# Supplementary material for: AAV-mediated FGF21 gene therapy promotes health span extension by whole-body tissue-specific adaptations
Source: Mol Ther. 2026 Jun 2;34(8):4546–68. doi: 10.1016/j.ymthe.2026.05.025 (PMC13464179; doi:10.1016/j.ymthe.2026.05.025)
Supplement: Supplementary file 1 [file mmc1.pdf]

YMTHE, Volume *34*

Supplemental Information

AAV-mediated FGF21 gene therapy promotes health span extension by whole-body tissue-specific adaptations

Veronica Jimenez, Victor Sacristan, Miquel Garcia, Claudia Jambrina, Estefania Casana, Sergio Muñoz, Laia Vilà, Ignasi Grass, Maria Luisa Jaén, Carles Roca, Xavier León, Sara Marcó, Maria Molas, Albert Ribera, Ivet Elias, Jordi Rodó, Tura Ferré, and Fatima Bosch

Figure S1

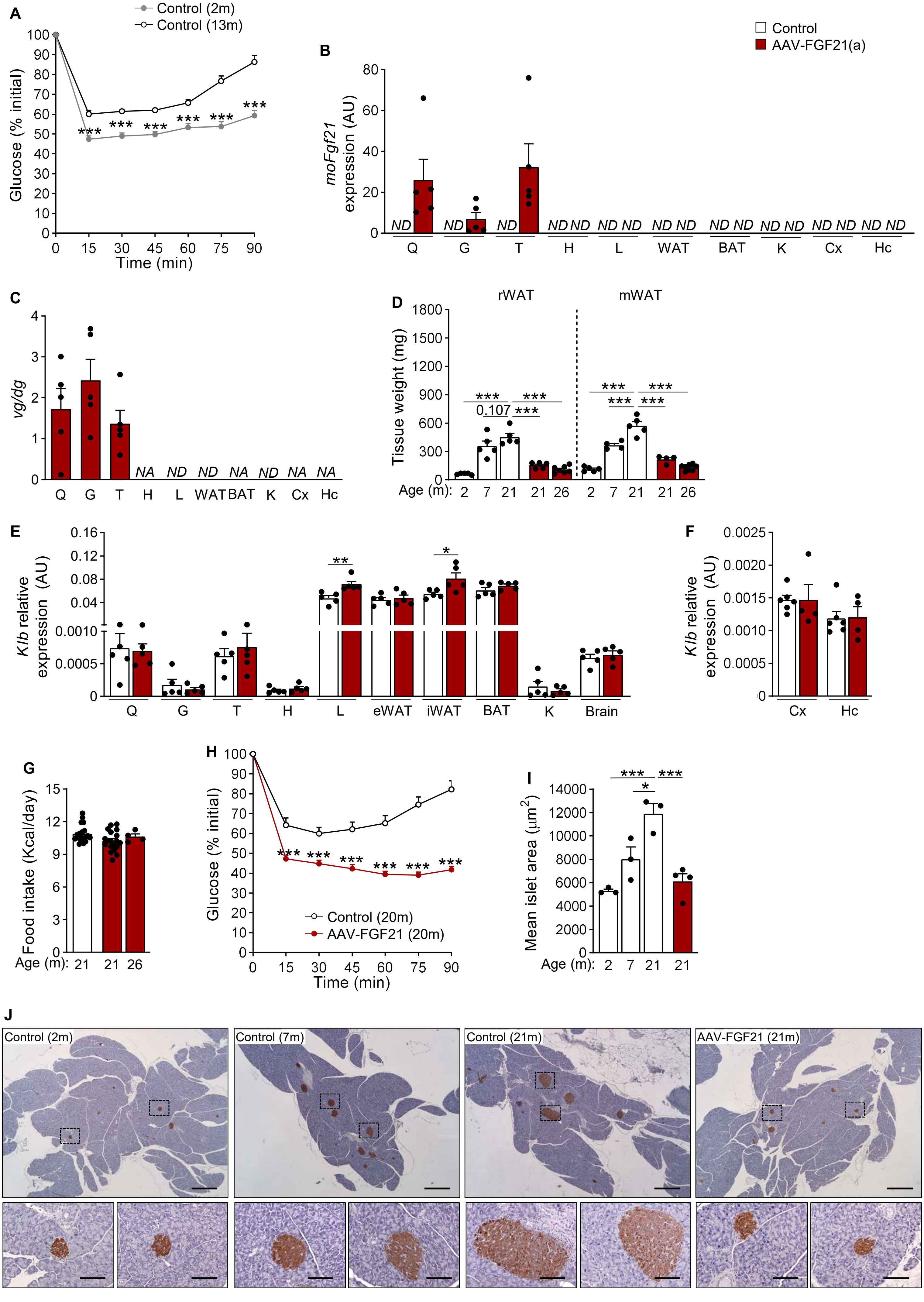
A
Control (2m)
Control (13m)
Glucose (% initial)
Time (min)
B
Control
AAV-FGF21(a)
moFgf21 expression (AU)
Q G T H L WAT BAT K Cx Hc
ND
C
vg/dg
NA
ND
D
rWAT
mWAT
Tissue weight (mg)
Age (m):
0.107
E
Klb relative expression (AU)
Q G T H L eWAT iWAT BAT K Brain
F
Cx Hc
G
Food intake (Kcal/day)
H
Control (20m)
AAV-FGF21 (20m)
I
Mean islet area (μm²)
J
Control (2m)
Control (7m)
Control (21m)
AAV-FGF21 (21m)

Figure S1. Effects of AAV-FGF21 treatment in body weight gain and glucose metabolism in male mice. (**A**) Insulin sensitivity was determined after an intraperitoneal injection of insulin (0.75 units/kg body weight) in 2- and 13-month-old male mice. Results were calculated as the percentage of initial blood glucose levels (*n*=11-30/group). (**B**) Quantification by qRT–PCR of the expression of murine optimized Fgf21 (*moFgf21*) in the three injected skeletal muscles (Q, quadriceps; G, gastrocnemius; T, tibialis cranialis) and in the heart (H), the liver (L), white adipose tissue (WAT), brown adipose tissue (BAT), and kidney (K) in 21-month-old male mice and in cortex (Cx) and hippocampus (Hc) in 26-month-old male mice (*n*=5/group). (**C**) Vector genome copy number in the same cohorts as in (B) (*n*=5/group).(**D**) Weight of the retroperitoneal (rWAT), and mesenteric (mWAT) white adipose tissue depots in male mice (*n*=5-8/group). (**E** and **F**) Quantification by qRT–PCR of the expression of β-Klotho (*Klb*) in the same cohorts as in B and C in 21 (E) and 26-month-old (F) male mice (*n*=5/group). (**G**) Histogram depicting the food intake in male mice (*n*=4-19 cages/group). (**H**) Insulin sensitivity was determined after an intraperitoneal injection of insulin (0.75 units/kg body weight) in 21-month-old male mice. Results were calculated as the percentage of initial blood glucose levels (*n*=13-19/group). (**I**) Representative images of the immunostaining against insulin in pancreas sections of male mice. Scale bars: 400 μm. Inset scale bars: 100 μm. (**J**) Quantification of mean islet area in male mice (*n*=3-4/group). Data are expressed as the mean ± SEM. *P<0.05, **P<0.01, ***P<0.001 by one-way ANOVA with Dunnet's multiple comparison test (D,G,I) or Student's two-tailed t test (A,E,F,H). AAV-FGF21 (a), male mice were treated with AAV-FGF21 vectors at 13 months of age; AU, arbitrary units; m, months of age; ND, non-detected; NA, non-available; vg/dg, vector genomes/diploid genome.

Figure S2

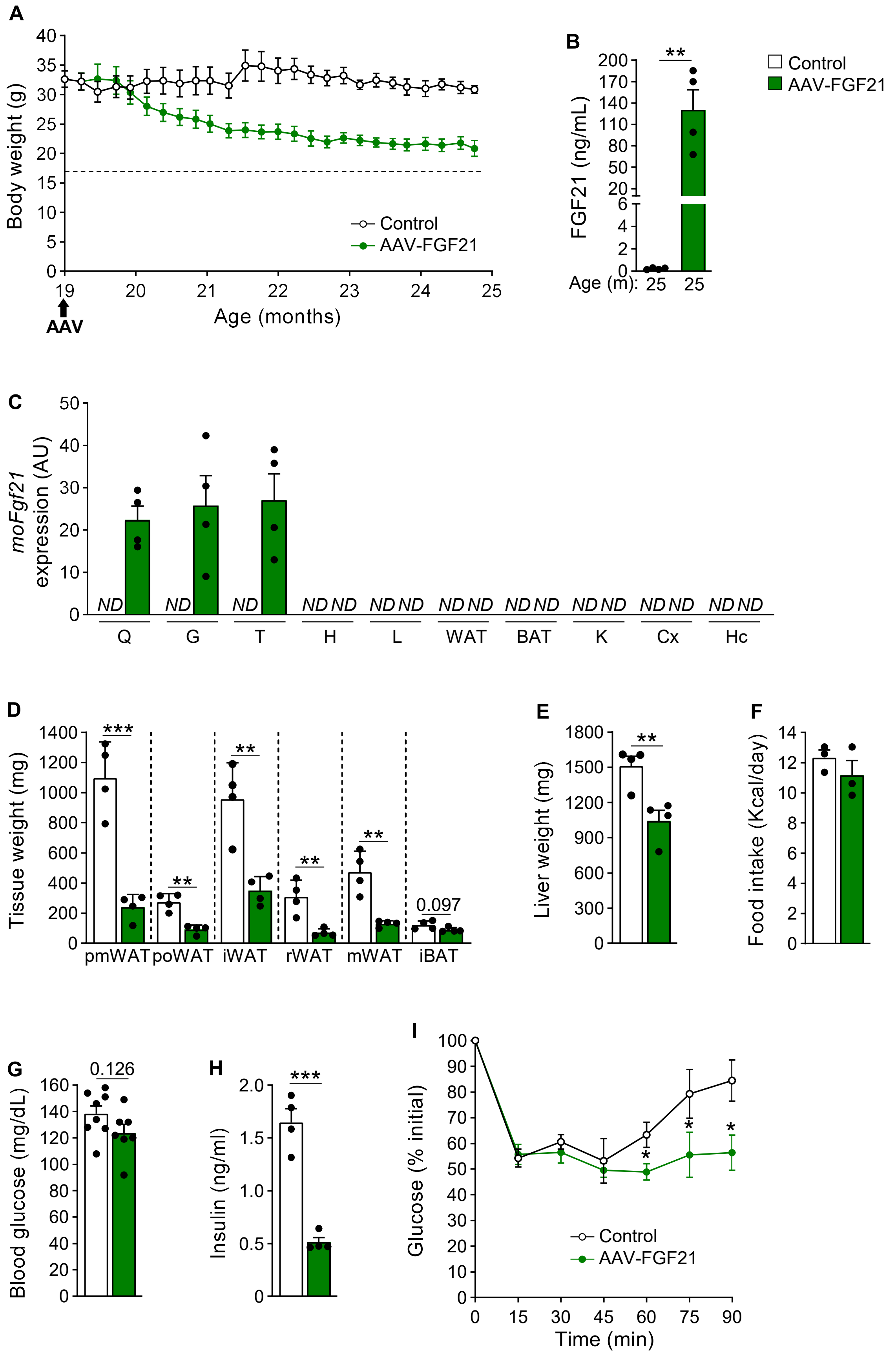
A
Body weight (g)
Age (months)
AAV
Control
AAV-FGF21
B
FGF21 (ng/mL)
Age (m): 25 25
**
Control
AAV-FGF21
C
moFgf21 expression (AU)
ND
Q
G
T
H
L
WAT
BAT
K
Cx
Hc
D
Tissue weight (mg)
pmWAT
poWAT
iWAT
rWAT
mWAT
iBAT
0.097
E
Liver weight (mg)
F
Food intake (Kcal/day)
G
Blood glucose (mg/dL)
0.126
H
Insulin (ng/ml)
I
Glucose (% initial)
Time (min)
Control
AAV-FGF21

Figure S2. Effects of AAV-FGF21 treatment in body weight gain and glucose metabolism in female mice. (**A**) Body weight follow-up in female mice treated with AAV-FGF21 vectors at 19 months of age (*n*=7-8/group). (**B**) Serum FGF21 levels in female mice (*n*=4/group). (**C**) Quantification by qRT–PCR of the expression of murine optimized Fgf21 (*moFgf21*) in the three injected skeletal muscles and in the heart (H), the liver (L), white adipose tissue (WAT), brown adipose tissue (BAT), kidney (K), cortex (Cx) and hippocampus (Hc) in 25-month-old female mice (*n*=4/group). (**D**) Weight of the parametrial (pmWAT), periovaric (poWAT), inguinal (iWAT), retroperitoneal (rWAT) and mesenteric (mWAT) white adipose tissue depots and the interscapular brown adipose tissue depot (iBAT) in female mice (*n*=4/group). (**E**) Weight of the liver in female mice (*n*=4/group). (**F**) Histogram depicting the food intake in female mice (*n*=3 cages/group). (**G** and **H**) Fed blood glucose (H, n=7-8/group) and insulin (**I**, n=4/group) levels in female mice at 21 months of age. (**I**) Insulin sensitivity was determined after an intraperitoneal injection of insulin (0.375 units/kg body weight) in 21-month-old female mice. Results were calculated as the percentage of initial blood glucose levels (*n*=6/group). Data are expressed as the mean ± SEM. *P<0.05, **P<0.01, ***P<0.001 by Student's two-tailed t test. AU, arbitrary units; m, months of age; ND, non-detected.

Figure S3

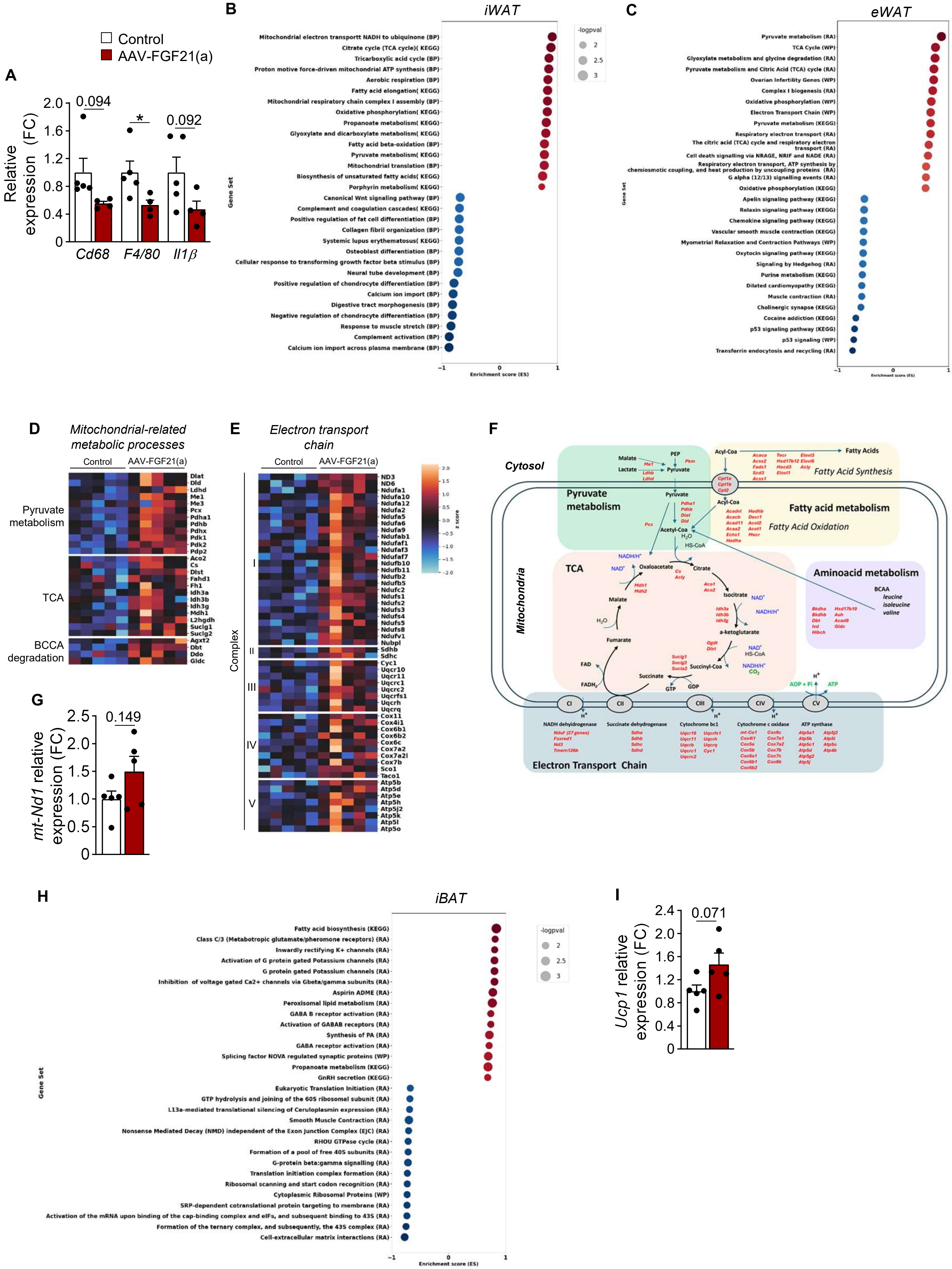

A
Control
AAV-FGF21(a)
Relative expression (FC)
0.094
*
0.092
Cd68
F4/80
Il1β
B
iWAT
Mitochondrial electron transportt NADH to ubiquinone (BP)
Citrate cycle (TCA cycle)(KEGG)
Tricarboxylic acid cycle (BP)
Proton motive force-driven mitochondrial ATP synthesis (BP)
Aerobic respiration (BP)
Fatty acid elongation(KEGG)
Mitochondrial respiratory chain complex I assembly (BP)
Oxidative phosphorylation(KEGG)
Propanoate metabolism(KEGG)
Glyoxylate and dicarboxylate metabolism(KEGG)
Fatty acid beta-oxidation (BP)
Pyruvate metabolism(KEGG)
Mitochondrial translation (BP)
Biosynthesis of unsaturated fatty acids(KEGG)
Porphyrin metabolism(KEGG)
Canonical Wnt signaling pathway (BP)
Complement and coagulation cascades(KEGG)
Positive regulation of fat cell differentiation (BP)
Collagen fibril organization (BP)
Systemic lupus erythematosus(KEGG)
Osteoblast differentiation (BP)
Cellular response to transforming growth factor beta stimulus (BP)
Neural tube development (BP)
Positive regulation of chondrocyte differentiation (BP)
Calcium ion import (BP)
Digestive tract morphogenesis (BP)
Negative regulation of chondrocyte differentiation (BP)
Response to muscle stretch (BP)
Complement activation (BP)
Calcium ion import across plasma membrane (BP)
Gene Set
Enrichment score (ES)
-logpval
C
eWAT
Pyruvate metabolism (RA)
TCA Cycle (WP)
Glyoxylate metabolism and glycine degradation (RA)
Pyruvate metabolism and Citric Acid (TCA) cycle (RA)
Ovarian Infertility Genes (WP)
Complex I biogenesis (RA)
Oxidative phosphorylation (WP)
Electron Transport Chain (WP)
Pyruvate metabolism (KEGG)
Respiratory electron transport (RA)
The citric acid (TCA) cycle and respiratory electron transport (RA)
Cell death signalling via NRAGE, NRIF and NADE (RA)
Respiratory electron transport, ATP synthesis by chemiosmotic coupling, and heat production by uncoupling proteins (RA)
G alpha (12/13) signalling events (RA)
Oxidative phosphorylation (KEGG)
Apelin signaling pathway (KEGG)
Relaxin signaling pathway (KEGG)
Chemokine signaling pathway (KEGG)
Vascular smooth muscle contraction (KEGG)
Myometrial Relaxation and Contraction Pathways (WP)
Oxytocin signaling pathway (KEGG)
Signaling by Hedgehog (RA)
Purine metabolism (KEGG)
Dilated cardiomyopathy (KEGG)
Muscle contraction (RA)
Cholinergic synapse (KEGG)
Cocaine addiction (KEGG)
p53 signaling pathway (KEGG)
p53 signaling (WP)
Transferrin endocytosis and recycling (RA)
D
Mitochondrial-related metabolic processes
Control
AAV-FGF21(a)
Pyruvate metabolism
TCA
BCCA degradation
E
Electron transport chain
Complex
F
Cytosol
Mitochondria
Pyruvate metabolism
Fatty acid metabolism
Fatty Acid Synthesis
Fatty Acid Oxidation
TCA
Aminoacid metabolism
Electron Transport Chain
G
mt-Nd1 relative expression (FC)
0.149
H
iBAT
Fatty acid biosynthesis (KEGG)
Class C/3 (Metabotropic glutamate/pheromone receptors) (RA)
Inwardly rectifying K+ channels (RA)
Activation of G protein gated Potassium channels (RA)
G protein gated Potassium channels (RA)
Inhibition of voltage gated Ca2+ channels via Gbeta/gamma subunits (RA)
Aspirin ADME (RA)
Peroxisomal lipid metabolism (RA)
GABA B receptor activation (RA)
Activation of GABAB receptors (RA)
Synthesis of PA (RA)
GABA receptor activation (RA)
Splicing factor NOVA regulated synaptic proteins (WP)
Propanoate metabolism (KEGG)
GnRH secretion (KEGG)
Eukaryotic Translation Initiation (RA)
GTP hydrolysis and joining of the 60S ribosomal subunit (RA)
L13a-mediated translational silencing of Ceruloplasmin expression (RA)
Smooth Muscle Contraction (RA)
Nonsense Mediated Decay (NMD) independent of the Exon Junction Complex (EJC) (RA)
RHOU GTPase cycle (RA)
Formation of a pool of free 40S subunits (RA)
G-protein beta:gamma signalling (RA)
Translation initiation complex formation (RA)
Ribosomal scanning and start codon recognition (RA)
Cytoplasmic Ribosomal Proteins (WP)
SRP-dependent cotranslational protein targeting to membrane (RA)
Activation of the mRNA upon binding of the cap-binding complex and eIFs, and subsequent binding to 43S (RA)
Formation of the ternary complex, and subsequently, the 43S complex (RA)
Cell-extracellular matrix interactions (RA)
Gene Set
Enrichment score (ES)
-logpval
I
Ucp1 relative expression (FC)
0.071

Figure S3. Effects of treatment with AAV-FGF21 in adipose tissue in male mice. Male mice were treated with either AAV-Null (control) or AAV-FGF21 vectors at 13 months of age (AAV-FGF21 (a)). (**A**) Quantification by qRT–PCR of the expression of the inflammatory markers *Cd68*, *F4/80* and *Il1β* in iWAT at 21 months of age (n=5/group). (**B** and **C**) The plots represent the gene sets identified through Gene Set Enrichment Analysis (GSEA) of iWAT (B) and eWAT (C) of AAV-Null and AAV-FGF21-treated mice at 21 months of age. The color gradient from blue to red signifies the enrichment score (ES) values, providing a visual representation of enrichment magnitude. (**D** and **E**) The heatmaps depict the normalized expression patterns of leading-edge genes involved in mitochondrial-related metabolic processes (D) and encoding electron transport chain components (E) in eWAT at 21 months of age. (**F**) Scheme depicting the upregulated leading edge-genes (highlighted in red) involved in mitochondrial-related metabolism in iWAT of 21-month-old AAV-FGF21-treated mice. (**G**) Quantification by qRT–PCR of the expression of the mitochondrial gene mt-*Nd1* encoding the subunit 1 of NADH dehydrogenase in iWAT of 21-month-old mice (*n*=5/group). (**H**) The plot represents the gene sets identified through Gene Set Enrichment Analysis (GSEA) of iBAT of AAV-Null and AAV-FGF21-treated mice at 21 months of age. The color gradient from blue to red signifies the enrichment score (ES) values, providing a visual representation of enrichment magnitude. (**I**) Quantification by qRT–PCR of the expression of the thermogenic marker *Ucp1* in iBAT of 21-month-old mice (*n*=5/group). In (A,G,I) data are expressed as the mean ± SEM. FC, fold change. *P<0.05 by Student's two-tailed t test.

Figure S4

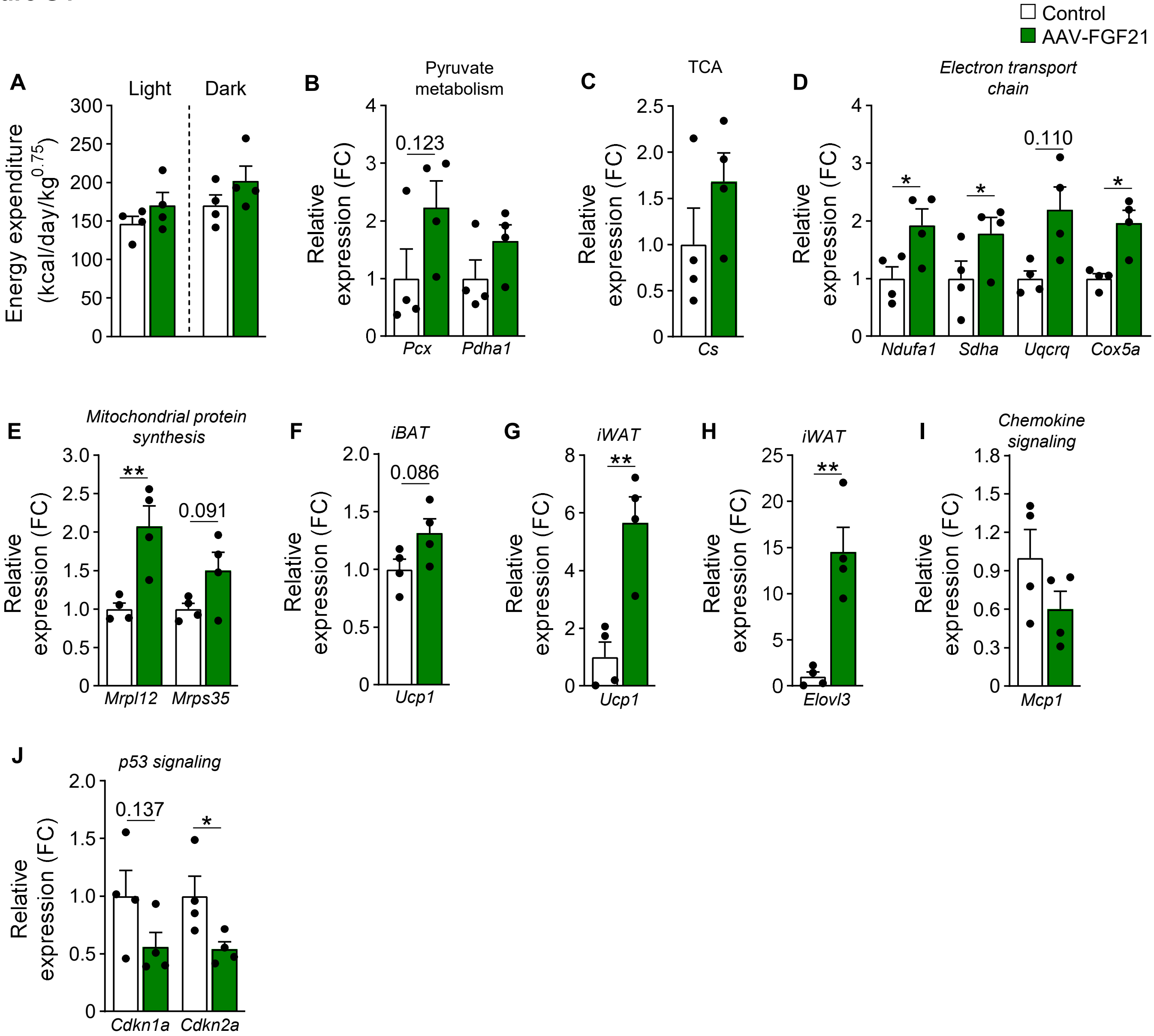

Control
AAV-FGF21
A
Light
Dark
Energy expenditure (kcal/day/kg$^{0.75}$)
300
250
200
150
100
50
0
B
Pyruvate metabolism
Relative expression (FC)
0.123
Pcx
Pdha1
C
TCA
Cs
D
Electron transport chain
0.110
*
Ndufa1
Sdha
Uqcrq
Cox5a
E
Mitochondrial protein synthesis
**
0.091
Mrpl12
Mrps35
F
iBAT
0.086
Ucp1
G
iWAT
**
Ucp1
H
iWAT
**
Elovl3
I
Chemokine signaling
Mcp1
J
p53 signaling
0.137
*
Cdkn1a
Cdkn2a

Figure S4. Effects of treatment with AAV-FGF21 in adipose tissue in female mice. (**A**) Energy expenditure was measured with an indirect open circuit calorimeter in female mice treated with AAV-FGF21 vectors at 19 months of age (*n*=4/group). (**B-E**) Quantification by qRT–PCR of the expression of key genes involved in pyruvate metabolism (B), the tricarboxylic acid cycle (TCA) (C), electron transport chain and mitochondrial protein synthesis (D) in iWAT in 25-month-old female mice (*n*=4/group). (**F** and **G**) Quantification by qRT–PCR of the expression of the thermogenic gene marker *Ucp1* in iBAT (F) and iWAT (G) in 25-month-old female mice (*n*=4/group). (**H**) Quantification by qRT–PCR of the expression of *Elovl3* in iWAT in 25-month-old female mice (*n*=4/group). (**I** and **J**) Quantification by qRT–PCR of the expression of key genes involved in chemokine (I) and p53 signaling (J) pathways in pmWAT in 25-month-old female mice (*n*=4/group). Data are expressed as the mean ± SEM. FC, fold change. *$P<0.05$, **$P<0.01$ by Student's two-tailed t test.

Figure S5

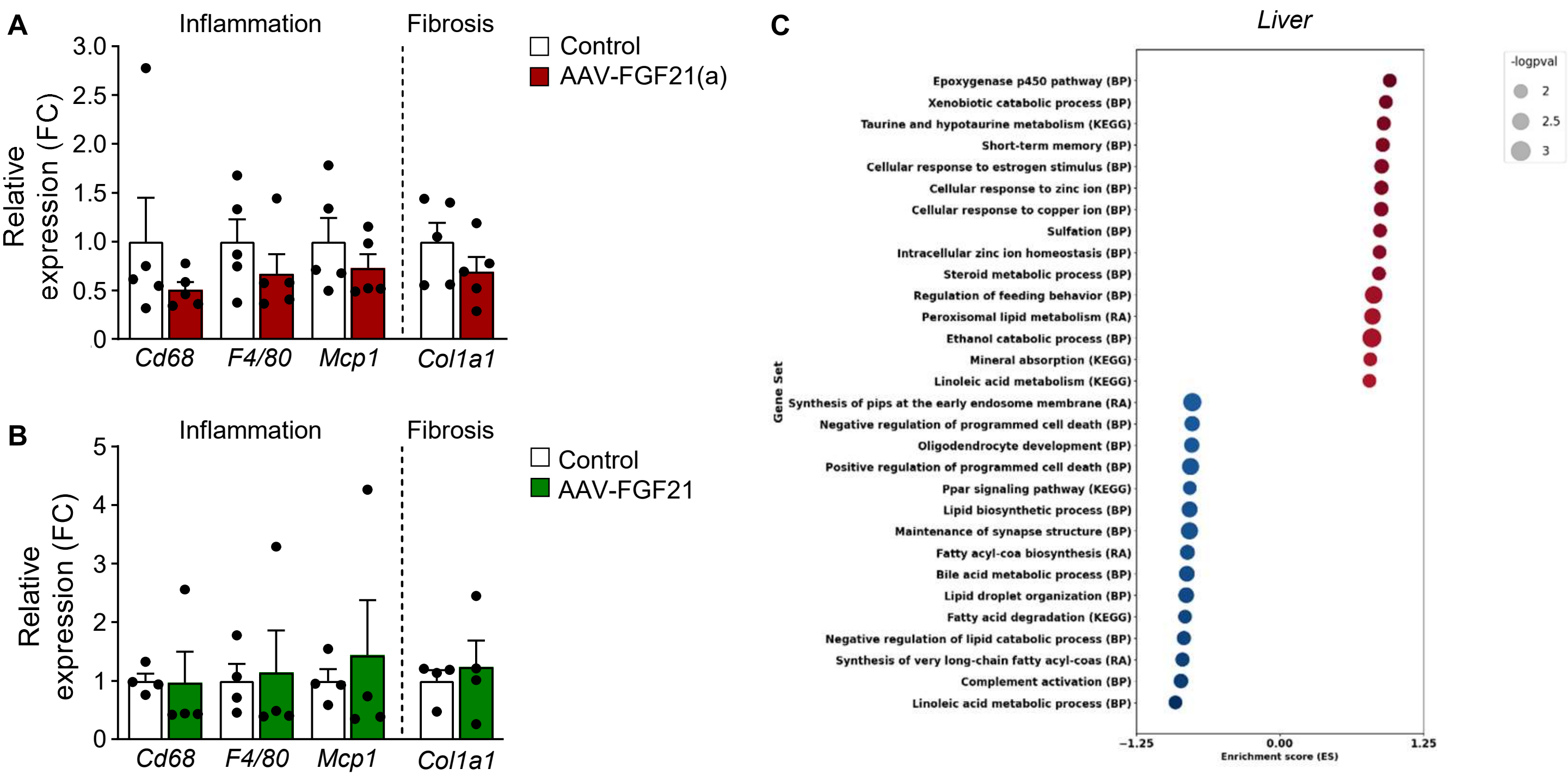

Figure S5. Effects of treatment with AAV-FGF21 in the liver. Male and female mice were treated with either AAV-Null (control) or AAV-FGF21 vectors at 13 (AAV-FGF21 (a)) or 19 months of age (AAV-FG21), respectively. (**A and B**) Quantification by qRT–PCR of the expression of the inflammatory and fibrosis markers *Cd68, F4/80*, *Mcp1* and *Col1a1* in the liver of male mice at 21 months of age (*n*=5/group) (A) or of female mice at 25 months of age (*n*=4/group) (B). (**C**) The plot represents the gene sets identified through Gene Set Enrichment Analysis (GSEA) of the liver of AAV-Null and AAV-FGF21-treated male mice at 21 months of age. The color gradient from blue to red signifies the ES values, providing a visual representation of enrichment magnitude. In (A,B) data are expressed as the mean ± SEM. FC, fold change.

Figure S6

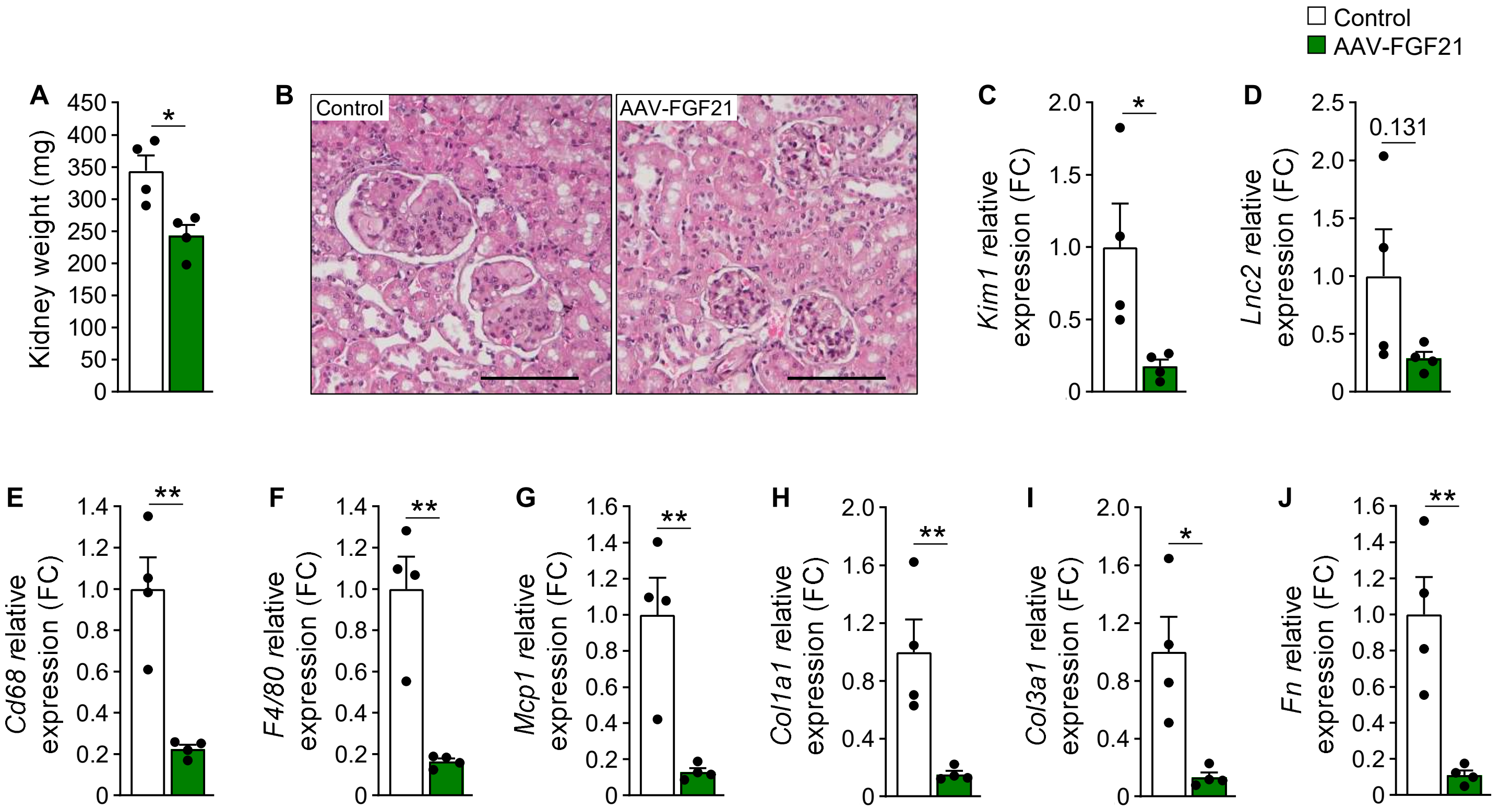

Figure S6. AAV-FGF21 treatment revers age-related kidney disease in female mice. Female mice were treated with AAV-FGF21 vectors at 19 (AAV-FGF21) months of age. (**A**) Kidney weight (n=4/group). (**B**) Representative images of hematoxylin–eosin staining of kidney sections. Scale bars, 100 μm. (**C** and **D**) Quantification by qRT–PCR of the expression of the kidney damage gene markers *Kim1* (C) and *Lnc2* (D) (n=4/group). (**E** to **J**) Quantification by qRT–PCR of the expression of the inflammatory and fibrosis gene markers *Cd68* (E), *F4/80* (F), *Mcp1* (G), *Col1a1* (H), *Col3a1* (I) and *Fn* (J) (n=4/group). Data are expressed as the mean ± SEM. *P<0.05, **P<0.01 by Student's two-tailed t test. FC, Fold change.

Figure S7

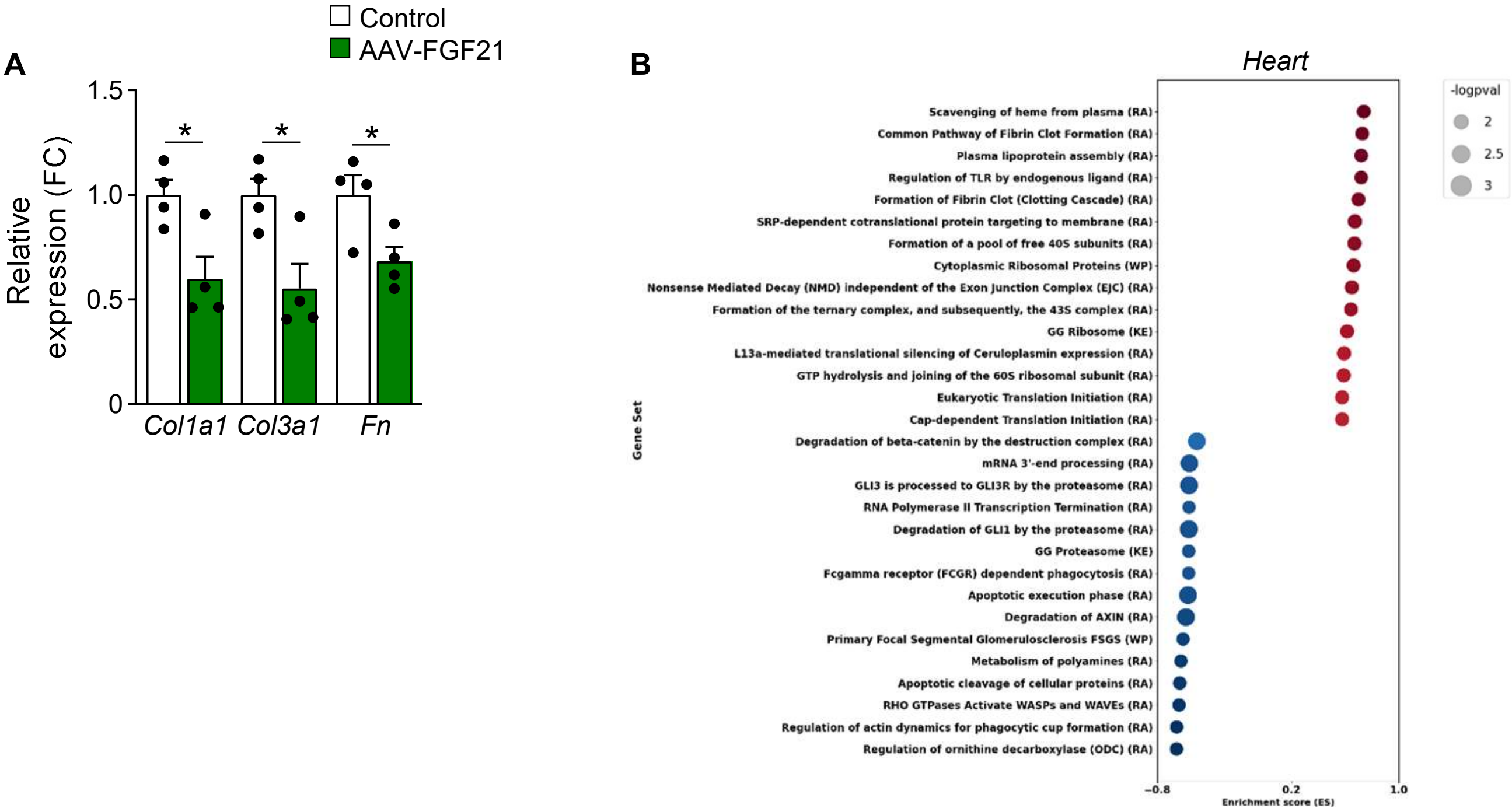

Figure S7. Effects of treatment with AAV-FGF21 in the heart. (**A**) Quantification by qRT–PCR of the expression of the fibrotic gene markers *Col1a1*, Col3a1, and *Fn*, (n=4/group) in female mice treated with AAV-FGF21 vectors at 19 months of age and sacrificed at 25 months of age. (**B**) The plot represents the gene sets identified through Gene Set Enrichment Analysis (GSEA) of the heart of AAV-Null and AAV-FGF21-treated mice at 21 months of age. The color gradient from blue to red signifies the ES values, providing a visual representation of enrichment magnitude. In (A) data are expressed as the mean ± SEM. *P<0.05 by Student's two-tailed t test. FC, Fold change.

Figure S8

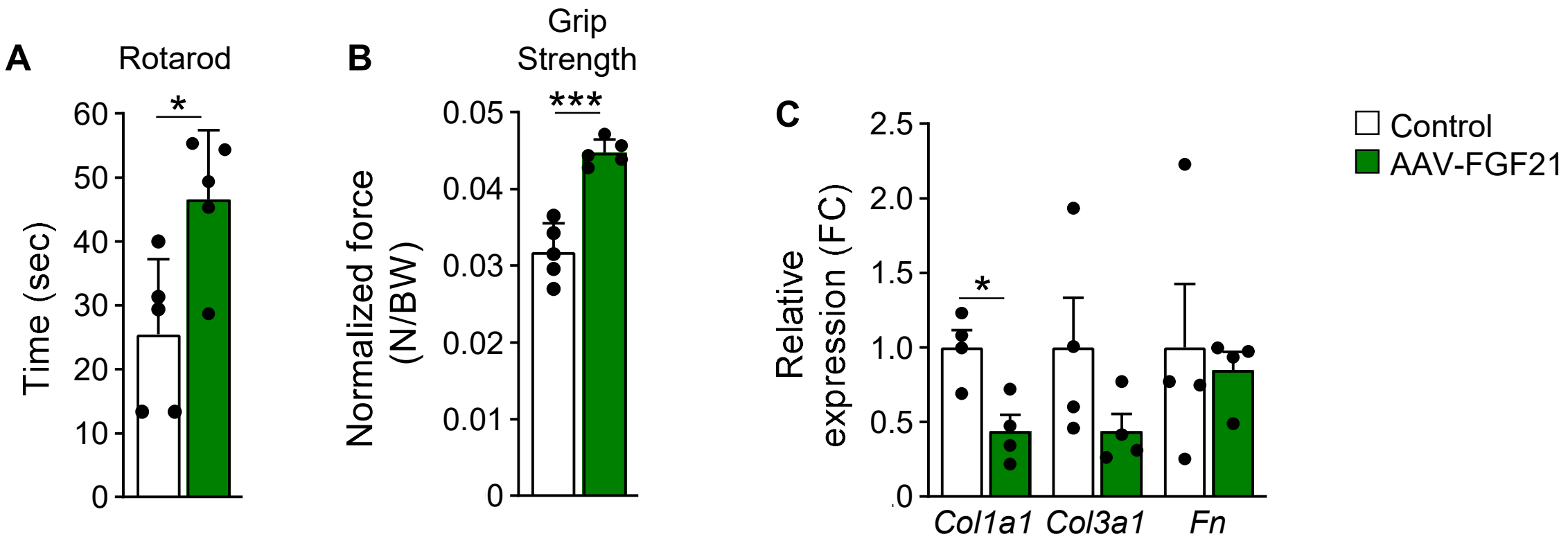

Figure S8. AAV1-FGF21 therapy improves muscular performance in female mice. (**A**) Time before falling during the rotarod test (*n*=5/group). (**B**) Forelimb grip strength (*n*=5/group). (**C**) Quantification by qRT–PCR of the expression of the fibrotic markers *Col1a1*, Col3a1, and *Fn* (n=4/group). Data are expressed as the mean ± SEM. *P<0.05, ***P<0.001 by Student's two-tailed t test.

Figure S9

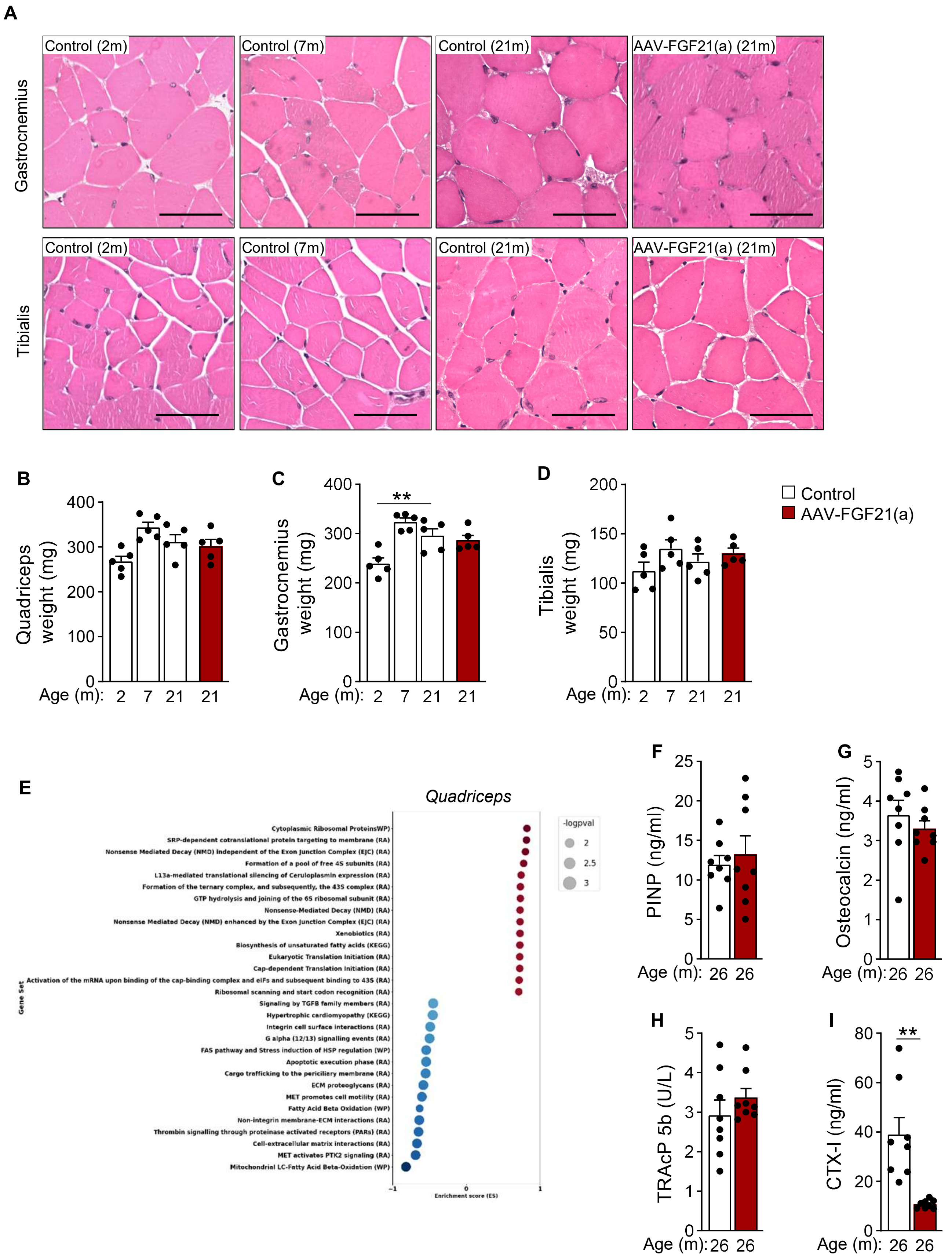
A
Gastrocnemius
Tibialis
Control (2m)
Control (7m)
Control (21m)
AAV-FGF21(a) (21m)
B
Quadriceps weight (mg)
Age (m): 2 7 21 21
C
Gastrocnemius weight (mg)
**
Age (m): 2 7 21 21
D
Tibialis weight (mg)
Age (m): 2 7 21 21
Control
AAV-FGF21(a)
E
Quadriceps
-logpval
Gene Set
Cytoplasmic Ribosomal ProteinsWP)
SRP-dependent cotranslational protein targeting to membrane (RA)
Nonsense Mediated Decay (NMD) independent of the Exon Junction Complex (EJC) (RA)
Formation of a pool of free 4S subunits (RA)
L13a-mediated translational silencing of Ceruloplasmin expression (RA)
Formation of the ternary complex, and subsequently, the 43S complex (RA)
GTP hydrolysis and joining of the 6S ribosomal subunit (RA)
Nonsense-Mediated Decay (NMD) (RA)
Nonsense Mediated Decay (NMD) enhanced by the Exon Junction Complex (EJC) (RA)
Xenobiotics (RA)
Biosynthesis of unsaturated fatty acids (KEGG)
Eukaryotic Translation Initiation (RA)
Cap-dependent Translation Initiation (RA)
Activation of the mRNA upon binding of the cap-binding complex and eIFs and subsequent binding to 43S (RA)
Ribosomal scanning and start codon recognition (RA)
Signaling by TGFB family members (RA)
Hypertrophic cardiomyopathy (KEGG)
Integrin cell surface interactions (RA)
G alpha (12/13) signalling events (RA)
FAS pathway and Stress induction of HSP regulation (WP)
Apoptotic execution phase (RA)
Cargo trafficking to the periciliary membrane (RA)
ECM proteoglycans (RA)
MET promotes cell motility (RA)
Fatty Acid Beta Oxidation (WP)
Non-integrin membrane-ECM interactions (RA)
Thrombin signalling through proteinase activated receptors (PARs) (RA)
Cell-extracellular matrix interactions (RA)
MET activates PTK2 signaling (RA)
Mitochondrial LC-Fatty Acid Beta-Oxidation (WP)
Enrichment score (ES)
F
PINP (ng/ml)
Age (m): 26 26
G
Osteocalcin (ng/ml)
Age (m): 26 26
H
TRAcP 5b (U/L)
Age (m): 26 26
I
CTX-I (ng/ml)
**
Age (m): 26 26

Figure S9. Effects of treatment with AAV-FGF21 in the skeletal muscle and bones. Male mice were treated with either AAV-Null (control) or AAV-FGF21 vectors at 13 months of age (AAV-FGF21 (a)). (**A**) Representative images of hematoxylin–eosin staining of gastrocnemius and tibialis cranialis sections. Scale bars, 100 μm. (**B**-**D**) Weight of the quadriceps (B), gastrocnemius (C) and tibialis cranialis (D) skeletal muscles (*n*=5/group). (**E**) The plot represents the gene sets identified through Gene Set Enrichment Analysis (GSEA) of the quadriceps of AAV-Null and AAV-FGF21-treated mice at 21 months of age. The color gradient from blue to red signifies the ES values, providing a visual representation of enrichment magnitude. (**F** and **G**) Serum levels of the bone formation markers procollagen type 1 N-terminal propeptide (PINP) (F) and osteocalcin (G) in 26-month-old male mice. (**H** and **I**) Serum levels of the bone resorption markers tartrate-resistant acid phosphatase 5b (TRAcP 5b) (H) and circulating carboxy-terminal crosslinked telopeptide of type 1 collagen (CTX-1) (**I**) in 26-month-old male mice. In (B-D and F-**I**) data are expressed as the mean ± SEM. **$P<0.01$ by one-way ANOVA with Dunnet's multiple comparison test (B-D) or by Student's two-tailed t test (F-**I**). m, months of age.

Figure S10

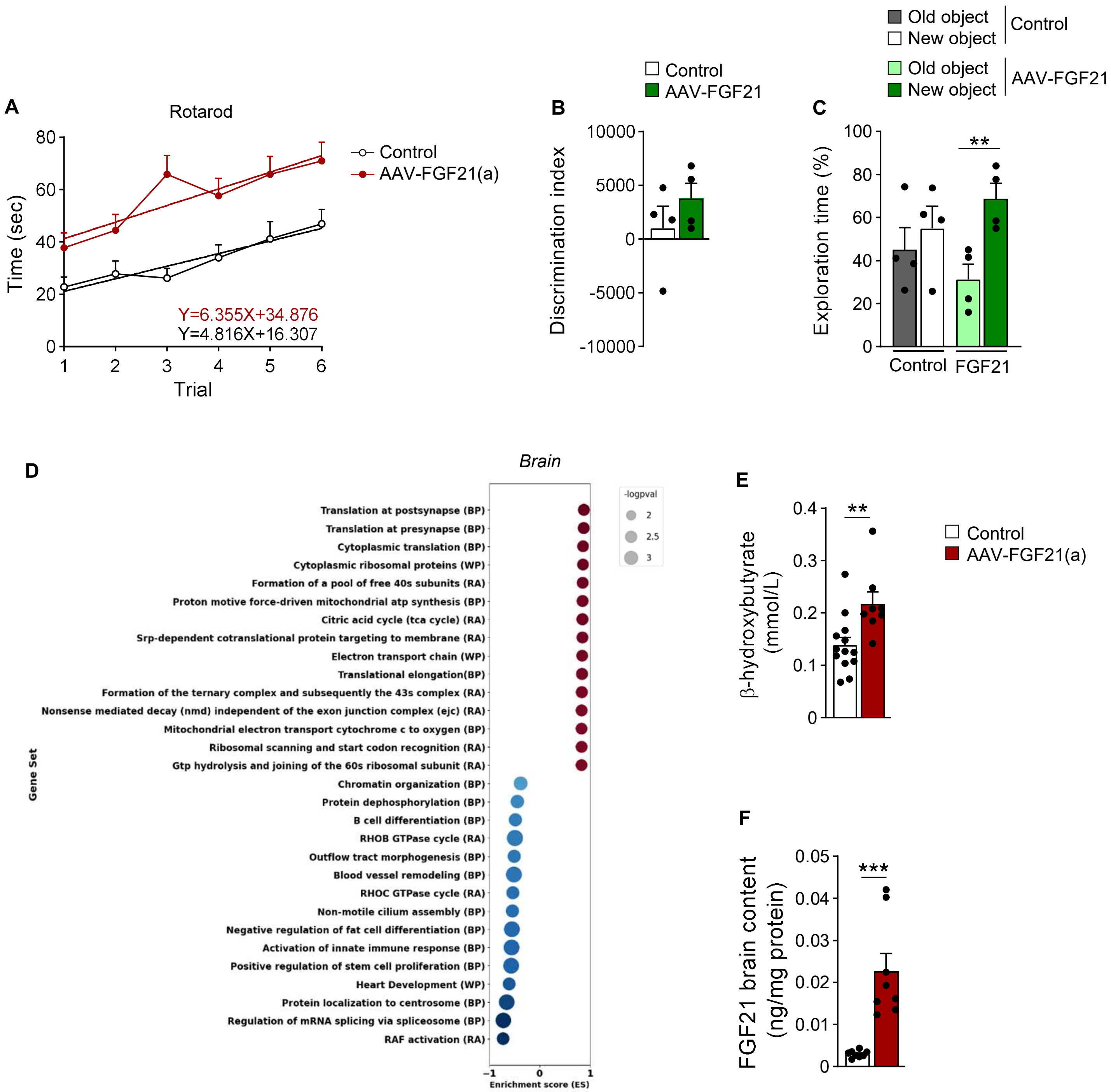

A
Rotarod
Time (sec)
Trial
Control
AAV-FGF21(a)
Y=6.355X+34.876
Y=4.816X+16.307
B
Control
AAV-FGF21
Discrimination index
C
Old object
New object
Control
Old object
New object
AAV-FGF21
Exploration time (%)
**
Control
FGF21
D
Brain
-logpval
Translation at postsynapse (BP)
Translation at presynapse (BP)
Cytoplasmic translation (BP)
Cytoplasmic ribosomal proteins (WP)
Formation of a pool of free 40s subunits (RA)
Proton motive force-driven mitochondrial atp synthesis (BP)
Citric acid cycle (tca cycle) (RA)
Srp-dependent cotranslational protein targeting to membrane (RA)
Electron transport chain (WP)
Translational elongation(BP)
Formation of the ternary complex and subsequently the 43s complex (RA)
Nonsense mediated decay (nmd) independent of the exon junction complex (ejc) (RA)
Mitochondrial electron transport cytochrome c to oxygen (BP)
Ribosomal scanning and start codon recognition (RA)
Gtp hydrolysis and joining of the 60s ribosomal subunit (RA)
Chromatin organization (BP)
Protein dephosphorylation (BP)
B cell differentiation (BP)
RHOB GTPase cycle (RA)
Outflow tract morphogenesis (BP)
Blood vessel remodeling (BP)
RHOC GTPase cycle (RA)
Non-motile cilium assembly (BP)
Negative regulation of fat cell differentiation (BP)
Activation of innate immune response (BP)
Positive regulation of stem cell proliferation (BP)
Heart Development (WP)
Protein localization to centrosome (BP)
Regulation of mRNA splicing via spliceosome (BP)
RAF activation (RA)
Gene Set
Enrichment score (ES)
E
β-hydroxybutyrate (mmol/L)
**
Control
AAV-FGF21(a)
F
FGF21 brain content (ng/mg protein)
***

Figure S10. Effects of treatment with AAV-FGF21 vectors in the brain. (**A**) Motor learning ability during subsequent trials in the rotarod test in male mice treated with either AAV-Null (control) or AAV-FGF21 vectors at 13 months of age (AAV-FGF21 (a)) (n=14-15/group). (**B** and **C**). Discrimination index (B) and exploration time (C) during the novel object recognition (NOR) test in female mice treated with AAV-FGF21 vectors at 19 months of age (n=4/group). (**D**) The plot represents the gene sets identified through Gene Set Enrichment Analysis (GSEA) of the brain of AAV-Null and AAV-FGF21-treated male mice at 21 months of age. The color gradient from blue to red signifies the ES values, providing a visual representation of enrichment magnitude. (**E**) Serum β-hydroxybutyrate levels in male mice at 26 months age (*n*=8-13/group). (**F**) FGF21 protein content in brain in male mice at 21 months of age (*n*=8/group). In (A,B,C,E,F) data are expressed as the mean ± SEM. $^{**}P<0.01$, $^{***}P<0.001$ by Student's two-tailed t test.

Table S1. Annotated sequence of the pAAV-CMV-moFGF21 plasmid.

Table S2. GSEA Leading Edge Analysis and Cluster Map across Tissues. The dataset presents the top 15 enriched gene sets for AAV-FGF21- (orange) and AAV-Null-treated (blue) mice, categorized by tissue type in individual sheets. For each gene set, the leading-edge genes (the subset driving the enrichment score) are identified. The accompanying binary cluster maps (overlap matrices) visualize the distribution of these genes (x-axis) across the enriched pathways (y-axis). Yellow blocks indicate gene presence within a pathway's leading edge, while purple indicates absence. This matrix identifies "hub" genes contributing to multiple biological processes and highlights functional redundancies between enriched gene sets, facilitating a modular understanding of the FGF21-induced transcriptional response.
