# Supplementary material for: AAV-mediated FGF21 gene therapy promotes health span extension by whole-body tissue-specific adaptations
Source: Mol Ther. 2026 Jun 2;34(8):4546–68. doi: 10.1016/j.ymthe.2026.05.025 (PMC13464179; doi:10.1016/j.ymthe.2026.05.025)
Supplement: Supplementary file 2 [file mmc4.pdf]

Molecular Therapy
Original Article

AAV-mediated FGF21 gene therapy promotes health span extension by whole-body tissue-specific adaptations

Veronica Jimenez,[1,2,3,4] Victor Sacristan,[1,2,3,4] Miquel Garcia,[1,2,3,4] Claudia Jambrina,[1,2,3] Estefania Casana,[1,2,3] Sergio Muñoz,[1,2,3] Laia Vilà,[1,2] Ignasi Grass,[1,2] Maria Luisa Jaén,[1,2,3] Carles Roca,[1,2,3] Xavier León,[1,2,3] Sara Marcó,[1,2,3] Maria Molas,[1,2,3] Albert Ribera,[1,2,3] Ivet Elias,[1,2,3] Jordi Rodó,[1,2] Tura Ferré,[1,2,3] and Fatima Bosch[1,2,3]

[1]Center of Animal Biotechnology and Gene Therapy (CBATEG), Universitat Autònoma de Barcelona, 08193 Bellaterra, Spain; [2]Department of Biochemistry and Molecular Biology, School of Veterinary Medicine, Universitat Autònoma de Barcelona, 08193 Bellaterra, Spain; [3]Centro de Investigación Biomédica en Red de Diabetes y Enfermedades Metabólicas Asociadas (CIBERDEM), Instituto de Salud Carlos III, 28029 Madrid, Spain

The decline of organ function during aging limits health span. Despite the potential of lifestyle interventions to improve health, sustained maintenance of health span is challenging, and no gerotherapeutic drugs have been approved. Here, we demonstrated that aged and geriatric male and female mice treated with muscle-directed adeno-associated viral (AAV) vector-mediated fibroblast growth factor 21 (FGF21) gene therapy extended health span and lifespan with sustained organ benefits. This treatment normalized body weight and adiposity, improved insulin sensitivity and glucose homeostasis, preserved hepatic detoxification capacity, counteracted age-related kidney disease, promoted cardiac health and muscular function, and enhanced cognition. Transcriptomic and histopathological analyses indicated improved whole-body energy homeostasis and cellular fitness, which were mediated by tissue-specific adaptations, including enhanced mitochondrial function, restored proteostasis, and reversion of inflammation, fibrosis, and amyloidosis. AAV-FGF21 treatment also activated AMPK signaling. These results highlight FGF21 gene therapy as a potential strategy to promote health span and delay age-related deterioration.

INTRODUCTION

Deterioration of physiological integrity and growing risk of disease during the aging process results from progressive impairment of cellular fitness and organ function.[1] Demographic projections indicate a dramatic expansion of the global aging population, with more than 2.1 billion individuals expected to be over 65 years old and 426 million over 80 by 2050 (www.who.int/z/news-room/fact-sheets/detail/ageing-and-health). Yet, while chronological lifespan continues to steadily increase, a comparable parallel prolongation of health span has not been achieved. Prevalence of age-associated disorders, such as type 2 diabetes (T2D), obesity, and cardiovascular or neurodegenerative diseases, is increasing with epidemic proportions (www.who.int/news-room/fact-sheets/detail/noncommunicable-diseases).

The aging process is accompanied by progressive metabolic dysregulation characterized by weight gain, increased adiposity, insulin resistance, and impaired cellular metabolism and energy homeostasis.[1–5] These metabolic shifts are recognized as central hallmarks of aging and key drivers of major age-related diseases, tissue functional decline, frailty, and diminished quality of life in the elderly.[2,3] Conversely, metabolically healthy individuals, centenarians, and long-lived populations demonstrate remarkable resistance to age-related metabolic deterioration.[3] They exhibit lower adiposity and improved insulin sensitivity, lipid metabolism, mitochondrial function, and protein homeostasis.[6–9]

Lifestyle interventions, such as exercise, healthy diet, caloric, protein, or amino acid restriction, and intermittent fasting, can enhance metabolic health, reduce chronic diseases, and promote health span.[10] These effects are mediated by a complex network of molecular pathways, in which AMPK, a master regulator of cellular metabolism,[11] is a critical regulatory hub.[10,12] However, these approaches show variable efficacy and suffer from poor long-term adherence. Recently, metabolic-targeting drugs like metformin and rapamycin have been proposed as potential gerotherapeutics. Metformin, a first-line treatment for T2D, mimics exercise and dietary interventions, extending health span in animals primarily through AMPK-dependent pathways.[13] Metformin also lowers the incidence of age-related diseases and reduces mortality in both diabetic and non-diabetic subjects.[14]

Similarly, the endocrine factor fibroblast growth factor 21 (FGF21) is a key metabolic regulator that coordinates inter-organ metabolism and energy homeostasis and has emerged as a promising therapeutic agent

Received 11 December 2025; accepted 27 May 2026;
https://doi.org/10.1016/j.ymthe.2026.05.025.

[4]These authors contributed equally

Correspondence: Fatima Bosch, Centre of Animal Biotechnology and Gene Therapy, Universitat Autònoma de Barcelona, 08193 Bellaterra, Spain..
E-mail: fatima.bosch@uab.es

 © 2026 The Author(s).
Published by Elsevier Inc. on behalf of The American Society of Gene and Cell Therapy.
This is an open access article under the CC BY license (http://creativecommons.org/licenses/by/4.0/).

for chronic metabolic diseases.[15] In this regard, one-time administration of adeno-associated viral (AAV) vector-mediated FGF21 gene therapy resulted in sustained reversal of metabolic dysfunction-associated steatohepatitis (MASH), T2D, and obesity in mice.[16,17] This gene therapy approach is advancing toward clinical translation in humans (https://kriyatherapeutics.com/pipeline/). In this study, the therapeutic benefits of the muscle-directed AAV-FGF21 gene therapy in old and geriatric (13-, 19-, and 22-month-old) male and female mice were assessed. This single intervention extended both health span and lifespan through targeted tissue adaptations that delay age-related whole-body organ deterioration. This study establishes AAV-FGF21 gene therapy as a translatable strategy to promote healthy aging with a favorable long-term efficacy and safety profile.

RESULTS

Treatment of old mice with AAV-FGF21 increases health span and lifespan

Overweight and insulin-resistant 13-month-old chow-fed male mice (Figure 1A; Figure S1A) were treated intramuscularly (i.m.) with 3×10^{11} viral genomes (vg) of AAV vectors of serotype 1 encoding FGF21 (AAV-FGF21) to assess whether this gene therapy could reverse established metabolic alterations and to prevent age-related decline in whole-body cellular fitness. While control (AAV-Null-treated) mice steadily increased their body weight, AAV-FGF21-treated mice progressively lost weight and reached a mean body weight similar to that of young (2-month-old) mice throughout the follow-up period (Figure 1A). At 21 months of age, obese AAV-Null-treated mice began losing weight and gradually increased mortality (Figures 1A and 1B). These mice showed a median lifespan of 28.14 months, which was expanded to 33.92 months in the AAV-FGF21-treated group ($p = 0.0098$), representing a 20.54% increase in midlife survival (Figure 1B). Moreover, by $\sim$34 months of age, 90% of AAV-Null mice had died (Figure 1B), whereas AAV-FGF21 treatment significantly extended longevity. Similarly, male mice treated with AAV-FGF21 at 22 months of age (Figure 1A) remained alive (4/4) at 26 months, while AAV-Null-treated mice showed increased mortality (8/31, $\sim$25.8%) during this 5-months period (Figure 1B). This benefit resulted from sustained elevation of circulating FGF21 levels (Figure 1C), due to specific transduction and FGF21 overexpression in the AAV-FGF21-treated skeletal muscles (Figures S1B and S1C). As a result of the i.m. administration of AAV1 vectors, vector genome copy numbers and AAV-derived FGF21 expression were not detected in other tissues (Figures S1B and S1C), consistent with previous reports.[16–19] The decreased body weight paralleled the reductions in the weight of the main white (WAT) and brown (BAT) adipose tissue depots, as well as the liver (Figures 1D and 1E; Figure S1D). These tissues also showed increased gene expression of the FGF21 co-receptor β-klotho (*Klb*) (Figures S1E and S1F). Although AAV-FGF21-treated mice were leaner, food intake remained unchanged (Figure S1G). Similar metabolic improvements were observed in both male and female geriatric mice, treated at 22 and 19 months old, respectively, with the same dose of AAV-FGF21 vectors (Figures 1A, 1C–1E; Figures S2A–S2F).

Moreover, AAV-FGF21 treatment decreased glycemia, normalized insulinemia, and increased insulin sensitivity (at both high and low insulin doses) and glucose tolerance, indicating improved peripheral tissue metabolism (Figures 1F–1I; Figure S1H). Consistent with insulin resistance, AAV-Null mice developed islet hyperplasia and increased β cell mass, both of which were normalized in AAV-FGF21-treated mice (Figure 1J; Figures S1I and S1J). AAV-FGF21-treated mice also exhibited reduced blood glucose levels in the pyruvate tolerance test (Figure 1K) reflecting decreased hepatic glucose production, which is typically elevated in insulin-resistant states. Similar insulin-sensitizing effects were observed in geriatric male and female mice treated with AAV-FGF21 vectors (Figures 1F and 1G; Figures S2G–S2I). Collectively, these results demonstrated that AAV-FGF21 treatment improved glucose homeostasis in aged mice. These whole-body benefits likely resulted from enhanced cellular fitness in key tissues.

Contribution of white and brown adipose tissues to extension of health span

The aging process exhibits organ-specific dynamics, with WAT showing the earliest onset and the most pronounced distress, particularly in metabolic and inflammation-related processes.[20,21] Consistently, old AAV-Null-treated animals exhibited increased lipid deposition in visceral (epididymal WAT, eWAT) and subcutaneous WAT (inguinal WAT, iWAT) as well as in BAT (Figures 2A and 2B). Conversely, aged AAV-FGF21-treated mice showed white adipocyte size comparable to that of 2-month-old controls (Figures 2A and 2B). Moreover, reduced expression of the macrophage markers protein Cd68 antigen (*Cd68*) and adhesion G protein-coupled receptor E1 (*Adgre1*, also known as *F4/80)*, along with the proinflammatory cytokine interleukin-1β (*IL1β*), was observed in eWAT and iWAT, indicating decreased age-associated low-grade inflammation (Figure 2C; Figure S3A). This paralleled the increased serum levels of adiponectin (Figure 2D), an anti-inflammatory and insulin-sensitizing adipokine, and a key biomarker for FGF21 biological activity.[22] The reduction of inflammation in adipose tissue and increased adiponectin levels would contribute to prevent systemic low-grade inflammation and development of insulin resistance (Figures 1G and 1H; Figures S1H, S2H, and S2I).

The normalization of body weight and adiposity in FGF21-overexpressing mice resulted from higher energy expenditure during both light and dark cycles (Figure 2E; Figure S4A). In this regard, gene set enrichment analysis (GSEA) of adipose tissues from 21-month-old male mice revealed over-representation of oxidative phosphorylation (OXPHOS) and other key mitochondrial-related metabolic processes, not only in iWAT but also in eWAT of AAV-FGF21-treated mice (Figures S3B and S3C). Cluster mapping of the GSEA leading-edge genes revealed enrichment in these depots of most enzymes involved in pyruvate metabolism and tricarboxylic acid (TCA) cycle and of the majority of components of all electron transport chain (ETC) complexes (Figures 2F and 2G; Figures S3D–S3F). AAV-FGF21-treated female mice also showed upregulation of key markers of these metabolic pathways (Figures S4B–S4D). The TCA cycle is the central metabolic hub of

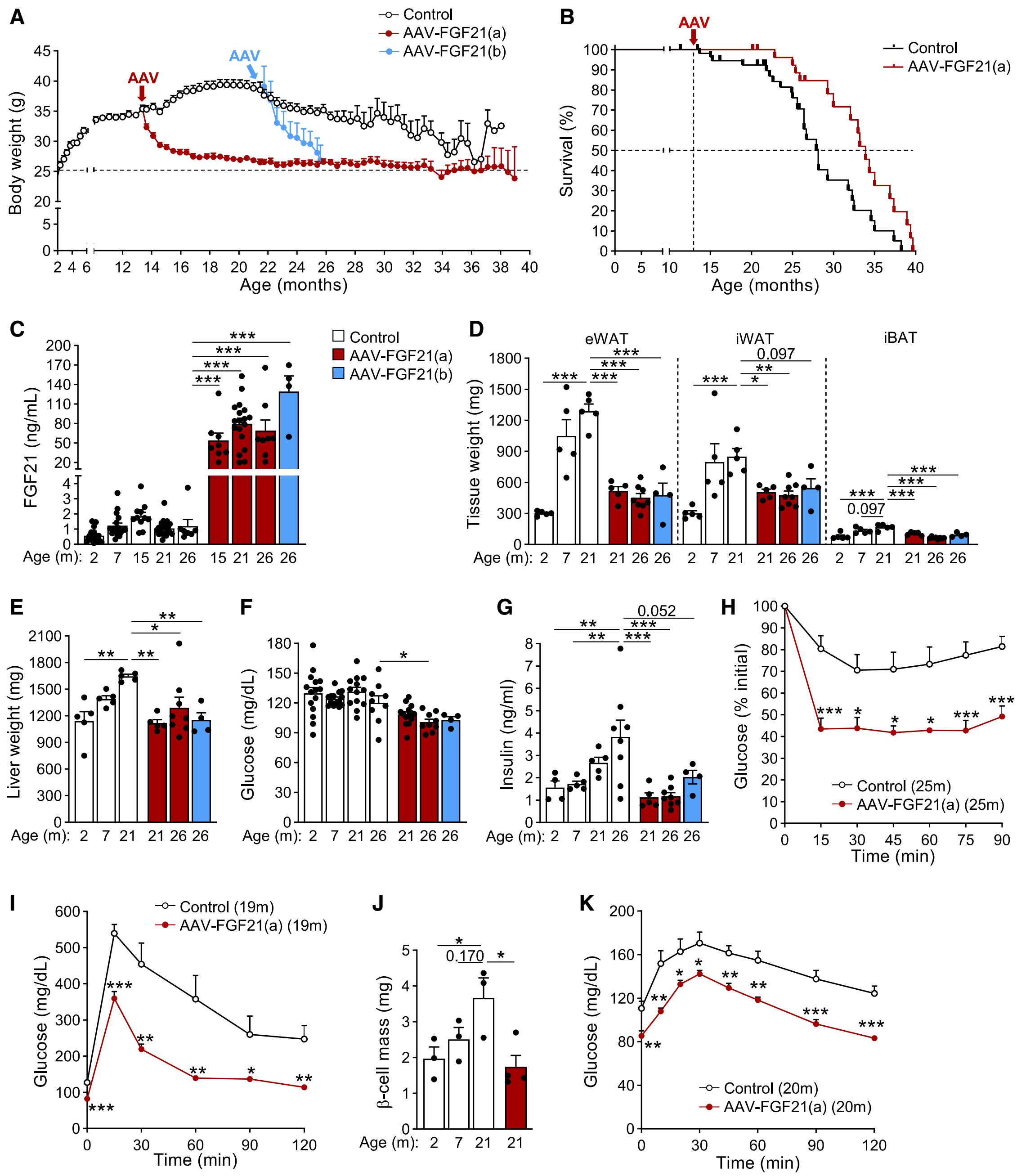

Figure 1. Treatment with AAV-FGF21 extends lifespan, counteracts body weight gain, and improves metabolism

(A) Body weight follow-up in mice treated with either AAV-Null (control, $n = 61$) or AAV-FGF21 vectors at 13 (AAV-FGF21 [a], $n = 32$) or 22 (AAV-FGF21 [b], $n = 4$) months of age. (B) Kaplan-Meier survival analysis ($n = 32–61$/group). (C) Serum FGF21 levels ($n = 4–24$/group). (D) Weight of the epididymal (eWAT) and inguinal (iWAT) white adipose tissue depots and the interscapular brown adipose tissue depot (iBAT) ($n = 4–8$/group). (E) Weight of the liver ($n = 4–8$/group). (F and G) Fed blood glucose (F, $n = 4–15$/group) and insulin (G, $n = 4–8$/group) levels. (H) Insulin sensitivity was determined after an intraperitoneal injection of insulin (0.375 units/kg body weight) at 25 months of age. Results were calculated as the percentage of initial blood glucose levels ($n = 3–6$/group). (I) Glucose tolerance test was determined after an intraperitoneal injection of glucose (2 g/kg body weight) at 19 months of age ($n = 7$/group). (J) Quantification of β cell mass ($n = 3–4$/group). (K) Hepatic gluconeogenesis was assessed by measurement of blood glucose excursion after an intraperitoneal injection of pyruvate (1 g/kg body weight) at 20 months of age ($n = 6$/group). Data are expressed as the mean ± SEM. $^{*}p < 0.05$, $^{**}p < 0.01$, $^{***}p < 0.001$ by one-way ANOVA with Dunnet's multiple comparison test (C–G and J) or Student's two-tailed *t* test (H, I, and K). m, months of age. Data were obtained in male mice.

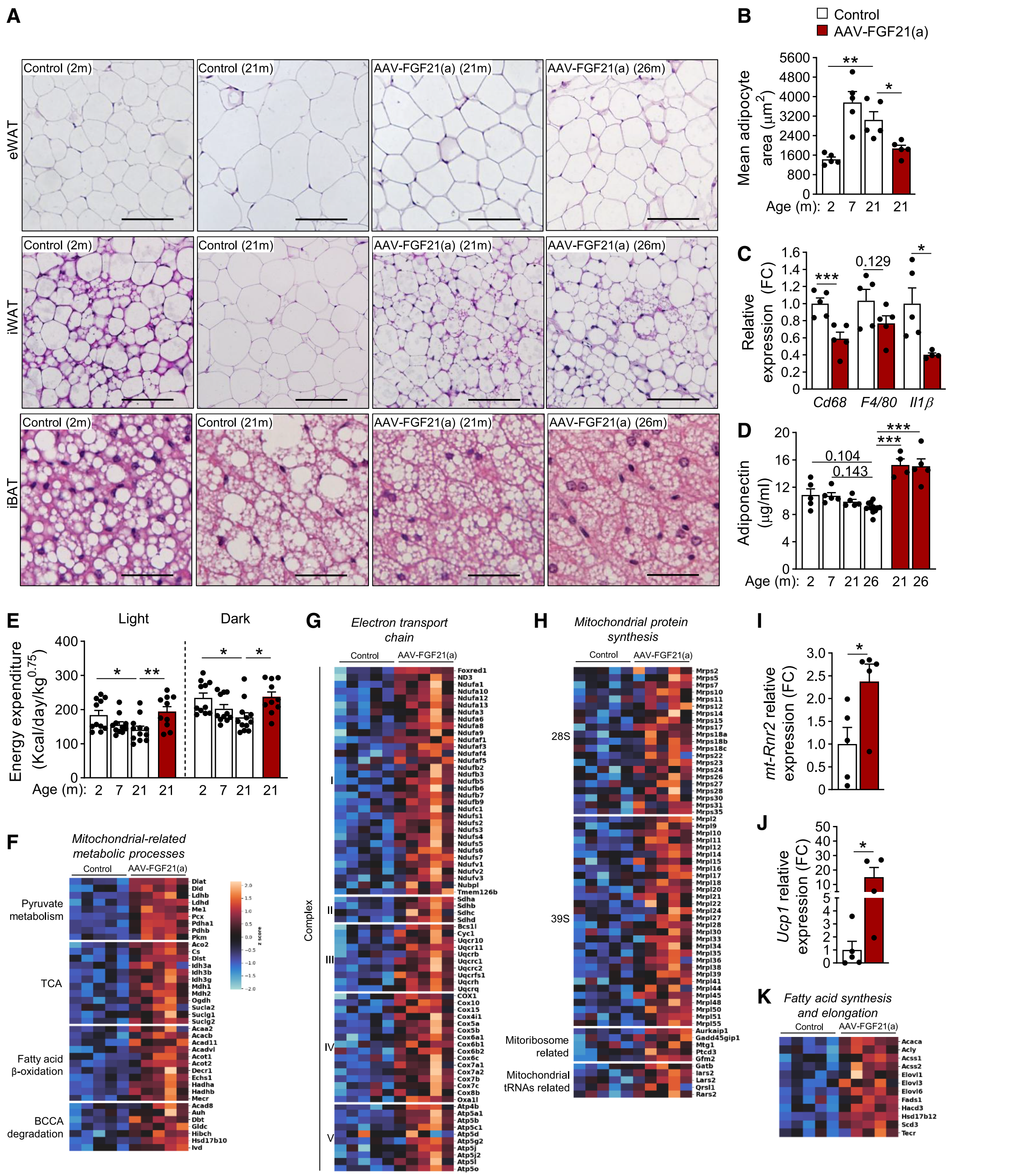

Figure 2. Treatment with AAV-FGF21 decreases lipid deposition in adipose tissue and increases energy expenditure

Male mice were treated with either AAV-Null (control) or AAV-FGF21 vectors at 13 months of age (AAV-FGF21 (a)). (A) Representative images of the hematoxylin–eosin staining of eWAT, iWAT, and iBAT sections. Scale bars: 100 μm (eWAT, iWAT) and 50 μm (iBAT). (B) Morphometric analysis of the adipocyte area in eWAT (n = 5/group). (C) Quantification by real-time quantitative PCR of the expression of the inflammatory markers *Cd68*, *F4/80*, and *Il1β* in eWAT at 21 months of age (n = 5/group). (D) Circulating levels of adiponectin (n = 5/group). (E) Energy expenditure was measured with an indirect open circuit calorimeter. Data were taken during the light and dark cycles (n = 10–12/group). (F–H) The heatmaps depict the normalized expression patterns of leading-edge genes involved in mitochondrial-related metabolic processes (F), electron transport chain components (G), and mitochondrial protein synthesis (H) in iWAT at 21 months of age. (I and J) Quantification by real-time quantitative PCR of the expression of the mitochondrial gene *mt-Rnr2* encoding the 16S ribosomal RNA (I) and the thermogenic marker *Ucp1* (J) in iWAT of 21-month-old mice (n = 4–5/group). (K) The heatmap

(legend continued on next page)

the cell producing NADH and $FADH_2$ to drive ETC and generate ATP through OXPHOS. Genes involved in fatty acid β-oxidation, which supplies substrates for both TCA cycle and ETC, were also enriched in iWAT (Figure 2F). Degradation of branched chain amino acids (BCAA; leucine, isoleucine, and valine) was also activated, likely to maintain intermediates for the TCA cycle (Figure 2F; Figures S3D and S3F). Upregulation of TCA cycle, OXPHOS, and amino acid metabolism was also observed in WAT of mice subjected to 17 lifespan-extending interventions.[23]

A high number of genes involved in translation of mtDNA-encoded polypeptides, which are essential structural components of ETC complexes I, III, IV, and V, were also enriched in iWAT (Figure 2H; Figure S4E). These included structural components of the small and large mitoribosomal subunits (28S and 39S: *Mrps* and *Mrpl* genes), genes involved in assembly, stabilization, and recycling of mitoribosomes (*Ptcd3Mtg1*, *Aurkaip1*, *Gadd45gip1*, *Gfm2*), as well as enzymes mediating synthesis and modification of mitochondrial tRNAs (*Iars2*, *Lars2*, *Rars2*, *Gatb*, *Qrsl1*) (Figure 2H; Figure S4E). Mitochondrial protein synthesis is essential for adipose tissue function, and its impairment contributes to metabolic dysregulation during aging.[24,25] In agreement with the globally enhanced expression of mitochondrial proteins, AAV-FGF21-treated mice exhibited increased mtDNA content in WAT (Figure 2I; Figure S3G).

The lack of enrichment for all these pathways in BAT (Figure S3H) likely stemmed from the tissue's pre-existing activation, a result of housing the mice at sub-thermoneutrality (22°C). Nevertheless, decreased lipid deposition in BAT of old AAV-FGF21-treated mice (Figure 2A) was indicative of enhancement of non-shivering thermogenesis (NST) in this depot. Accordingly, treatment with AAV-FGF21 vectors increased the expression of UCP1, the main molecular marker and effector of NST, in BAT (Figures S3I and S4F). Similarly, and in agreement with the induction of multilocular beige adipocytes in iWAT (Figure 2A), increased levels of UCP1 were observed in this tissue (Figure 2J; Figure S4G). Moreover, FGF21-overexpressing mice showed enrichment of genes related to fatty acid synthesis and elongation in iWAT (Figure 2K; Figure S4H). These processes sustain lipid reserves depleted by β-oxidation and optimize fatty acid composition for NST, which allows for a rapid substrate turnover to support high metabolic rates during NST.[26] Indeed, some of the upregulated genes (*Elovl3*, *Elovl6*) are key markers of NST (Figure 2K; Figure S4H). Collectively, these results indicated that NST was enhanced in iWAT and iBAT of aged mice treated with AAV-FGF21, contributing to the increased energy expenditure observed in these mice (Figure 2E; Figure S4A).

Development of fibrosis and dysregulated extracellular matrix (ECM) remodeling in WAT are also hallmarks of aging.[27] These processes are regulated by TGF-β and Wnt signaling pathways,[28] which were downregulated in iWAT of AAV-FGF21-treated mice (Figures 3A and 3B). Consequently, these animals also showed decreased expression of genes encoding ECM components (such as collagens) and of genes involved in ECM assembly and integrity (*Fbn1*, *Ctsk*, *Mmp11*) (Figure 3C). Adipose fibrosis impairs metabolic homeostasis, and ECM remodeling can alter mitochondrial homeostasis via TGF-β signaling.[29,30] Moreover, increased TGF-β signaling impairs browning of WAT.[31] Therefore, downregulation of the TGF-β and Wnt signaling pathways in AAV-FGF21-treated mice would not only prevent the development of fibrosis but also contribute to maintaining mitochondrial function and induction of iWAT browning.

Development of fibrosis in WAT has been reported to induce inflammation in this tissue, which also regulates adipose tissue aging.[32,33] Moreover, chronic low-grade inflammation, or "inflammaging," is a hallmark of aging and major contributor to age-related diseases.[34] In eWAT of AAV-FGF21-treated male mice, downregulation of the chemokine signaling pathway (Figure 3D) was consistent with the lack of low-grade inflammation in this depot (Figure 2C). Likewise, downregulation of immune response genes in WAT occurs across multiple health-span- and lifespan-extending interventions.[23] Moreover, the p53 signaling pathway was downregulated in eWAT of these mice (Figure 3E). Similarly, AAV-FGF21-treated female mice also showed reduced expression of markers of the chemokine and p53 signaling pathways in visceral WAT (Figures S4I and S4J). p53 downregulation in WAT has been reported to reduce senescence-associated changes, which decreases production of proinflammatory cytokines and macrophage infiltration, increases adiponectin levels, and improves insulin sensitivity.[35,36] In contrast, upregulation of p53, induced by aging or by other stimuli, causes an inflammatory response in WAT that leads to insulin resistance.[35] In addition, p53 is involved in energy metabolism. Downregulation of p53 in adipocytes can increase expression of mitochondrial enzymes involved in TCA cycle, ETC, and β-oxidation.[36–38] Similar observations were made in AAV-FGF21-treated mice (Figures S3D and S3E), suggesting that downregulation of p53 signaling also contributed to the improved energy metabolism observed in these mice. Additionally, activation of AMPK, a key mediator of FGF21 signaling,[39] was also observed in iWAT and iBAT of AAV-FGF21-treated mice (Figures 3F and 3G).Altogether, treatment with AAV-FGF21 vectors appeared to induce metabolic reprogramming in adipose tissue that enhanced mitochondrial function and mitigated aging-related signaling pathways, thereby improving cellular energetics and fitness.[39]

AAV-FGF21 treatment improves detoxifying metabolism in the liver

Although body and liver weight, as well as hepatic triglyceride content, increased with aging (Figures 1A and 1E; Figure 4A),

depicts the normalized expression patterns of leading-edge genes involved in fatty acid synthesis and elongation in iWAT at 21 months of age. In (B)–(E), data are expressed as the mean ± SEM. *$p < 0.05$, **$p < 0.01$, ***$p < 0.001$ by one-way ANOVA with Dunnet's multiple comparison test (B–E) or Student's two-tailed t test (I and J). BCAA, branched chain amino acids; eWAT, epididymal white adipose tissue; iBAT, interscapular brown adipose tissue; iWAT, inguinal white adipose tissue; FC, fold change; m, months of age; TCA, tricarboxylic acid cycle.

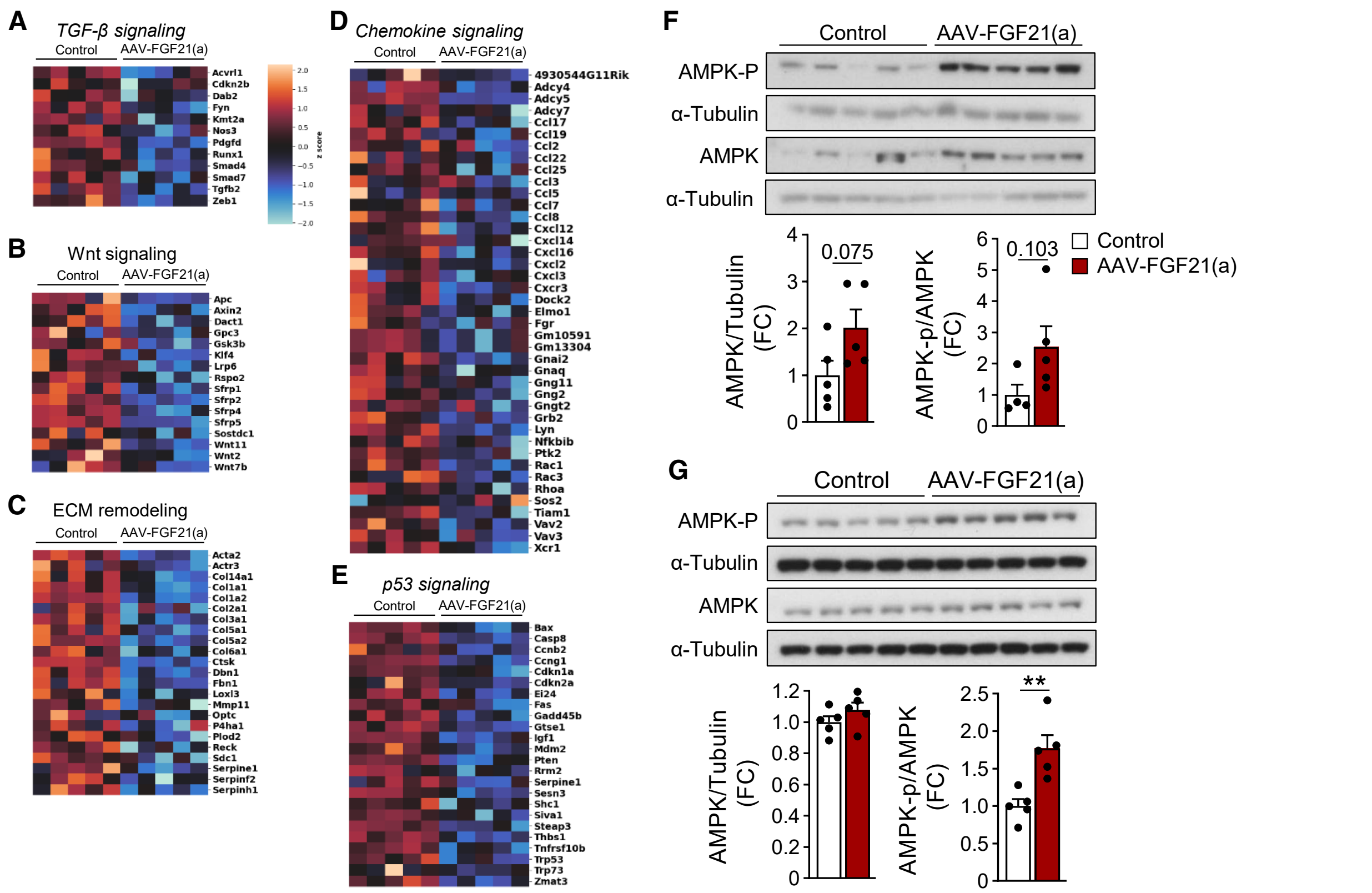

Figure 3. Effects of treatment with AAV-FGF21 in fibrosis- and inflammation-related processes in adipose tissue

Male mice were treated with either AAV-Null (control) or AAV-FGF21 vectors at 13 months of age (AAV-FGF21 (a)). (A–E) The heatmaps depict the normalized expression patterns of leading-edge genes involved in TGF-β (A) and Wnt (B) signaling, and ECM remodeling (C) in iWAT, and in chemokine (D) and p53 (E) signaling in eWAT at 21 months of age. (F and G) Western blot analysis of AMPK and AMPK-P content in iWAT (F) and iBAT (G) at 21 months of age. Representative immunoblots are shown (upper panels). Histograms depict the densitometric analyses of the immunoblots (lower panels). (n = 5/group). In (F) and (G), data are expressed as the mean ± SEM. $^{**}p < 0.01$ by Student's two-tailed t test. ECM, extracellular matrix; FC, fold change.

the levels reached were much lower than those observed during obesogenic states, such as high-fat diet feeding or genetic obesity.[16,17] In agreement with these findings, no steatosis was observed in liver sections of old control and AAV-FGF21-treated mice (Figure 4B). Consistent with the lack of hepatosteatosis, no striking differences in inflammation and collagen deposition were observed between groups at 21 months of age (Figures S5A and S5B). Nevertheless, at 26 months of age, histopathological analysis of the liver of control mice showed morphological alterations compatible with age-related amyloidosis, which were absent in AAV-FGF21-treated mice, indicating that this treatment prevented liver damage (Figures 4B and 4C).

The liver also plays a pivotal role in detoxification of drugs, environmental toxins, and endogenous molecules, which is crucial for maintaining cellular homeostasis. Hepatic detoxification occurs through two sequential phases: phase I introduces reactive or polar groups via oxidation, reduction, and hydrolysis, while phase II conjugates water-soluble molecules to these modified compounds, increasing their solubility for excretion. GSEA of the liver revealed that gene sets related to detoxification were overrepresented in AAV-FGF21-treated mice (Figure S5C). Moreover, cluster map analysis of the leading-edge genes demonstrated that genes of both phases were enriched in these animals (Figures 4D and 4E). A significant number of genes encoding cytochrome P450 (CYP) enzymes, principal catalysts of phase I metabolism, were upregulated in AAV-FGF21-treated mice (Figure 4D). Several of them belong to the CYP2A, CYP2B, CYP2C, CYP3A, and CYP4A families, which metabolize lipids, steroid hormones, bile acids, and arachidonic acid (Figure 4D).[40–43] Conversely, expression and activity of CYP enzymes and total CYP content decline during aging.[44] Flavin-containing monooxygenases (FMOs) are another class of key phase I enzymes involved in oxidative metabolism of xenobiotics and endogenous compounds. FGF21-overexpressing mice showed increased levels of FMO1–FMO4 (Figure 4D).

The balance between phase I and II is critical for effective detoxification. Treatment of aged mice with AAV-FGF21 vectors upregulated numerous phase II enzymes including glutathione S-transferases (GSTs), sulfotransferases (SULTs), and acyl-coenzyme A amino

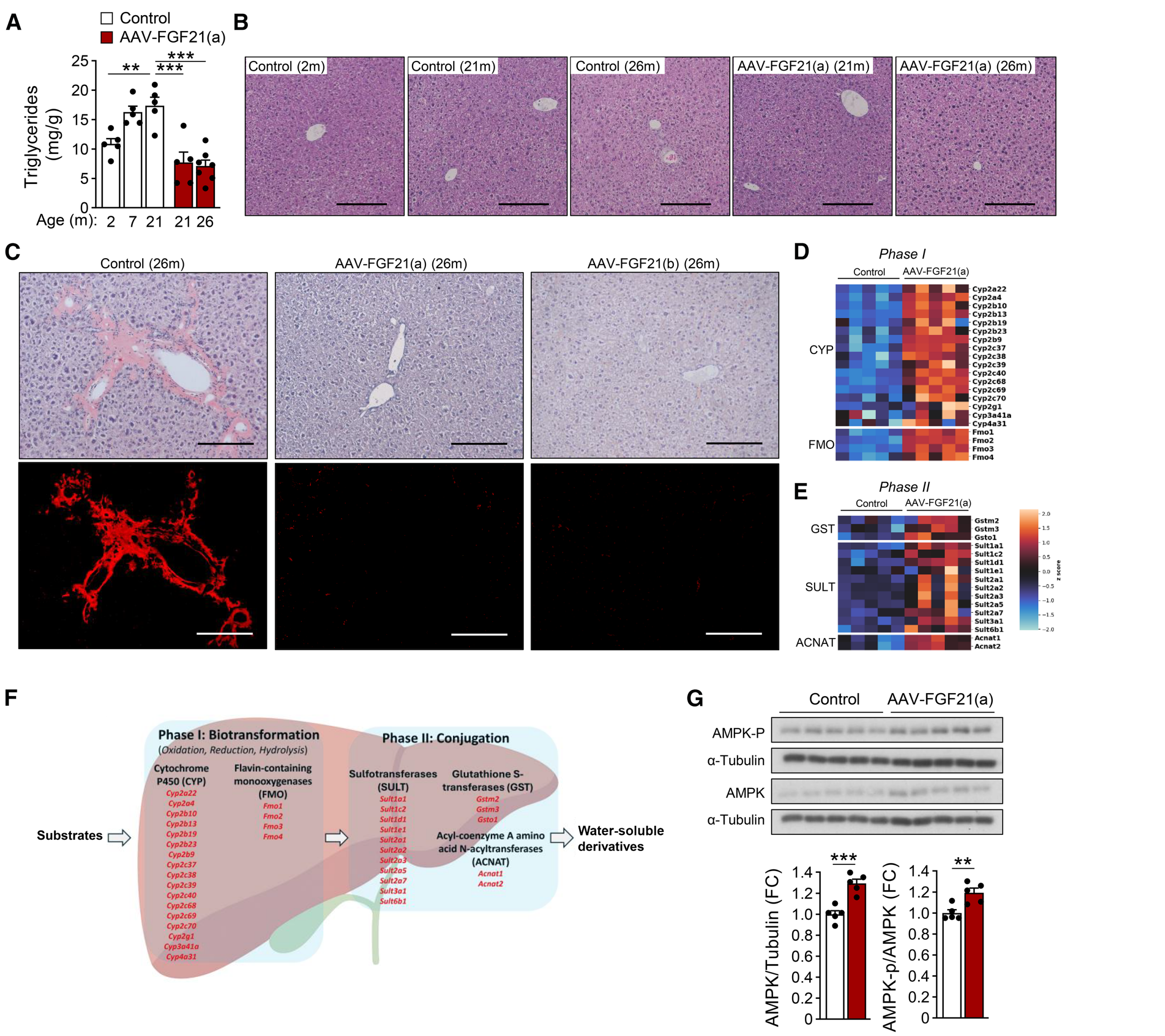

Figure 4. AAV-FGF21 gene therapy improves hepatic detoxification capacity

Male mice were treated with either AAV-Null (control) or AAV-FGF21 vectors at 13 (AAV-FGF21 (a)) or 22 (AAV-FGF21 (b)) months of age. (A) Liver triglyceride content (n = 5–7/group). (B and C) Representative images of hematoxylin-eosin (B) and Congo red (C; bright field, upper panel, and fluorescence signal, lower panel) staining of liver sections. Scale bars, 200 μm. (D and E) The heatmaps depict the normalized expression patterns of leading-edge genes of the phase I (D) and II (E) of liver detoxification at 21 months of age. (F) Scheme depicting the upregulated leading-edge genes involved in detoxifying metabolism in the liver of 21-month-old AAV-FGF21-treated mice (highlighted in red). (G) Western blot analysis of AMPK and AMPK-P content in the liver at 21 months of age. Representative immunoblots are shown. The histograms depict the densitometric analyses of the immunoblots (n = 5/group). In (A) and (G), data are expressed as the mean ± SEM. $^{**}p < 0.01$, $^{***}p < 0.001$ by one-way ANOVA with Dunnet's multiple comparison test (A) or Student's two-tailed t test (G). ACNAT, acyl-coenzyme A amino acid-N-acyltransferases; CYP, cytochrome P450; GST, glutathione S-transferases; FC, fold change; FMO, flavin-containing monooxygenases; m, months of age; SULT, sulfotransferases.

acid-N-acyltransferases (ACNATs) (Figure 4E). GST and SULT play critical roles in detoxification, oxidative stress regulation, and hormone metabolism with their expression declining during aging.[45,46] ACNAT1 and ACNAT2, which were increased in AAV-FGF21-treated mice (Figure 4E), catalyze the conjugation of fatty acids to the amino acid taurine, forming N-acyl taurines. These bioactive lipids modulate lipid metabolism and improve glucose homeostasis[47,48] and are also upregulated after caloric restriction.[49] Phase I and II leading-edge genes upregulated in the liver of aged mice treated with AAV-FGF21 are depicted in Figure 4F. Similar to the observations made in adipose tissue (Figures 3F and 3G), AAV-FGF21 treatment enhanced AMPK signaling in the liver (Figure 4G). Collectively, these results demonstrate that treatment with AAV-FGF21 counteracts hepatic age-related perturbations, thereby preserving liver detoxification capacity and function.

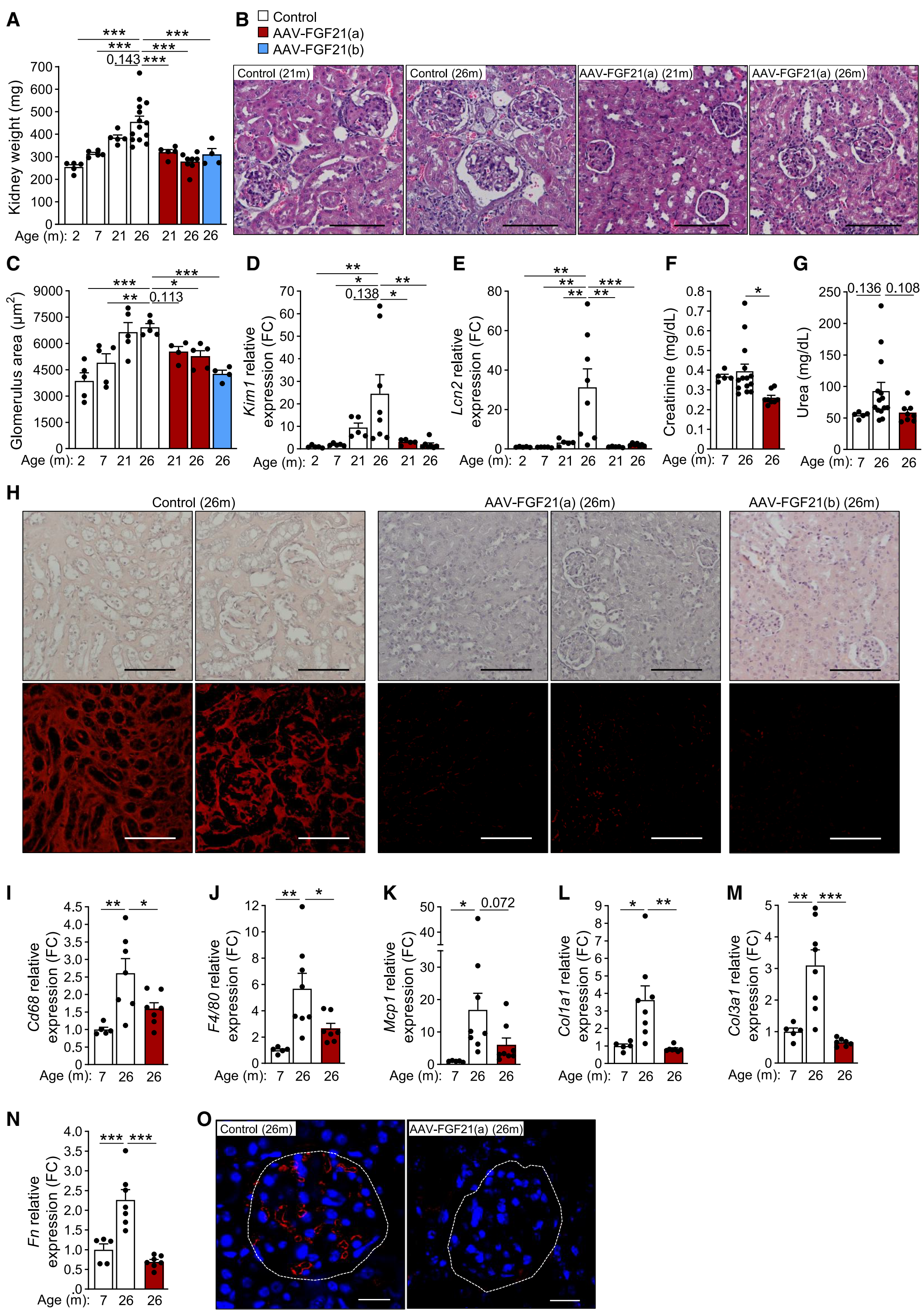

(legend on next page)

AAV-FGF21 treatment reverses age-related kidney disease

The aging process is associated with a time-dependent decline in kidney function and is recognized as a major contributor to the increased incidence of acute kidney injury and chronic kidney disease.[50] As animals aged, progressive increases, particularly pronounced at 26 months, were observed in key markers of impaired kidney function and age-related kidney disease, including kidney weight, glomerulus area, expression of Kidney Injury Molecule 1 (*Kim1)* and Lipocalin 2 (*Lcn2)*, and serum levels of creatinine and urea (Figures 5A–5G).[51,52] In addition, 26-month-old control mice showed age-related amyloidosis in the kidney (Figure 5H). Treatment of old and geriatric male and female mice with AAV-FGF21 vectors normalized these parameters (Figures 5A–5H; Figures S6A–S6D). Moreover, AAV-FGF21-treated mice reversed the age-associated overexpression of inflammatory and fibrotic markers in the kidney, including *Cd68, F4/80*, *Mcp1,* collagen type I alpha 1 chain (*Col1a1*), collagen type III alpha 1 chain (*Col3a1*), and fibronectin (*Fn*) (Figures 5I–5N; Figures S6E–S6J). Consistent with this, reduced fibronectin immunostaining was observed in kidney sections of AAV-FGF21-treated mice (Figure 5O). All these findings confirmed the absence of age-related kidney damage following FGF21 gene therapy.

AAV-FGF21 treatment prevents heart aging

The aging process causes progressive structural and functional deterioration of the heart in elderly individuals and represents the dominant risk factor for cardiovascular diseases development—the leading cause of death globally.[53] Histopathological analysis of hematoxylin-eosin-stained myocardium sections revealed pathological alterations in 26-month-old mice receiving AAV-Null vectors (Figure 6A). These sections stained positive for Congo red, confirming development of heart amyloidosis (Figure 6B), a condition closely associated with the aging process.[54] Moreover, Picrosirius red staining demonstrated abundant collagen fibers in the heart of 26-month-old control animals (Figures 6C and 6D). In contrast, FGF21-overexpressing mice showed no signs of amyloidosis or fibrosis (Figures 6B–6D). In agreement, decreased expression levels of *Col1a1*, *Col3a1*, and *Fn* were observed in male and female mice treated with AAV-FGF21 (Figure 6E; Figure S7A). Accordingly, transcriptomic evaluation of the heart in these mice revealed downregulation of TGF-β, the master regulator of fibrosis, and several genes associated with the Wnt signaling pathway and ECM remodeling (Figures 6F and 6G). Chronic activation of the TGF-β and Wnt signaling pathways in the aging heart promotes pathological remodeling (including hypertrophy and ECM deposition), reduces elasticity, and impairs contractility, ultimately leading to heart failure.[55]

Additionally, pathways related to enhanced protein synthesis capability were enriched in the hearts of AAV-FGF21-treated mice (Figure S7B). These mice showed upregulation of leading-edge genes encoding many structural proteins of both the small (40S) and large (60S) cytoplasmic ribosomal subunits (*Rps* and *Rpl* genes, respectively) and translation-associated factors (Figure 6H). The 40S subunit is critical for mRNA binding and translation initiation, whereas the 60S subunit is essential for peptide bond formation and elongation. Moreover, AAV-FGF21-treated mice showed overrepresentation of leading-edge genes involved in mitochondrial protein synthesis (*Mrps* and *Mrpl*) (Figure 6I). These findings suggested that AAV-FGF21 gene therapy not only enhanced cardiac proteostasis but also improved mitochondrial function through more efficient synthesis of ETC components encoded by mtDNA.

AAV1-FGF21 therapy improves muscular performance

Muscle function and physical performance progressively declined during aging (Figures 7A–7D).[56] However, old mice treated with AAV-FGF21 vectors exhibited enhanced motor coordination, balance, locomotor activity, endurance, and muscle strength, performing similarly to their young counterparts (Figures 7A–7D; Figures S8A and S8B). These results demonstrated preservation of muscular performance at advanced ages.

No alterations in myofiber morphology and integrity or muscle weight were observed between 21-month-old AAV-FGF21- and AAV-Null-treated mice (Figure 7E; Figures S9A–S9D). Nevertheless, at 26 months of age, Picrosirius red staining of quadriceps sections evidenced development of fibrosis in control mice, a hallmark of aging muscle, which was absent in FGF21-overexpressing mice (Figures 7F and 7G). In agreement, decreased expression levels of *Col1a1*, *Col3a1*, and *Fn* were also observed in mice treated with AAV-FGF21 (Figure 7H; Figure S8C). Similar to the transcriptomic changes observed in cardiac muscle, AAV-FGF21-treated mice also showed downregulation of leading-edge genes involved in TGF-β signaling and ECM remodeling in quadriceps (Figures 7I and 7J). This would prevent fibrosis development, thereby preserving muscle structure and preventing functional decline.[57,58]

Moreover, pathways involved in protein synthesis were overrepresented in quadriceps of AAV-FGF21-treated animals (Figure S9E). This gene therapy upregulated numerous *Rps* and *Rpl* genes as well as genes encoding translation-associated factors (Figure 7K). Expression of structural ribosomal constituents is also induced in skeletal muscle by other lifespan-promoting interventions.[23] Among

Figure 5. AAV-FGF21 treatment reverses age-related kidney disease

Male mice were treated with either AAV-Null (control) or AAV-FGF21 vectors at 13 (AAV-FGF21 (a)) or 22 (AAV-FGF21 (b)) months of age. (A) Kidney weight (n = 5–14/group). (B) Representative images of hematoxylin-eosin staining of kidney sections. Scale bars, 100 μm. (C) Glomerulus area (n = 4–5/group). (D and E) Quantification by real-time quantitative PCR of the expression of the kidney damage gene markers Kidney Injury Molecule 1 (*Kim1*) (d) and Lipocalin 2 (*Lnc2*) (e) (n = 5–14/group). (F and G) Serum creatinine (F) and urea (G) levels (n = 5–14/group). (H) Representative images of Congo red staining of kidney sections (bright field, upper panels; fluorescence signal, lower panels). Scale bars, 100 μm. (I and N) Quantification by real-time quantitative PCR of the expression of the inflammatory and fibrosis gene markers *Cd68* (I), *F4/80* (J), *Mcp1* (K), *Col1a1* (L), *Col3a1* (M), and fibronectin (*Fn*) (N) (n = 5–8/group). (O) Representative images of kidney sections immunostained against fibronectin. Scale bars, 20 μm. Data are expressed as the mean ± SEM. *$p < 0.05$, **$p < 0.01$, ***$p < 0.001$ by one-way ANOVA with Dunnet's multiple comparison test. FC, fold change; m, months of age.

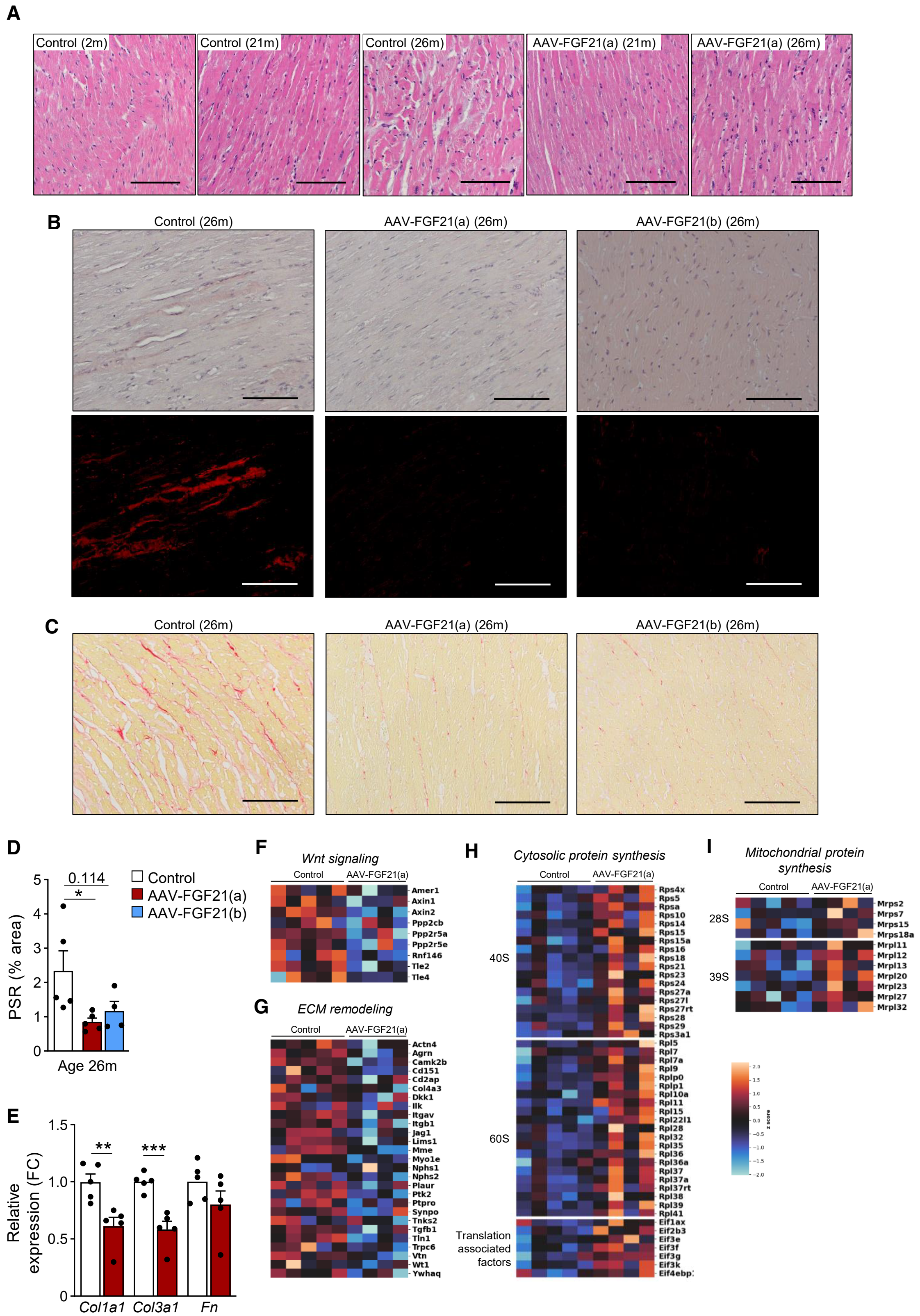

(legend on next page)

translation-associated factors, AAV-FGF21 treatment upregulated genes encoding the following: (1) critical ribosomal assembly and maturation factors, such as *Rps15*, *Rpl10A*, and *Fau*, a pivotal gene for 40S subunit maturation; (2) translation initiation regulators including *Eif4ebp1*, which controls cap-dependent translation, and *Eif3f* and *Eif3k*, which form the elF3 complex critical for ribosome assembly; and (3) mRNA processing and translational regulation factors like *Rnps1*, an RNA-binding protein involved in splicing and surveillance, and *Srp14* and *Srp72*, signal recognition particle components important for targeting secretory and membrane proteins (Figure 7K). Collectively, these AAV-FGF21-mediated changes suggested maintenance of protein synthesis capacity, whose decline is a hallmark of aged muscle contributing to reduced function.[59–61]

In addition to muscular performance, the effects of long-term increased FGF21 circulating levels on bone in aged mice were also assessed. At 26 months of age, mice treated at 13 months did not exhibit alterations in serum markers of bone formation, including procollagen type 1 N-terminal propeptide (PINP) and osteocalcin, nor in the bone resorption marker tartrate-resistant acid phosphatase 5b (TRAcP 5b), despite sustained elevation of circulating FGF21 for more than 1 year (Figures S9F–S9H). Moreover, circulating carboxy-terminal crosslinked telopeptide of type 1 collagen (CTX-1) was lower in these mice than in age-matched controls (Figure S9I), indicating reduced degradation of the type I collagen bone matrix. These data demonstrated that sustained elevated skeletal muscle-derived FGF21 circulating levels did not induce bone toxicity in aged mice. These findings are consistent with previous studies showing that long-term AAV-mediated increased FGF21 levels in obese, insulin-resistant mice did not induce bone alterations.[16,62] Moreover, non-human primates treated for 1 year with an FGF21 mimetic did not exhibit adverse effects on bone mineral density or bone structure.[63]

AAV-FGF21 treatment reverses age-associated cognitive decline

Impaired memory and learning are the most common consequences of the aging process in the central nervous system in the absence of neurodegenerative diseases.[64] By 27 months of age, male mice treated with FGF21-encoding vectors showed enhanced memory compared with age-matched controls, equivalent to that of 2-month-old animals in the novel object recognition test (Figures 8A and 8B). Moreover, evaluation of performance in the rotarod test during subsequent trials demonstrated that AAV-FGF21-treated mice exhibited improved motor learning ability to age-matched counterparts (Figure S10A). Treatment with AAV-FGF21 vectors also improved cognition in old female mice (Figures S10B and S10C).

Similar to observations in adipose tissue, skeletal muscle, and heart, GSEA of brain tissue from old mice treated with FGF21-encoding vectors revealed over-representation of processes related to mitochondrial energy metabolism (TCA cycle, OXPHOS, and ETC) as well as pathways associated with enhanced protein translation (Figure S10D). Specifically, AAV-FGF21-treated mice showed enrichment of leading-edge genes encoding key TCA cycle enzymes and components of the ETC complexes I–V (Figures 8C and 8D). The brain is a highly energy-demanding organ that relies on ATP production via the TCA cycle and OXPHOS, which is essential for synaptic function, neurotransmission, ion pump activity, and overall neuronal survival.[65,66] Moreover, mitochondrial function and energy metabolism decline significantly with age, and their impairment is critical for the development of neurodegenerative diseases such as Alzheimer's and Parkinson's diseases.[65,66] In 25-month-old AAV-FGF21-treated mice, measurement of key OXPHOS markers by qPCR further corroborated the transcriptomic findings and suggested efficient ATP production in cortex and hippocampus (Figure 8E), the main brain areas involved in cognitive function. Similarly, these mice also showed increased expression of key glycolysis-related genes in these regions (Figure 8F). The brain almost exclusively uses glucose as a substrate for energy metabolism, which is metabolized through glycolysis and TCA cycle.[65,66] Nevertheless, in agreement with previous reports,[67,68] treatment with AAV-FGF21 increased circulating levels of β-hydroxybutyrate (Figure S10E), which could also be utilized by the brain as an alternative energy substrate in addition to glucose.[69]

AAV-FGF21-treated mice also showed upregulation of *Rps* and *Rpl* genes, as well as genes encoding translation initiation (EIFs), elongation factors (EEFs and TSFM), and additional translation-related factors (SRP68, PABPCL1, FAU) (Figure 8G). Upregulation of these leading-edge genes may also support cognitive function by maintaining synaptic plasticity, which depends heavily on rapid, on-demand synthesis of new proteins at the synapse.[70,71] Mitochondrial perturbations and reduced ATP production also contribute to synaptic dysfunction and degeneration, which strongly correlate with cognitive deficits and memory loss.[66,72,73] Notably, by 25 months of age, animals treated with AAV-FGF21 showed increased expression of genes encoding key synaptic proteins in cortex and hippocampus, including synaptophysin, and subunits of the alpha-amino-3-hydroxy-5-methyl-4-isoxazole proprionic acid (AMPA)-type and N-methyl-d-aspartate (NMDA)-type ionotropic glutamate receptors

Figure 6. AAV-FGF21 treatment prevents heart aging

Male mice were treated with either AAV-Null (control) or AAV-FGF21 vectors at 13 (AAV-FGF21 (a)) or 22 (AAV-FGF21 (b)) months of age. (A–C) Representative images of hematoxylin-eosin (A), Congo red (B; bright field, upper panels; fluorescence signal, lower panels), and PicroSirius red (C) staining of heart sections. Scale bars, 100 μm. (D) Histomorphometric quantitative assessment of heart fibrosis (PSR) (n = 4–5/group). (E) Quantification by RT-qPCR of the expression of the fibrosis gene markers *Col1a1*, *Col3a1*, and *Fn* (n = 5/group). (F–I) The heatmaps depict the normalized expression patterns of leading-edge genes involved in Wnt signaling (F), ECM remodeling (G), cytosolic (H), and mitochondrial protein synthesis (I) in heart at 21 months of age. Data in (D) and (E) are expressed as the mean ± SEM. *$p < 0.05$, **$p < 0.01$ and ***$p < 0.001$ by one-way ANOVA with Dunnet's multiple comparison test (D) or Student's two-tailed t test (E).

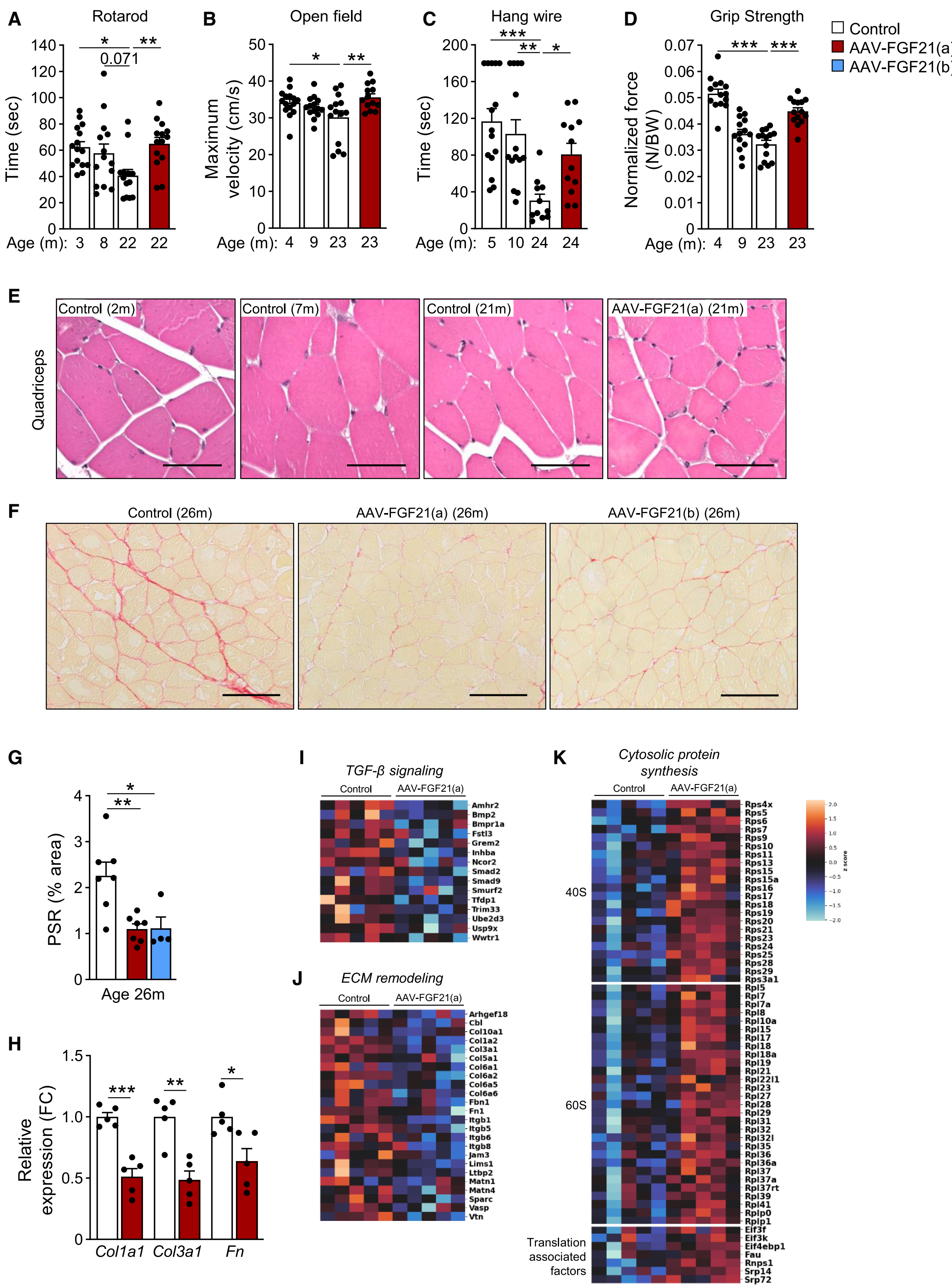

(legend on next page)

(Figure 8H). Overall, the coordinated upregulation of mitochondrial, metabolic, and protein synthesis-related genes would maintain the brain's energy-producing capacity and support brain health during aging, thereby preventing cognitive decline.

In aged mice treated with AAV-FGF21 vectors, FGF21 levels in the brain increased (Figure S10F), although they remained markedly lower than the circulating concentrations (Figure 1C). Since native FGF21 can cross the blood-brain barrier,[74] these direct central effects may contribute to the therapeutic outcomes. In addition, efficacy may reflect AAV-FGF21-driven amelioration of obesity, tissue inflammation, and insulin resistance, which are metabolic perturbations that negatively impact brain function.[75–77]

DISCUSSION

This study demonstrated that a single administration of the muscle-directed AAV-FGF21 gene therapy led to sustained, long-term increases in circulating levels of FGF21 in aged mice, making the factor continuously available to all organs, including the brain.[74] The AAV-FGF21 treatment drove four interconnected adaptive programs that counteracted fundamental aging mechanisms. First, enhanced mitochondrial function was evident across most tissues, characterized by upregulation of TCA cycle enzymes, ETC components, and mitochondrial ribosomal proteins. This coordinated enhancement would address mitochondrial dysfunction, a primary hallmark of aging,[1,78] and provide energetic capacity essential for tissue function. Energy-dependent organs including adipose tissue, brain, heart, and skeletal muscle showed particularly robust mitochondrial improvements correlating with functional preservation. Second, restored proteostasis was evident through widespread upregulation of cytoplasmic and mitochondrial ribosomal proteins and translation factors. Maintenance of protein synthesis capacity, which declines progressively with age,[79] would have contributed to preserved muscle function, cardiac health, and synaptic plasticity underlying cognitive improvements. Third, suppression of pro-fibrotic and proinflammatory pathways, such as TGF-β, Wnt, and p53 signaling, would have prevented pathological tissue remodeling, fibrosis, and chronic inflammation characterizing aging tissues.[34] Prevention of amyloidosis in liver, kidney, and heart demonstrated protection against age-related protein aggregation. Fourth, the coordinated upregulation of phase I and phase II detoxification enzymes likely maintained the capacity of the liver to efficiently metabolize and eliminate toxic compounds and metabolic by-products, thereby attenuating cellular stress and supporting the preservation of systemic metabolic homeostasis. Enhanced hepatic detoxification capacity is a molecular signature of multiple lifespan-extending interventions.[23] Together, these four processes link molecular changes to functional health span improvements and extension of midlife survival.

Widespread expression of FGF21 receptors (FGFR1–4) and the co-receptor β-Klotho have been reported throughout tissues in the body[80] and likely enabled the observed multi-organ gerotherapeutic benefits. In this regard, all tissues examined showed detectable β-Klotho expression, with the highest levels in the liver and adipose depots, that were further induced in these tissues following AAV-FGF21 treatment. In tissues such as skeletal muscle, heart, kidney, and brain where bulk β-Klotho expression was comparatively low, global measurements may underestimate functionally relevant expression in specific cellular subpopulations.[81–84] Nevertheless, the widespread phenotypic improvements observed after muscle-derived increased circulating FGF21 levels may also be mediated by indirect, systemic mechanisms. In particular, increased circulating adiponectin, a well-established downstream effector of FGF21,[68,85] has been reported to exert protective effects in kidney, heart, and skeletal muscle through anti-inflammatory, anti-fibrotic, anti-oxidative, and metabolic pathways,[68,86–89] thus likely contributing to the FGF21-derived therapeutic effects. Moreover, the improved systemic metabolic homeostasis induced by AAV-FGF21 gene therapy would reduce lipotoxic and oxidative stress in kidney, heart, and skeletal muscle, even in the absence of robust local β-Klotho signaling.

Additionally, AAV-FGF21 gene therapy in previous studies was able to counteract already established MASH, T2D, and obesity, major chronic age-associated metabolic diseases, and prevented development of hepatocellular carcinomas and obesity-associated cognitive decline and anxiety in obese mice,[16,17] supporting its clinical translation to treat MASH (https://kriyatherapeutics.com/pipeline/). Unlike these earlier studies, which focused on genetically induced or diet-induced relevant mouse models of these severe metabolic diseases,[16,17] the current study used naturally aged mice, which had already developed spontaneous multi-organ age-related deterioration. This shift from a pathological to a physiological context allowed to demonstrate, for the first time, that a single i.m. administration of AAV-FGF21 vectors not only improved metabolic parameters but extended health span and lifespan, producing robust and systemic geroprotective effects. In addition, integrated whole-organ transcriptomic and histopathological analyses revealed

Figure 7. AAV1-FGF21 therapy improves muscular performance in old male mice

Male mice were treated with either AAV-Null (control) or AAV-FGF21 vectors at 13 (AAV-FGF21 (a)) or 22 (AAV-FGF21 (b)) months of age. (A) Time before falling during the rotarod test (n = 14–15/group). (B) Maximum velocity was measured in the open field test (n = 13–15/group). (C) Hang time during the hang wire test (n = 11–15/group). (D) Forelimb grip strength (n = 14/group). (E) Representative images of hematoxylin-eosin staining of quadriceps sections. Scale bars, 100 μm. (F) Representative images of PicroSirius red staining of quadriceps sections. Scale bars, 100 μm. (G) Histomorphometric quantitative assessment of fibrosis in quadriceps (PSR) (n = 4–7/group). (H) Quantification by real-time quantitative PCR of the expression of the fibrosis gene markers *Col1a1*, *Col3a1*, and *Fn* (n = 5/group). (I–K) The heatmaps depict the normalized expression patterns of leading-edge genes involved in TGF-β signaling (I), ECM remodeling (J), and cytosolic protein synthesis (K) in quadriceps at 21 months of age. In (A)–(D) and (F), data are expressed as the mean ± SEM. *$p < 0.05$, **$p < 0.01$, ***$p < 0.001$ by one-way ANOVA with Dunnet's multiple comparison test (G) or Student's two-tailed t test (H). BW, body weight; m, months of age.

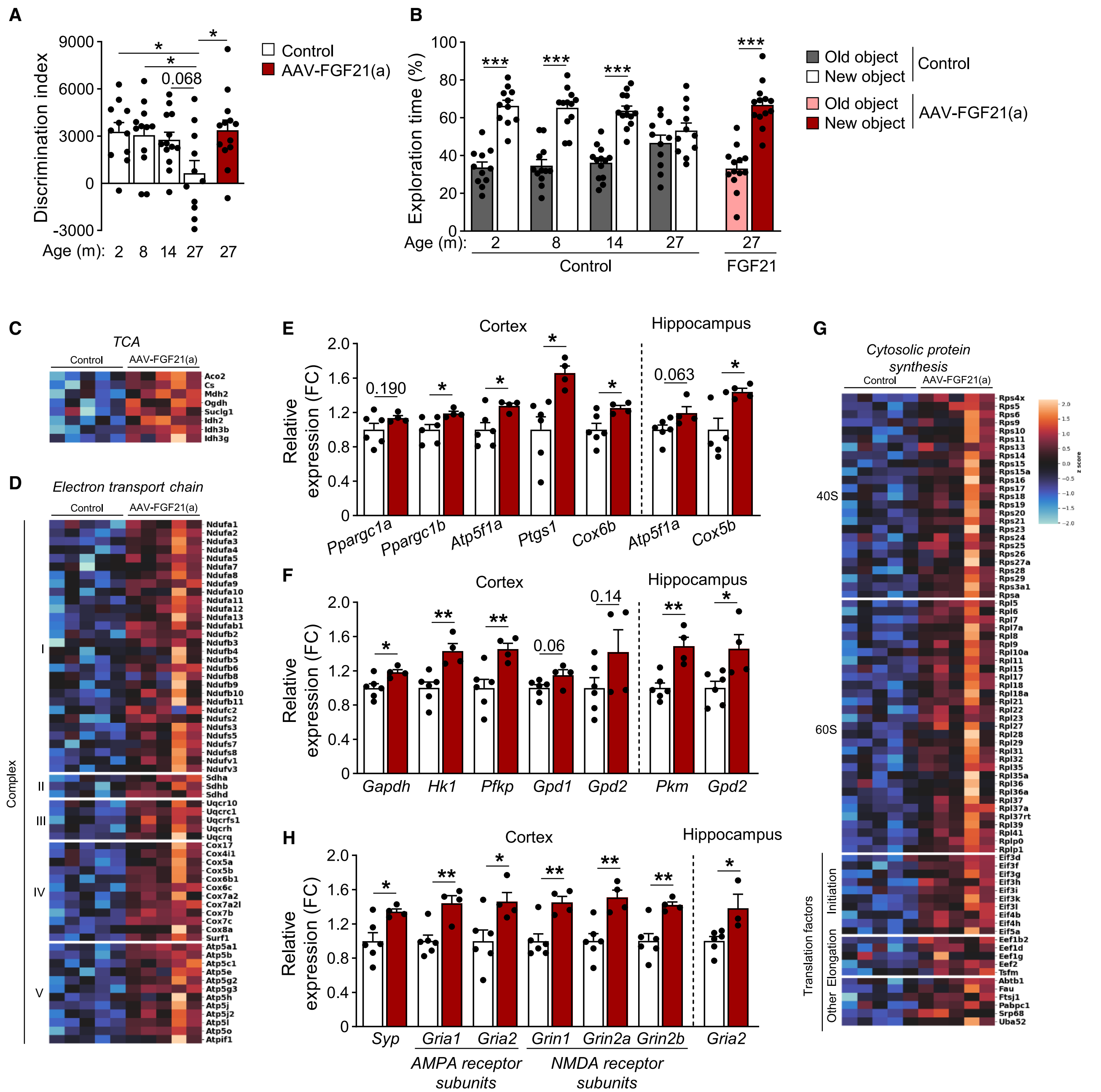

Figure 8. AAV-FGF21 treatment reverses age-associated cognitive decline in old male mice

Male mice were treated with either AAV-Null (control) or AAV-FGF21 vectors at 13 months of age (AAV-FGF21 (a)). (A and B) Discrimination index (A) and exploration time (B) during novel object recognition (NOR) test (n = 11–13/group). (C and D) The heatmaps depict the normalized expression patterns of leading-edge genes encoding TCA enzymes (C) and ETC components (D) in brain at 21 months of age. (E and F) Quantification by real-time quantitative PCR of the expression of key gene markers of OXPHOS (E) and glycolysis (F) in cortex and hippocampus of 25-month-old mice (n = 4–5/group). (G) The heatmap depicts the normalized expression patterns of leading-edge genes involved in cytosolic protein synthesis in brain at 21 months of age. (H) Quantification by real-time quantitative PCR of the expression of genes encoding key synaptic proteins in cortex and hippocampus of 25-month-old mice (n = 4–5/group). In (A), (B), (E), (F), and (H), data are expressed as the mean ± SEM. *$p < 0.05$, **$p < 0.01$, ***$p < 0.001$ by one-way ANOVA with Dunnet's multiple comparison test (A) or Student's two-tailed t test (B, E, F, and H). TCA, tricarboxylic acid cycle; *Ppargc1a*, peroxisome proliferator-activated receptor gamma coactivator 1 alpha; *Ppargc1b*, peroxisome proliferator-activated receptor gamma coactivator 1 beta; *Atp5f1a*, ATP synthase F1 subunit alpha; *Ptgs1*, prostaglandin-endoperoxide synthase 1; *Cox6b*, cytochrome *c* oxidase subunit 6b; *Cox5b*, cytochrome *c* oxidase subunit 5b; *Gapdh*, glycerldehyde-3-phosphate

(legend continued on next page)

tissue-specific adaptive responses that collectively preserved cellular fitness during natural aging. Whereas previous publications positioned AAV-FGF21 primarily as a promising treatment for metabolic disorders,[16,17] the present work established its gerotherapeutic potential capable of modulating core biological hallmarks of aging, thereby enhancing its translational relevance for an expanding aging population.

While other previous studies have delineated the biological role of FGF21 in aging, their translational relevance remains intrinsically constrained as they used transgenic mouse models but not developed FGF21-based therapeutic strategies.[90–94] In this regard, hepatocyte-specific FGF21 transgenic mice exhibited improved metabolic parameters and markedly extended lifespan, but in this model, FGF21 overexpression was already activated during embryogenesis,[67,90,95] thereby precluding inference on the efficacy of therapeutic intervention in adult organisms. Moreover, these transgenic mice were dwarf and exhibited low bone mass, and as they aged, they failed to maintain reduced adiposity and showed no improvement in glucose tolerance or phosphorylated AMPK levels in liver and adipose tissue.[90,96] More recently, a doxycycline-inducible adipocyte-FGF21 transgenic mouse model was developed to avoid overexpression from embryogenesis, and it demonstrated that FGF21 promotes longevity and confers metabolic benefits.[93] Nevertheless, in contrast to the present work, in this study using transgenic mice, FGF21 overexpression was induced in young ages (10–12 weeks of age), rather than in old animals. Additionally, the chronic high-fat-diet regimen limited the experimental context to diet-induced metabolic dysfunction instead of natural aging physiology.[93] Moreover, doxycycline-inducible systems cannot be used in gene therapy approaches to drive expression of therapeutic genes because of their immunogenicity.[97]

On the other hand, protein restriction confirmed that FGF21 upregulation is required for metabolic and lifespan improvements, but the intervention was initiated in young male mice.[97] When 12-month-old male mice were subjected to an 8-week methionine-restricted diet, they also exhibited increased serum FGF21 levels and a reversal of age-associated changes in body weight, adiposity, physical activity, and glucose tolerance.[98] However, due to the short duration of the intervention, lifespan outcomes were not assessed, nor were potential therapeutic benefits on cognitive function evaluated in these animals.[98] Moreover, the deleterious consequences of long-term restriction of the essential amino acid methionine remain unexplored.

In addition, compared with the robust gerotherapeutic effects observed in the present study, previous AAV-FGF21-based strategies have produced partial and limited outcomes. Systemic administration of liver-targeted AAV8-FGF21 vectors to 18-month-old male mice reduced body weight in a 5-month follow-up, but this was the only aging-related phenotype reported.[62] In the same study, AAV8-FGF21 vectors were tested against surgically induced renal fibrosis and cardiac failure in mice.[62] However, these experimentally induced models do not reflect the spontaneous age-associated organ decline of physiologically aged mice. Likewise, adipose-directed Rec2-AAV-FGF21 gene therapy in 17-month-old female mice modestly reduced body weight and slightly increased energy expenditure, without improving glucose tolerance, grip strength, rotarod performance, circulating adiponectin, liver weight, hepatic triglycerides, or β-Klotho expression in liver or adipose tissue.[99] This limited efficacy was consistent with the minimal serum FGF21 elevation achieved,[99] in contrast to the robust and sustained systemic FGF21 levels induced by i.m. AAV-FGF21 administration in the present study.

The absence of long-term adverse events in the present work, and in previous studies in mice and dogs,[16,17] highlighted the high safety profile of the AAV-FGF21 gene therapy. In this regard, a large body of work in large animals and human patients has demonstrated that i.m. delivery of AAV vectors to genetically engineer skeletal muscle is safe and well tolerated.[17,18,100–108] Moreover, durability of transgene expression in large animals and humans has been documented for 8 and 10 years, respectively, demonstrating that the capacity of AAV-engineered muscles to produce and secrete therapeutic proteins persisted as treated animal and human subjects grew older.[18,106] Notably, in type 1 diabetic Beagle dogs treated i.m. with AAV vectors encoding insulin and glucokinase, using the same delivery route, AAV1 serotype, and CMV promoter as in the present study, transgene expression and therapeutic efficacy were sustained for a duration that essentially spanned the animals' lifetimes.[18,100] This program is currently in the IND-enabling phase of development (https://kriyatherapeutics.com/pipeline/).

While AAV vectors are generally regarded as safe, systemic administration of high doses of these vectors may elicit adverse outcomes in clinical studies in patients, including hepatotoxicity, complement activation, severe immune-mediated responses, and, in certain contexts, life-threatening responses.[109–112] Nevertheless, AAV vectors administered via the i.m. route exhibit restricted systemic biodistribution, especially the AAV1 serotype, which largely remains in transduced muscle fibers, as it lacks the capacity to cross endothelial barriers.[16–18,100] Furthermore, in the AAV-FGF21 approach, genetic engineering of a limited subset of skeletal muscles in hindlimbs is sufficient to establish a therapeutic pump for FGF21 secretion into bloodstream, thereby reducing vector dose requirements and, consequently, the risk of detrimental host responses.[16,17] Additionally, i.m. administration of AAV vectors for gene delivery is not substantially hindered by pre-existing neutralizing antibodies against the AAV capsid,[107,108,113,114] a critical consideration given the

dehydrogenase; *Hk1*, hexokinase 1; *Pfkp*, platelet isoform of phosphofructokinase; *Gpd1*, glycerol-3-phosphate dehydrogenase 1; *Gpd2*, glycerol-3-phosphate dehydrogenase 2; *Pkm*, pyruvate kinase M; *Syp*, synaptophysin; *Gria1* and *Gria2*, GluR1 and GluR2 subunits of the alpha-amino-3-hydroxy-5-methyl-4-isoxazole proprionic acid (AMPA)-type ionotropic glutamate receptor; *Grin1*, *Grin2a*, and *Grin2b*, NR1, N2A, and N2B subunits of the N-methyl-d-aspartate (NMDA)-type ionotropic glutamate receptor; FC, fold change; m, months of age.

high prevalence of anti-AAV immunity in the general population, which can otherwise limit the clinical applicability of AAV-based therapies.[115]

The geroprotective effects demonstrated in the current study, alongside a robust safety profile, support translation of AAV-FGF21 gene therapy as a therapeutic strategy to promote health span extension. Nonetheless, additional studies in large animals are required prior to human application. Prolonging health span encompasses profound individual benefits, including enhanced functional capacity, preserved autonomy, and reduced multimorbidity, and it alleviates societal burdens by lowering healthcare utilization and costs and productivity loss. This is the priority of the United Nations Decade of Healthy Aging (2021–2030), whose implementation is led by the World Health Organization, focused on advancing interventions designed to link increasing longevity with extended well-being (https://www.decadeofhealthyageing.org/). In this context, our AAV-FGF21-based gene therapy holds potential to be transformative for health span extension by targeting whole-body hallmarks of aging.

MATERIALS AND METHODS

Animals

Male and female C57BL/6J mice were kept in a specific pathogen-free facility (SER-CBATEG, UAB) and maintained under a light-dark cycle of 12 h at 22°C. Mice were fed *ad libitum* with a standard diet (2018S Teklad Global Diets, Envigo). For tissue sampling, mice were anesthetized with inhalational anesthetic isoflurane (IsoFlo, Abbott Laboratories) and decapitated. Tissues of interest were excised and kept at −80°C or in formalin until analysis. Animal care and experimental procedures were approved by the Ethics Committee in Animal and Human Experimentation of the Universitat Autònoma de Barcelona (UAB).

Recombinant AAV vectors

An AAV expression cassette was obtained by cloning, between the ITRs of AAV2, the murine codon-optimized FGF21 coding sequence (*moFGF21*) under the control of the cytomegalovirus (CMV) promoter (Table S1). A non-coding cassette carrying the CMV promoter but no transgene was used to produce AAV-Null vectors. Single-stranded AAV1 vectors were produced by triple transfection in HEK293 cells and purified using a double CsCl gradient-based purification protocol that renders vector preps of high purity with negligible amounts of empty capsids.[116] Viral genome titers were determined by PicoGreen (Quant-iT PicoGreen dsDNA assay kit, Invitrogen), using phage lambda DNA to generate the standard curve. The same batch of AAV-FGF21 vectors was used for all the mouse experimental work.

Intramuscular administration of AAV vectors

Mice were anesthetized with an intraperitoneal injection of ketamine (100 mg/kg) and xylazine (10 mg/kg). Hindlimbs were shaved, and AAV vectors were i.m. administered in a volume of 180 μL divided into six injection sites distributed between quadriceps, gastrocnemius, and tibialis cranialis of each hindlimb (one site per skeletal muscle).

Insulin tolerance test

Insulin (Humulin Regular; Eli Lilly) was injected intraperitoneally to fed mice at a dose of 0.375 or 0.75 IU/kg body weight. Glycemia was measured in tail vein blood samples at the indicated time points.

Glucose and pyruvate tolerance tests

Awake mice were fasted overnight (16 h) and administered with an intraperitoneal injection of glucose (2 g/kg body weight) or pyruvate (1 g/kg body weight). Glycemia was measured in tail vein blood samples at the indicated time points.

Indirect calorimetry

An indirect open circuit calorimeter (Oxylet, Panlab) was used to monitor O_2 consumption and CO_2 production. Mice were individualized and acclimated to the metabolic chambers for 24 h, and data were collected in each cage for 5 min, every 45 min, for 24 h. Data were taken during the light and dark cycles and adjusted by body weight.

Rotarod test

Mice were placed on a rotating rod (Panlab), spinning at 4 rpm. Lane width was 50 mm; rod diameter was 30 mm. Once stabilized, mice were subjected to an incrementally increasing speed from 4 to 40 rpm in 5 min. The first day of the experiment was used to train the animals in the use of the device. Each animal underwent 3 trials. The latency to fall from the rod was recorded. Then, animals underwent 1 day resting, and the third day, mice took 3 more trials on the rod. The average of 3 trials was analyzed.

Open field test

The open field test was performed between 9:00 a.m. and 1:00 p.m. Mice were placed in a corner of a square arena surrounded by high white walls (45 × 45 × 40 cm), and motor and exploratory activities were evaluated during the first 6 min using a video tracking system (SMART Junior, Panlab).

Hang wire test

The hang wire test was conducted using a 55-cm-wide, 2-mm-thick metallic wire, which was secured to two vertical stands. The wire was maintained 35 cm above a layer of bedding material to prevent injury to the animal when it falls down. Mice, handled by the tail, were allowed to grasp the middle of the wire with their forelimbs. The time until mice fell down was measured. Mice that reached the limit suspension time of 180 s, regardless of the trial number, were allowed to stop the experiment, while the others were directly retested for a maximum of three trials (a 30-s recovery period was used between trials). The average of 3 trials was analyzed.

Grip strength test

A grip strength test meter (Panlab) was used to assess forelimb grip strength. The grip strength meter was positioned horizontally, and

mice were held by the tail and lowered toward the apparatus. Animals were allowed to grasp the metal bar with their forepaws and were then pulled backward in the horizontal plane. The force applied to the bar just before it lost grip was recorded as the peak tension. The average of 3 trials was analyzed.

Novel object recognition test

The novel object recognition tests were conducted in the open field box.[17] Open field test was used to acclimatize the mice to the box. The next day, to conduct the first trial, two identical objects (A and B) were placed in the upper right and upper left quadrants of the box, and then mice were placed backward to both objects. After 10 min of exploration, mice were removed from the box and allowed for 10 min break. In the second trial, one of the identical objects (A and B) was replaced with object C (new object). Mice were then put back into the box for a further 10 min of exploration. The amount of time animals spent exploring the novel object was recorded and evaluated using a video tracking system (SMART Junior; Panlab). Mice with exploration times for both objects shorter than 5 s, either during the first or the second trial, were excluded from analysis, as it cannot be confirmed that they spent enough time exploring to learn/discriminate the objects. The evaluation of novel object recognition test memory was expressed as a percentage of the discrimination ratio calculated according to the following formula: Discrimination ratio (%) = $(N - F)/(N + F) \times 100\%$, where N represents the time spent in exploring the new object, and F represents the time spent in exploring the same object.

Serum parameters and metabolite assays

Hepatic triglyceride content was determined by chloroform:methanol (2:1 vol/vol) extraction of total lipids.[17] Triglycerides and β-hydroxybutyrate were quantified spectrophotometrically using an enzymatic assay (Horiba-ABX). Glycemia was determined using a Glucometer Elite (Bayer). The Mouse/Rat FGF-21 ELISA kit (MF2100, R&D Systems) was used to determine FGF21 levels in serum and brain. Brain samples (cerebellum) were homogenized with T-PER buffer (Thermo Fisher Scientific) using a sonicator (VC130PB, Sonics & Materials). Buffer was supplemented with protease inhibitors (Complete, Roche) and phosphatase inhibitors (orthovanadate). Once homogenized, samples were centrifuged at 10,000 $\times$ *g* during 10 min at 4°C, and supernatant was kept at −80°C until analysis. Serum insulin, adiponectin, osteocalcin, CTX-1, TRAcP 5b, and PINP levels were determined using the Rat Insulin ELISA kit (90010, Crystal Chem), the Mouse Adiponectin ELISA kit (80569, Crystal Chem), the ELISA Kit for Osteocalcin (SEA471Mu, Cloud-Clone Corp.), the RatLaps (CTX-I) EIA kit (AC-06F1, Immunodiagnostics Systems Limited), the MouseTRAP (TRAcP 5b) ELISA kit (SB-TR103, Immuno Diagnostics Systems Limited), and the Rat/Mouse PINP EIA kit (AC-33F1, Immuno Diagnostics Systems Limited), respectively. Serum creatinine and urea levels were measured by spectrophotometry at the Servei de Bioquímica Clínica Veterinària (Veterinary School, UAB).

Immunohistochemistry

Tissues were fixed for 12–24 h in 10% formalin, embedded in paraffin, and sectioned. Sections were incubated overnight at 4°C with guinea pig anti-insulin (1:100; I-8510; Sigma-Aldrich) or rat anti-fibronectin (1:100; F3648; Sigma). Biotinylated rabbit anti-guinea pig coupled to peroxidase (1:300, P0141, Dako) or Donkey anti-rabbit 568 (a10042, Invitrogen) were used as secondary antibodies. The ABC peroxidase kit (Pierce) was used for immunodetection, and sections were counterstained in Mayer's hematoxylin. Hoechst (B2261; Sigma-Aldrich) was used for nuclear counterstaining of fluorescent specimens. PicroSirius red staining and Congo red staining were used to evaluate fibrosis and amyloidosis, respectively. Morphometric analysis of β cell mass, adipocyte size, and glomerulus area were performed as previously described.[17,117] Tissue fibrosis was assessed by the percentage of the PSR-positive area quantified in 10 randomly selected $\times$100 fields per tissue section.[17] Images were obtained with a Nikon Eclipse 90i microscopy (Nikon).

Western blot analysis

For total protein extracts, tissues were homogenized in protein lysis buffer and centrifuged at 15,000 $\times$ *g* during 15 min at 4°C. Proteins were separated by 12% SDS-PAGE and analyzed by immunoblotting with Anti-AMPK (2532, Cell Signaling), Antiphospho-AMPK-Thr172 (2531, Cell Signaling), and Anti-α-tubulin (ab4074, Abcam). Detection was performed using ECL Plus detection reagent (Amersham Biosciences).

RNA analysis

Total RNA was obtained from different tissues using isolation reagent (Tripure, Roche, for liver, kidney, heart, skeletal muscle, and brain, or QIAzol, Qiagen, for adipose depots) and an RNeasy Minikit (Qiagen) and treated with DNAseI (Qiagen). 1 μg of RNA was reverse-transcribed using the Transcriptor First Strand cDNA Synthesis kit (Roche). Real-time quantitative PCR was performed in a Lightcycler (Roche) using the Lightcycler 480 SyBr Green I Master Mix (Roche) and the following primers:

moFGF21: 5′CCTAACCAGGACGCCACAAG3′, 5′GTTCCACCATGCTCAGAGGG3′; *Cd68*: 5′GGGGCTCTTGGGAACTACAC3′, 5′CAAGCCCTCTTTAAGCCCCA3′;

F4/80: 5′CTTTGGATGGGCTTCCAGTC3′, 5′GCAAGGAGGACAGAGTTTATC3′;

Il1β: 5′ 5′TGTAATGAAAGACGGCACACC3′, 5′TCTTCTTTGGGTATTGCTTGG3′; *Mcp1*: 5′AACTGCATCTGCCCTAAGGTC3′, 5′AAGTGCTTGAGGTGGTTGTG3′;

Ucp1: 5′GGATTGGCCTCTACGACTCAG3′, 5′TGTAGGCTGCCCAATGAACA3′;

mt-Rnr2: 5′-CCGCAAGGGAAAGATGAAAGAC-3′, 5′ TCGTTTGGTTTCGGGGTTTC3′; *mt-Nd1*: 5′ CTAGCAGAAACAAACCGGG

C3′, 5′ CCGGCTGCGTATTCTACGTT3′; *Kim1*: 5′AAGCCGCAGAAAAACCTAC3′, 5′ AACCACGCTTAGAGATGCTG3′;

Lcn2: 5′CAGAAGGCAGCTTTACGATG3′; 5′CCTGGAGCTTGGAACAAATG3′;

Col1a1: 5′GACTGGAAGAGCGGAGAGTA3′, 5′CCTTGATGGCGTCCAGGTT3′; *Col3a1*: 5′AGGATCTGTCCTTTGCGATG3′, 5′TCTCCAAATGGGATCTCTGG3′;

Fn: 5′AAGATTGGCGACAAGTGGAG3′, 5′TAGGTTTGCAGGTCCATTCC3′;

Ppargc1a: 5′TTTGGCCGACGACACGACTTTTC3′, 5′TTGTGTTGGGCGAGAGAAAG3′;

Ppargc1b: 5′AGAAGCGCTTTGAGGTGTTC3′, 5′GGTGATAAAACCGTGCTTCTGG3′; *Atp5f1a*: 5′GGCCATTTTGTGCCAGTCG3′, 5′CTGACCAATCGCGACGTAGA3′;

Ptgs1: 5′TATCCAACTCATCCCTTGAC3′, 5′GAGTAGCGTCGTGGTATTCC3′;

Cox6b: 5′AGTCCCTCTGTCCCGTGTC3′, 5′ATATGCTGAGGTCCCCCTTT3′;

Cox5b: 5′CTCGTCAGCCTCAGCCAGT3′, 5′TAGCAGCGAATGGAACAGAC3′;

Gapdh: 5′CCTTCCGTGTTCCTACCC3′, 5′CAACCTGGTCCTCAGTGTAG3′;

Hk1: 5′GACCGTTCCTCAGACACCTG3′, 5′CTCTGTGAGAGGGGGTCGTA3′;

Pfkp: 5′TGTGTCTGAAGGAGCAATCG3′, 5′GGCCAAAATCCTGTCAAATG3′;

Gpd1: 5′AGACACCCAACTTTCGCATC3′, 5′TATTCTTCAAGGCCCCACAG3′;

Gpd2: 5′TTGCCTTGGGAGAAGATGAC3′, 5′AGTTCCGCACTTCATTCAGG3′;

Pkm: 5′GCTTTGCATCTGATCCCATT3′, 5′AGTCCAGCCACAGGATGTTC3′;

Syp: 5′ACATGGACGTGGTGAATCAG3′, 5′AAGATGGCAAAGACCCAGTG3′;

Gria1: 5′CCATGCTGGTTGCCTTAATC3′, 5′CCGTATGGCTTCATTGATGG3′;

Gria2: 5′AACGGCGTGTAATCCTTGAC3′, 5′TTTCAGCAGGTCTCCATCAG3′;

Grin1: 5′TGACTACCCGAATGTCCATC3′, 5′TTGTAGACGCGCATCATCTC3′;

Grin2a: 5′TGTGAAGAAGTGCTGCAAGG3′, 5′CGCCTATCATTCCATTCCAC3′;

Grin2b: 5′TTGGTGAGGTGGTCATGAAG3′, 5′TGCGTGATACCATGACACTG3′;

Klb: 5′ACGAGGGCTGTTTTATGTTGG3′, 5′TTCAAAGGGAAGCCGTTGTC3′;

Pcx: 5′TTCCTGCAGGGCTACATTGG3′, 5′GGCAGGTCCTTTAGCACCTT3′;

Pdha1: 5′TGTGTGATGGTCAGGAAGCC3′, 5′CCGAGTGAAGGTGAAGCCAT3′;

Cs: 5′CCCACAGAGGAACAGGTGTC3′, 5′ACCACATGAGAAGGCAGAGC3′;

Ndufa1: 5′CAACGGGGGCAAGGAAAAAC3′, 5′CCAGGCCCTTGGACACATAG3′;

Sdha: 5′GTTGCAGCACAGGGAGGTAT3′, 5′TCGGAGCCTTTCACAGTGTC3′;

Uqcrq: 5′TTCAGCAAAGGCATCCCCAA3′, 5′TAGACCACTACAAACGGCGG3′;

Cox5a: 5′TTGCGAGCATGTAGACGGTT3′, 5′TGAGGTCCTGCTTTGTCCTT3′;

Mrpl12: 5′GGCAAAGCCTGTGGACAAAG3′, 5′CTACCAGCTTCTTGGCCTGG3′;

Mrps35: 5′CACCACAGACAGATGCCCTT3′, 5′CATCTGCAGCAGGGTCTGAA3′;

Elovl3: 5′AAACCGTGTGCTTTGCCATC3′; 5′CCGTGTCTCCCAGTTCAACA3′;

Cdkn1a: 5′CAGACCAGCCTGACAGATTTCT3′, 5′CTGACCCACAGCAGAAGAGG3′;

Cdkn2a: 5′TCTTGGTGAAGTTCGTGCGA3′, 5′TGCCCATCATCATCACCTGG3′;

Rplp0: 5′TCCCACCTTGTCTCCAGTCT3′, 5′ACTGGTCTAGGACCCGAGAAG3′; *nqHK2:* 5′GCCAGCCTCTCCTGATTTTAGTGT3′, 5′GGGAACACAAAAGACCTCTTCTGG3′.

Data were normalized to *Rplp0* expression, except for mtDNA-encoded genes, which were normalized to *nqHK2* expression.

Transcriptomic analysis

Microarray plate processing was carried out at the High Technology Unit (UAT) of the Vall d'Hebron Research Institut (VHIR) (Barcelona, Spain). The Agilent 2100 Bioanalyzer was used to assess sample RNA integrity prior to the microarray protocol. The Affymetrix GeneTitan microarray platform and the Genechip Mouse Clariom S array plate were used in this study. This array plate analyzes gene expression patterns on a whole-genome scale on a single plate with probes covering many exons on the target genomes and, thus, permitts an accurate expression summarization at gene level. The starting material was 200 ng of total RNA for each sample, obtained as previously described. The quality of isolated RNA was first measured by Bioanalyzer Assay (Agilent). Briefly, sense single-stranded DNA (ssDNA) suitable for labeling was generated from total RNA with the GeneChip WT Plus Reagent Kit from Affymetrix (Thermo Fisher-Affymetrix) according to the manufacturer's instructions. Sense ssDNA was fragmented, labeled, and hybridized to the arrays with the GeneChip WT Terminal Labeling and Hybridization Kit from the same manufacturer. Arrays plates were scanned to obtain.cel files, and quality control was performed with the Affymetrix Expression Control Software.

GSEA was performed to interpret transcriptomic data from a microarray analysis. This method uses gene sets, which are groups of genes that share common features based on existing biological knowledge from databases like the Molecular Signatures Database (MSigDB) (https://www.gsea-msigdb.org/gsea/msigdb). The analysis was conducted by first ordering genes in a ranked list based on their differential expression between two classes: AAV-FGF21 and AAV-Null. Next, an enrichment score was calculated for each gene set to determine if its members were overrepresented at the top or bottom of the ranked list. This score reflects the degree of enrichment for a particular gene set. Gene lists from several sources were used to gain biological insights, including Biological Process, KEGG Pathway, Reactome pathways, and Wikipathways. After GSEA, a leading-edge analysis was performed to identify the specific genes that contributed most to the enrichment of each category. These leading-edge genes and their corresponding categories were then clustered in a cluster map (Table S2). This process identified overlapping genes in enriched categories, which helped summarize the most relevant biological information and supported manual curation based on bibliographic research. GSEA and leading-edge identification were performed using the easy Visualization and Inference Toolbox for Transcriptome Analysis (eVITTA), and further data analysis and visualization were conducted in Python programming language.

Vector genome copy number

Tissue samples were digested overnight at 56°C in 300 μL of Tissue Lysis Solution supplemented with Proteinase K (0.2 mg/mL). Total DNA was isolated from supernatants using the MasterPure DNA purification kit. DNA was resuspended in Milli-Q water and quantified using a SynergyTM HTX Multi-ModeMicroplate reader. Vector genome copy number in 40 ng of total DNA was determined by quantitative PCR and primers and probe specific for the murine codon-optimized FGF21 coding sequence (*moFGF21*): forward primer: 5′GAGCCTGCTGGAACTGAAG3′; reverse primer: 5′TCAGGATCGAAGTGAGGAGAG3′; probe: 5′/56-FAM/CTTCACGCC/ZEN/CAGGATCTGGATCAC/3IABkFQ/3′. A reference standard curve was built from serial dilutions of the linearized AAV plasmid bearing the moFGF21 sequence spiked into 20 ng/μL of non-transduced mouse genomic DNA.

Statistical analysis and data processing

Sample size determination was based on previous experience with similar studies. Randomization was performed using the excel function Roundup() or by GraphPad QuickCalcs to allocate mice in each group. In addition, we tested that the mean body weight and the mean glycemia were statistically not different for each experimental group prior to assignment to treatment groups. Furthermore, each experimental group was caged separately to avoid any caging effects. All tests (ITT, GTT, open field, etc.) were performed by investigators blinded to the treatment. All results are expressed as mean ± SEM. The GraphPad Prism 7 software was used for statistical analyses. Data were analyzed by one-way analysis of variance (ANOVA) with Dunnett's multiple comparison test, except for those parameters involving comparison of only two experimental groups, in which case Student's two-tailed t test was used. The Kaplan-Meier method was used to analyze survival, and the log rank test was used for comparisons. Differences were considered significant when $p < 0.05$.

DATA AND CODE AVAILABILITY

Array data have been deposited in the ArrayExpress database (https://www.ebi.ac.uk/arrayexpress/; accession code E-MTAB-15771). Other data supporting the findings of this study are available within the paper and its supplemental information.

ACKNOWLEDGMENTS

The authors thank Lorena Noya, Marta Moya, Jennifer Barrero, and Lidia Hernandez for technical assistance. This work was supported by the grants PID2020-113864RB-100 funded by MICIU/AEI/https://doi.org/10.13039/501100011033 and PID2023-148027OB-I00 funded by MICIU/AEI/https://doi.org/10.13039/501100011033 and by "ERDF/EU", by Generalitat de Catalunya (2021 SGR 01012 with support from "Departament d'Universitats, Recerca i Societat de la Informació" and ICREA Academia Award to F.B.) and by CIBER-Consorcio Centro de Investigación Biomédica en Red-(CB07/08/0037), Instituto de Salud Carlos III, Ministerio de Ciencia e Innovación, Spain. The graphical abstract was generated with BioRender (https://www.biorender.com/).

AUTHOR CONTRIBUTIONS

F.B. and V.J. conceived the project, designed and supervised experiments, and wrote the manuscript. V.J., V.S., and C.J. generated reagents and performed experiments. M.G. performed experiments and transcriptomic analyses. X.L. generated reagents. E.C., S. Muñoz, L.V., I.G., M.L.J., C.R., S. Marcó, M.M., A.R., I.E., J.R., and T.F. performed experiments. V.J., V.S., M.G., and F.B. analyzed the data, contributed to discussion, and reviewed/edited the manuscript.

DECLARATION OF INTERESTS

V.J., V.S., M.G., C.J., E.C., I.E., I.G., A.R., and F.B. are co-inventors on patent applications for the use of AAV vectors for the treatment of metabolic diseases. F.B. is a member of the Scientific Advisory Board of Kriya Therapeutics.

SUPPLEMENTAL INFORMATION

Supplemental information can be found online at https://doi.org/10.1016/j.ymthe.2026.05.025.

REFERENCES

1. López-Otín, C., Blasco, M.A., Partridge, L., Serrano, M., and Kroemer, G. (2013). The Hallmarks of Aging. Cell *153*, 1194–1217. https://doi.org/10.1016/j.cell.2013.05.039.
2. Catic, A. (2018). Cellular Metabolism and Aging. Prog. Mol. Biol. Transl. Sci. *115*, 85–107. https://doi.org/10.1016/bs.pmbts.2017.12.003.
3. Palmer, A.K., and Jensen, M.D. (2022). Metabolic changes in aging humans: current evidence and therapeutic strategies. J. Clin. Invest. *132*, e158451. https://doi.org/10.1172/JCI158451.
4. Pontzer, H., Yamada, Y., Sagayama, H., Ainslie, P.N., Andersen, L.F., Anderson, L. J., Arab, L., Baddou, I., Bedu-Addo, K., Blaak, E.E., et al. (2021). Daily energy expenditure through the human life course. Science *373*, 808–812. https://doi.org/10.1126/science.abe5017.
5. Kuk, J.L., Saunders, T.J., Davidson, L.E., and Ross, R. (2009). Age-related changes in total and regional fat distribution. Ageing Res. Rev. *8*, 339–348. https://doi.org/10.1016/j.arr.2009.06.001.
6. Fu, S., Yao, Y., Lv, F., Zhang, F., Zhao, Y., and Luan, F. (2018). Associations of immunological factors with metabolic syndrome and its characteristic elements in Chinese centenarians. J. Transl. Med. *16*, 315. https://doi.org/10.1186/s12967-018-1691-4.
7. Arai, Y., Kamide, K., and Hirose, N. (2019). Adipokines and Aging: Findings From Centenarians and the Very Old. Front. Endocrinol. *10*, 142. https://doi.org/10.3389/fendo.2019.00142.
8. Sanchez-Roman, I., Ferrando, B., Holst, C.M., Mengel-From, J., Rasmussen, S.H., Thinggaard, M., Bohr, V.A., Christensen, K., and Stevnsner, T. (2022). Molecular markers of DNA repair and brain metabolism correlate with cognition in centenarians. Geroscience *44*, 103–125. https://doi.org/10.1007/s11357-021-00502-2.
9. Frankowska, N., Bryl, E., Fulop, T., and Witkowski, J.M. (2023). Longevity, Centenarians and Modified Cellular Proteodynamics. Int. J. Mol. Sci. *24*, 2888. https://doi.org/10.3390/ijms24032888.
10. Green, C.L., Lamming, D.W., and Fontana, L. (2022). Molecular mechanisms of dietary restriction promoting health and longevity. Nat. Rev. Mol. Cel Biol. *23*, 56–73. https://doi.org/10.1038/s41580-021-00411-4.
11. Herzig, S., and Shaw, R.J. (2018). AMPK: guardian of metabolism and mitochondrial homeostasis. Nat. Rev. Mol. Cel Biol. *19*, 121–135. https://doi.org/10.1038/nrm.2017.95.
12. Guan, Y., and Yan, Z. (2022). Molecular Mechanisms of Exercise and Healthspan. Cells *11*, 872. https://doi.org/10.3390/cells11050872.
13. Kulkarni, A.S., Gubbi, S., and Barzilai, N. (2020). Benefits of Metformin in Attenuating the Hallmarks of Aging. Cell Metab. *32*, 15–30. https://doi.org/10.1016/j.cmet.2020.04.001.
14. Campbell, J.M., Bellman, S.M., Stephenson, M.D., and Lisy, K. (2017). Metformin reduces all-cause mortality and diseases of ageing independent of its effect on diabetes control: A systematic review and meta-analysis. Ageing Res. Rev. *40*, 31–44. https://doi.org/10.1016/j.arr.2017.08.003.
15. Flippo, K.H., and Potthoff, M.J. (2021). Metabolic Messengers: FGF21. Nat. Metab. *3*, 309–317. https://doi.org/10.1038/s42255-021-00354-2.
16. Jimenez, V., Jambrina, C., Casana, E., Sacristan, V., Muñoz, S., Darriba, S., Rodó, J., Mallol, C., Garcia, M., León, X., et al. (2018). FGF21 gene therapy as treatment for obesity and insulin resistance. EMBO Mol. Med. *10*. https://doi.org/10.15252/emmm.201708791.
17. Jimenez, V., Sacristan, V., Jambrina, C., Jaen, M.L., Casana, E., Muñoz, S., Marcó, S., Molas, M., Garcia, M., Grass, I., et al. (2024). Reversion of metabolic dysfunction-associated steatohepatitis by skeletal muscle-directed FGF21 gene therapy. Mol. Ther. *32*, 4285–4302. https://doi.org/10.1016/j.ymthe.2024.10.023.
18. Jaén, M.L., Vilà, L., Elias, I., Jimenez, V., Rodó, J., Maggioni, L., Ruiz-de Gopegui, R., Garcia, M., Muñoz, S., Callejas, D., et al. (2017). Long-Term Efficacy and Safety of Insulin and Glucokinase Gene Therapy for Diabetes: 8-Year Follow-Up in Dogs. Mol. Ther. Methods Clin. Dev. *6*, 1–7. https://doi.org/10.1016/j.omtm.2017.03.008.
19. Callejas, D., Mann, C.J., Ayuso, E., Lage, R., Grifoll, I., Roca, C., Andaluz, A., Ruiz-de Gopegui, R., Montané, J., Muñoz, S., et al. (2013). Treatment of Diabetes and Long-Term Survival After Insulin and Glucokinase Gene Therapy. Diabetes *62*, 1718–1729. https://doi.org/10.2337/db12-1113.
20. Schaum, N., Lehallier, B., Hahn, O., Pálovics, R., Hosseinzadeh, S., Lee, S.E., Sit, R., Lee, D.P., Losada, P.M., Zardeneta, M.E., et al. (2020). Ageing hallmarks exhibit organ-specific temporal signatures. Nature *583*, 596–602. https://doi.org/10.1038/s41586-020-2499-y.
21. Yu, Q., Xiao, H., Jedrychowski, M.P., Schweppe, D.K., Navarrete-Perea, J., Knott, J., Rogers, J., Chouchani, E.T., and Gygi, S.P. (2020). Sample multiplexing for targeted pathway proteomics in aging mice. Proc. Natl. Acad. Sci. USA *117*, 9723–9732. https://doi.org/10.1073/pnas.1919410117.
22. Talukdar, S., and Kharitonenkov, A. (2021). FGF19 and FGF21: In NASH we trust. Mol. Metab. *46*, 101152. https://doi.org/10.1016/j.molmet.2020.101152.
23. Tyshkovskiy, A., Bozaykut, P., Borodinova, A.A., Gerashchenko, M.V., Ables, G.P., Garratt, M., Khaitovich, P., Clish, C.B., Miller, R.A., and Gladyshev, V.N. (2019). Identification and Application of Gene Expression Signatures Associated with Lifespan Extension. Cel Metab. *30*, 573–593.e8. https://doi.org/10.1016/j.cmet.2019.06.018.
24. Cai, J., Wang, F., and Shao, M. (2023). The Emerging Importance of Mitochondria in White Adipocytes: Neither Last nor Least. Endocrinol. Metab. (Seoul). *38*, 493–503. https://doi.org/10.3803/EnM.2023.1813.
25. Macêdo, A.P.A., da Silva, A.S.R., Muñoz, V.R., Ropelle, E.R., and Pauli, J.R. (2021). Mitochondrial dysfunction plays an essential role in remodeling aging adipose tissue. Mech. Ageing Dev. *200*, 111598. https://doi.org/10.1016/j.mad.2021.111598.
26. Chang, J.S. (2023). Recent insights into the molecular mechanisms of simultaneous fatty acid oxidation and synthesis in brown adipocytes. Front. Endocrinol. *14*, 1106544. https://doi.org/10.3389/fendo.2023.1106544.
27. Yu, L., Wan, Q., Liu, Q., Fan, Y., Zhou, Q., Skowronski, A.A., Wang, S., Shao, Z., Liao, C.-Y., Ding, L., et al. (2024). IgG is an aging factor that drives adipose tissue fibrosis and metabolic decline. Cel Metab. *36*, 793–807.e5. https://doi.org/10.1016/j.cmet.2024.01.015.
28. Akhmetshina, A., Palumbo, K., Dees, C., Bergmann, C., Venalis, P., Zerr, P., Horn, A., Kireva, T., Beyer, C., Zwerina, J., et al. (2012). Activation of canonical Wnt signalling is required for TGF-β-mediated fibrosis. Nat. Commun. *3*, 735. https://doi.org/10.1038/ncomms1734.
29. Khan, T., Muise, E.S., Iyengar, P., Wang, Z.V., Chandalia, M., Abate, N., Zhang, B. B., Bonaldo, P., Chua, S., and Scherer, P.E. (2009). Metabolic Dysregulation and Adipose Tissue Fibrosis: Role of Collagen VI. Mol. Cel. Biol. *29*, 1575–1591. https://doi.org/10.1128/MCB.01300-08.
30. Zhang, H., Tsui, C.K., Garcia, G., Joe, L.K., Wu, H., Maruichi, A., Fan, W., Pandovski, S., Yoon, P.H., Webster, B.M., et al. (2024). The extracellular matrix integrates mitochondrial homeostasis. Cell *187*, 4289–4304.e26. https://doi.org/10.1016/j.cell.2024.05.057.
31. Bahn, Y.J., Wang, Y., Dagur, P., Scott, N., Cero, C., Long, K.T., Nguyen, N., Cypess, A.M., and Rane, S.G. (2024). TGF-β antagonism synergizes with PPARγ agonism to reduce fibrosis and enhance beige adipogenesis. Mol. Metab. *90*, 102054. https://doi.org/10.1016/j.molmet.2024.102054.
32. Sun, K., Li, X., and Scherer, P.E. (2023). Extracellular Matrix (ECM) and Fibrosis in Adipose Tissue: Overview and Perspectives. In Comprehensive Physiology (Wiley), pp. 4387–4407. https://doi.org/10.1002/cphy.c220020.
33. Zhang, Y.-X., Ou, M.-Y., Yang, Z.-H., Sun, Y., Li, Q.-F., and Zhou, S.-B. (2023). Adipose tissue aging is regulated by an altered immune system. Front. Immunol. *14*, 1125395. https://doi.org/10.3389/fimmu.2023.1125395.
34. Andonian, B.J., Hippensteel, J.A., Abuabara, K., Boyle, E.M., Colbert, J.F., Devinney, M.J., Faye, A.S., Kochar, B., Lee, J., Litke, R., et al. (2024). Inflammation and aging-related disease: A transdisciplinary inflammaging framework. Geroscience *47*, 515–542. https://doi.org/10.1007/s11357-024-01364-0.
35. Minamino, T., Orimo, M., Shimizu, I., Kunieda, T., Yokoyama, M., Ito, T., Nojima, A., Nabetani, A., Oike, Y., Matsubara, H., et al. (2009). A crucial role for adipose

tissue p53 in the regulation of insulin resistance. Nat. Med. *15*, 1082–1087. https://doi.org/10.1038/nm.2014.

36. Hallenborg, P., Fjære, E., Liaset, B., Petersen, R.K., Murano, I., Sonne, S.B., Falkerslev, M., Winther, S., Jensen, B.A.H., Ma, T., et al. (2016). p53 regulates expression of uncoupling protein 1 through binding and repression of PPARγ coactivator-1α. Am. J. Physiology-Endocrinology Metab. *310*, E116–E128. https://doi.org/10.1152/ajpendo.00119.2015.

37. Okita, N., Ishikawa, N., Mizunoe, Y., Oku, M., Nagai, W., Suzuki, Y., Matsushima, S., Mikami, K., Okado, H., Sasaki, T., and Higami, Y. (2014). Inhibitory effect of p53 on mitochondrial content and function during adipogenesis. Biochem. Biophys. Res. Commun. *446*, 91–97. https://doi.org/10.1016/j.bbrc.2014.02.059.

38. Fu, W., Liu, Y., Sun, C., and Yin, H. (2019). Transient p53 inhibition sensitizes aged white adipose tissue for beige adipocyte recruitment by blocking mitophagy. FASEB J. *33*, 844–856. https://doi.org/10.1096/fj.201800577R.

39. Salminen, A., Kauppinen, A., and Kaarniranta, K. (2017). FGF21 activates AMPK signaling: impact on metabolic regulation and the aging process. J. Mol. Med. *95*, 123–131. https://doi.org/10.1007/s00109-016-1477-1.

40. Hossam Abdelmonem, B., Abdelaal, N.M., Anwer, E.K.E., Rashwan, A.A., Hussein, M.A., Ahmed, Y.F., Khashana, R., Hanna, M.M., and Abdelnaser, A. (2024). Decoding the Role of CYP450 Enzymes in Metabolism and Disease: A Comprehensive Review. Biomedicines *12*, 1467. https://doi.org/10.3390/biomedicines12071467.

41. Ni, K.-D., and Liu, J.-Y. (2021). The Functions of Cytochrome P450 ω-hydroxylases and the Associated Eicosanoids in Inflammation-Related Diseases. Front. Pharmacol. *12*, 716801. https://doi.org/10.3389/fphar.2021.716801.

42. Wright, W.C., Chenge, J., and Chen, T. (2019). Structural perspectives of the CYP3A family and their small molecule modulators in drug metabolism. Liver Res. *3*, 132–142. https://doi.org/10.1016/j.livres.2019.08.001.

43. Heintz, M.M., Kumar, R., Maner-Smith, K.M., Ortlund, E.A., and Baldwin, W.S. (2022). Age- and Diet-Dependent Changes in Hepatic Lipidomic Profiles of Phospholipids in Male Mice: Age Acceleration in Cyp2b-Null Mice. J. Lipids *2022*, 1–17. https://doi.org/10.1155/2022/7122738.

44. Vyskočilová, E., Szotáková, B., Skálová, L., Bártíková, H., Hlaváčová, J., and Boušová, I. (2013). Age-Related Changes in Hepatic Activity and Expression of Detoxification Enzymes in Male Rats. Biomed. Res. Int. *2013*, 1–10. https://doi.org/10.1155/2013/408573.

45. Xu, S., Hou, D., Liu, J., and Ji, L. (2018). Age-associated changes in GSH S-transferase gene/proteins in livers of rats. Redox Rep. *23*, 213–218. https://doi.org/10.1080/13510002.2018.1546985.

46. Kocarek, T.A., Duanmu, Z., Fang, H.-L., and Runge-Morris, M. (2008). Age- and sex-dependent expression of multiple murine hepatic hydroxysteroid sulfotransferase (SULT2A) genes. Biochem. Pharmacol. *76*, 1036–1046. https://doi.org/10.1016/j.bcp.2008.07.032.

47. Grevengoed, T.J., Trammell, S.A.J., McKinney, M.K., Petersen, N., Cardone, R.L., Svenningsen, J.S., Ogasawara, D., Nexøe-Larsen, C.C., Knop, F.K., Schwartz, T. W., et al. (2019). *N* -acyl taurines are endogenous lipid messengers that improve glucose homeostasis. Proc. Natl. Acad. Sci. USA *116*, 24770–24778. https://doi.org/10.1073/pnas.1916288116.

48. Hunt, M.C., Siponen, M.I., and Alexson, S.E.H. (2012). The emerging role of acyl-CoA thioesterases and acyltransferases in regulating peroxisomal lipid metabolism. Biochim. Biophys. Acta (Bba) - Mol. Basis Dis. *1822*, 1397–1410. https://doi.org/10.1016/j.bbadis.2012.03.009.

49. Makwana, K., Gosai, N., Poe, A., and Kondratov, R.V. (2019). Calorie restriction reprograms diurnal rhythms in protein translation to regulate metabolism. FASEB J. *33*, 4473–4489. https://doi.org/10.1096/fj.201802167R.

50. Liu, F., Chen, J., Li, Z., and Meng, X. (2022). Recent Advances in Epigenetics of Age-Related Kidney Diseases. Genes (Basel) *13*, 796. https://doi.org/10.3390/genes13050796.

51. O'Sullivan, E.D., Hughes, J., and Ferenbach, D.A. (2017). Renal Aging: Causes and Consequences. J. Am. Soc. Nephrol. *28*, 407–420. https://doi.org/10.1681/ASN.2015121308.

52. Marquez-Exposito, L., Tejedor-Santamaria, L., Valentijn, F.A., Tejera-Muñoz, A., Rayego-Mateos, S., Marchant, V., Rodrigues-Diez, R.R., Rubio-Soto, I., Knoppert, S.N., Ortiz, A., et al. (2022). Oxidative Stress and Cellular Senescence Are Involved in the Aging Kidney. Antioxidants *11*, 301. https://doi.org/10.3390/antiox11020301.

53. Chiao, Y.A., and Rabinovitch, P.S. (2015). The Aging Heart. Cold Spring Harb. Perspect. Med. *5*, a025148. https://doi.org/10.1101/cshperspect.a025148.

54. Bart, N.K., Bianchi, G., Cuddy, S.A.M., Goyal, P., Griffin, J.M., Hummel, S.L., Macdonald, P., Maurer, M., Montgomery, E., Nanne, M.G., et al. (2025). Cardiac Amyloidosis in Older Adults With a Focus on Frailty. JACC: Adv. *4*, 101784. https://doi.org/10.1016/j.jacadv.2025.101784.

55. Kwak, H.-B. (2013). Aging, exercise, and extracellular matrix in the heart. J. Exerc. Rehabil. *9*, 338–347. https://doi.org/10.12965/jer.130049.

56. Distefano, G., and Goodpaster, B.H. (2018). Effects of Exercise and Aging on Skeletal Muscle. Cold Spring Harb. Perspect. Med. *8*, a029785. https://doi.org/10.1101/cshperspect.a029785.

57. Muraine, L., Bensalah, M., Butler-Browne, G., Bigot, A., Trollet, C., Mouly, V., and Negroni, E. (2023). Update on anti-fibrotic pharmacotherapies in skeletal muscle disease. Curr. Opin. Pharmacol. *68*, 102332. https://doi.org/10.1016/j.coph.2022.102332.

58. Lieber, R.L., and Ward, S.R. (2013). Cellular Mechanisms of Tissue Fibrosis. 4. Structural and functional consequences of skeletal muscle fibrosis. Am. J. Physiology-Cell Physiol. *305*, C241–C252. https://doi.org/10.1152/ajpcell.00173.2013.

59. Ubaida-Mohien, C., Lyashkov, A., Gonzalez-Freire, M., Tharakan, R., Shardell, M., Moaddel, R., Semba, R.D., Chia, C.W., Gorospe, M., Sen, R., and Ferrucci, L. (2019). Discovery proteomics in aging human skeletal muscle finds change in spliceosome, immunity, proteostasis and mitochondria. Elife *8*, e49874. https://doi.org/10.7554/eLife.49874.

60. Figueiredo, V.C., D'Souza, R.F., Van Pelt, D.W., Lawrence, M.M., Zeng, N., Markworth, J.F., Poppitt, S.D., Miller, B.F., Mitchell, C.J., McCarthy, J.J., et al. (2021). Ribosome biogenesis and degradation regulate translational capacity during muscle disuse and reloading. J. Cachexia Sarcopenia Muscle *12*, 130–143. https://doi.org/10.1002/jcsm.12636.

61. Koopman, R., and van Loon, L.J.C. (2009). Aging, exercise, and muscle protein metabolism. J. Appl. Physiol. *106*, 2040–2048. https://doi.org/10.1152/japplphysiol.91551.2008.

62. Davidsohn, N., Pezone, M., Vernet, A., Graveline, A., Oliver, D., Slomovic, S., Punthambaker, S., Sun, X., Liao, R., Bonventre, J.V., and Church, G.M. (2019). A single combination gene therapy treats multiple age-related diseases. Proc. Natl. Acad. Sci. USA *116*, 23505–23511. https://doi.org/10.1073/pnas.1910073116.

63. Thompson, K.E., Guillot, M., Graziano, M.J., Mangipudy, R.S., and Chadwick, K.D. (2021). Pegbelfermin, a PEGylated FGF21 analogue, has pharmacology without bone toxicity after 1-year dosing in skeletally-mature monkeys. Toxicol. Appl. Pharmacol. *428*, 115673. https://doi.org/10.1016/j.taap.2021.115673.

64. Murman, D. (2015). The Impact of Age on Cognition. Semin. Hear. *36*, 111–121. https://doi.org/10.1055/s-0035-1555115.

65. Cunnane, S.C., Trushina, E., Morland, C., Prigione, A., Casadesus, G., Andrews, Z. B., Beal, M.F., Bergersen, L.H., Brinton, R.D., de la Monte, S., et al. (2020). Brain energy rescue: an emerging therapeutic concept for neurodegenerative disorders of ageing. Nat. Rev. Drug Discov. *19*, 609–633. https://doi.org/10.1038/s41573-020-0072-x.

66. Butterfield, D.A., and Halliwell, B. (2019). Oxidative stress, dysfunctional glucose metabolism and Alzheimer disease. Nat. Rev. Neurosci. *20*, 148–160. https://doi.org/10.1038/s41583-019-0132-6.

67. Inagaki, T., Dutchak, P., Zhao, G., Ding, X., Gautron, L., Parameswara, V., Li, Y., Goetz, R., Mohammadi, M., Esser, V., et al. (2007). Endocrine Regulation of the Fasting Response by PPARα-Mediated Induction of Fibroblast Growth Factor 21. Cel Metab. *5*, 415–425. https://doi.org/10.1016/j.cmet.2007.05.003.

68. Lin, Z., Tian, H., Lam, K.S.L., Lin, S., Hoo, R.C.L., Konishi, M., Itoh, N., Wang, Y., Bornstein, S.R., Xu, A., and Li, X. (2013). Adiponectin Mediates the Metabolic Effects of FGF21 on Glucose Homeostasis and Insulin Sensitivity in Mice. Cel Metab. *17*, 779–789. https://doi.org/10.1016/j.cmet.2013.04.005.

69. Jensen, N.J., Wodschow, H.Z., Nilsson, M., and Rungby, J. (2020). Effects of Ketone Bodies on Brain Metabolism and Function in Neurodegenerative Diseases. Int. J. Mol. Sci. *21*, 8767. https://doi.org/10.3390/ijms21228767.

70. Hafner, A.-S., Donlin-Asp, P.G., Leitch, B., Herzog, E., and Schuman, E.M. (2019). Local protein synthesis is a ubiquitous feature of neuronal pre- and postsynaptic compartments. Science *364*, eaau3644. https://doi.org/10.1126/science.aau3644.

71. Perrone-Capano, C., Volpicelli, F., Penna, E., Chun, J.T., and Crispino, M. (2021). Presynaptic protein synthesis and brain plasticity: From physiology to neuropathology. Prog. Neurobiol. *202*, 102051. https://doi.org/10.1016/j.pneurobio.2021.102051.

72. Cai, Q., and Tammineni, P. (2017). Mitochondrial Aspects of Synaptic Dysfunction in Alzheimer's Disease. J. Alzheimer's Dis. *57*, 1087–1103. https://doi.org/10.3233/JAD-160726.

73. Rangaraju, V., Lauterbach, M., and Schuman, E.M. (2019). Spatially Stable Mitochondrial Compartments Fuel Local Translation during Plasticity. Cell *176*, 73–84.e15. https://doi.org/10.1016/j.cell.2018.12.013.

74. Hsuchou, H., Pan, W., and Kastin, A.J. (2007). The fasting polypeptide FGF21 can enter brain from blood. Peptides *28*, 2382–2386. https://doi.org/10.1016/J.PEPTIDES.2007.10.007.

75. Hamed, S.A. (2017). Brain injury with diabetes mellitus: evidence, mechanisms and treatment implications. Expert Rev. Clin. Pharmacol. *10*, 409–428. https://doi.org/10.1080/17512433.2017.1293521.

76. Misiak, B., Leszek, J., and Kiejna, A. (2012). Metabolic syndrome, mild cognitive impairment and Alzheimer's disease–the emerging role of systemic low-grade inflammation and adiposity. Brain Res. Bull. *89*, 144–149. https://doi.org/10.1016/J.BRAINRESBULL.2012.08.003.

77. Deery, H.A., Liang, E., Di Paolo, R., Voigt, K., Murray, G., Siddiqui, M.N., Egan, G. F., Moran, C., and Jamadar, S.D. (2024). Peripheral insulin resistance attenuates cerebral glucose metabolism and impairs working memory in healthy adults. Npj Metab. Health Dis. *2*, 17. https://doi.org/10.1038/s44324-024-00019-0.

78. Amorim, J.A., Coppotelli, G., Rolo, A.P., Palmeira, C.M., Ross, J.M., and Sinclair, D.A. (2022). Mitochondrial and metabolic dysfunction in ageing and age-related diseases. Nat. Rev. Endocrinol. *18*, 243–258. https://doi.org/10.1038/s41574-021-00626-7.

79. Solyga, M., Majumdar, A., and Besse, F. (2024). Regulating translation in aging: from global to gene-specific mechanisms. EMBO Rep. *25*, 5265–5276. https://doi.org/10.1038/s44319-024-00315-2.

80. Fon Tacer, K., Bookout, A.L., Ding, X., Kurosu, H., John, G.B., Wang, L., Goetz, R., Mohammadi, M., Kuro-o, M., Mangelsdorf, D.J., and Kliewer, S.A. (2010). Research Resource: Comprehensive Expression Atlas of the Fibroblast Growth Factor System in Adult Mouse. Mol. Endocrinol. *24*, 2050–2064. https://doi.org/10.1210/me.2010-0142.

81. Owen, B.M., Ding, X., Morgan, D.A., Coate, K.C., Bookout, A.L., Rahmouni, K., Kliewer, S.A., and Mangelsdorf, D.J. (2014). FGF21 Acts Centrally to Induce Sympathetic Nerve Activity, Energy Expenditure, and Weight Loss. Cel Metab. *20*, 670–677. https://doi.org/10.1016/j.cmet.2014.07.012.

82. Song, P., Zechner, C., Hernandez, G., Cánovas, J., Xie, Y., Sondhi, V., Wagner, M., Stadlbauer, V., Horvath, A., Leber, B., et al. (2018). The Hormone FGF21 Stimulates Water Drinking in Response to Ketogenic Diet and Alcohol. Cel Metab. *27*, 1338–1347.e4. https://doi.org/10.1016/j.cmet.2018.04.001.

83. Zhou, B., Claflin, K.E., Flippo, K.H., Sullivan, A.I., Asghari, A., Tadinada, S.M., Jensen-Cody, S.O., Abel, T., and Potthoff, M.J. (2022). Central FGF21 production regulates memory but not peripheral metabolism. Cell Rep. *40*, 111239. https://doi.org/10.1016/j.celrep.2022.111239.

84. Chen, Z., Yang, L., Liu, Y., Huang, P., Song, H., and Zheng, P. (2022). The potential function and clinical application of FGF21 in metabolic diseases. Front. Pharmacol. *13*, 1089214. https://doi.org/10.3389/fphar.2022.1089214.

85. Holland, W.L., Adams, A.C., Brozinick, J.T., Bui, H.H., Miyauchi, Y., Kusminski, C. M., Bauer, S.M., Wade, M., Singhal, E., Cheng, C.C., et al. (2013). An FGF21-Adiponectin-Ceramide Axis Controls Energy Expenditure and Insulin Action in Mice. Cel Metab. *17*, 790–797. https://doi.org/10.1016/j.cmet.2013.03.019.

86. Jing, H., Tang, S., Lin, S., Liao, M., Chen, H., Fan, Y., and Zhou, J. (2020). Adiponectin in renal fibrosis. Aging *12*, 4660–4672. https://doi.org/10.18632/aging.102811.

87. Zha, D., Wu, X., and Gao, P. (2017). Adiponectin and Its Receptors in Diabetic Kidney Disease: Molecular Mechanisms and Clinical Potential. Endocrinology *158*, 2022–2034. https://doi.org/10.1210/en.2016-1765.

88. Nanayakkara, G., Kariharan, T., Wang, L., Zhong, J., and Amin, R. (2012). The cardio-protective signaling and mechanisms of adiponectin. Am. J. Cardiovasc. Dis. *2*, 253–266.

89. Abou-Samra, M., Selvais, C.M., Dubuisson, N., and Brichard, S.M. (2020). Adiponectin and Its Mimics on Skeletal Muscle: Insulin Sensitizers, Fat Burners, Exercise Mimickers, Muscling Pills or Everything Together? Int. J. Mol. Sci. *21*, 2620. https://doi.org/10.3390/ijms21072620.

90. Zhang, Y., Xie, Y., Berglund, E.D., Coate, K.C., He, T.T., Katafuchi, T., Xiao, G., Potthoff, M.J., Wei, W., Wan, Y., et al. (2012). The starvation hormone, fibroblast growth factor-21, extends lifespan in mice. Elife *1*, e00065. https://doi.org/10.7554/eLife.00065.

91. Youm, Y.-H., Gliniak, C., Zhang, Y., Dlugos, T., Scherer, P.E., and Dixit, V.D. (2025). Enhanced paracrine action of FGF21 in stromal cells delays thymic aging. Nat. Aging *5*, 576–587. https://doi.org/10.1038/s43587-025-00813-5.

92. Youm, Y.-H., Horvath, T.L., Mangelsdorf, D.J., Kliewer, S.A., and Dixit, V.D. (2016). Prolongevity hormone FGF21 protects against immune senescence by delaying age-related thymic involution. Proc. Natl. Acad. Sci. USA *113*, 1026–1031. https://doi.org/10.1073/pnas.1514511113.

93. Gliniak, C.M., Gordillo, R., Youm, Y.-H., Lin, Q., Crewe, C., Zhang, Z., Field, B.C., Fujikawa, T., Virostek, M., Zhao, S., et al. (2025). FGF21 promotes longevity in diet-induced obesity through metabolic benefits independent of growth suppression. Cel Metab. *37*, 1547–1567.e6. https://doi.org/10.1016/j.cmet.2025.05.011.

94. Wedemeyer, S.A., Jones, N.E., Raza, I.G.A., Green, F.M., Xiao, Y., Semwal, M.K., Garza, A.K., Archuleta, K.S., Wimberly, K.L., Venables, T., et al. (2025). Paracrine FGF21 dynamically modulates mTOR signaling to regulate thymus function across the lifespan. Nat. Aging *5*, 588–606. https://doi.org/10.1038/s43587-024-00801-1.

95. Inagaki, T., Lin, V.Y., Goetz, R., Mohammadi, M., Mangelsdorf, D.J., and Kliewer, S.A. (2008). Inhibition of Growth Hormone Signaling by the Fasting-Induced Hormone FGF21. Cel Metab. *8*, 77–83. https://doi.org/10.1016/j.cmet.2008.05.006.

96. Wei, W., Dutchak, P.A., Wang, X., Ding, X., Wang, X., Bookout, A.L., Goetz, R., Mohammadi, M., Gerard, R.D., Dechow, P.C., et al. (2012). Fibroblast growth factor 21 promotes bone loss by potentiating the effects of peroxisome proliferator-activated receptor γ. Proc. Natl. Acad. Sci. USA *109*, 3143–3148. https://doi.org/10.1073/pnas.1200797109.

97. Hill, C.M., Albarado, D.C., Coco, L.G., Spann, R.A., Khan, M.S., Qualls-Creekmore, E., Burk, D.H., Burke, S.J., Collier, J.J., Yu, S., et al. (2022). FGF21 is required for protein restriction to extend lifespan and improve metabolic health in male mice. Nat. Commun. *13*, 1897. https://doi.org/10.1038/s41467-022-29499-8.

98. Lees, E.K., Król, E., Grant, L., Shearer, K., Wyse, C., Moncur, E., Bykowska, A.S., Mody, N., Gettys, T.W., and Delibegovic, M. (2014). Methionine restriction restores a younger metabolic phenotype in adult mice with alterations in fibroblast growth factor 21. Aging Cell *13*, 817–827. https://doi.org/10.1111/acel.12238.

99. Anderson, J.M., Arnold, W.D., Huang, W., Ray, A., Owendoff, G., and Cao, L. (2024). Long-term effects of a fat-directed FGF21 gene therapy in aged female mice. Gene Ther. *31*, 95–104. https://doi.org/10.1038/s41434-023-00422-0.

100. Callejas, D., Mann, C.J., Ayuso, E., Lage, R., Grifoll, I., Roca, C., Andaluz, A., Ruiz-de Gopegui, R., Montané, J., Muñoz, S., et al. (2013). Treatment of diabetes and long-term survival after insulin and glucokinase gene therapy. Diabetes *62*, 1718–1729.

101. Kuzmin, D.A., Shutova, M.V., Johnston, N.R., Smith, O.P., Fedorin, V.V., Kukushkin, Y.S., van der Loo, J.C.M., and Johnstone, E.C. (2021). The clinical landscape for AAV gene therapies. Nat. Rev. Drug Discov. *20*, 173–174. https://doi.org/10.1038/d41573-021-00017-7.

102. Wang, D., Tai, P.W.L., and Gao, G. (2019). Adeno-associated virus vector as a platform for gene therapy delivery. Nat. Rev. Drug Discov. *18*, 358–378. https://doi.org/10.1038/S41573-019-0012-9.

103. Mendell, J.R., Al-Zaidy, S.A., Rodino-Klapac, L.R., Goodspeed, K., Gray, S.J., Kay, C.N., Boye, S.L., Boye, S.E., George, L.A., Salabarria, S., et al. (2021). Current

Clinical Applications of In Vivo Gene Therapy with AAVs. Mol. Ther. *29*, 464–488. https://doi.org/10.1016/J.YMTHE.2020.12.007.

104. Gaudet, D., Stroes, E.S., Méthot, J., Brisson, D., Tremblay, K., Bernelot Moens, S.J., Iotti, G., Rastelletti, I., Ardigo, D., Corzo, D., et al. (2016). Long-Term Retrospective Analysis of Gene Therapy with Alipogene Tiparvovec and Its Effect on Lipoprotein Lipase Deficiency-Induced Pancreatitis. Hum. Gene Ther. *27*, 916–925. https://doi.org/10.1089/HUM.2015.158.

105. Mueller, C., Gernoux, G., Gruntman, A.M., Borel, F., Reeves, E.P., Calcedo, R., Rouhani, F.N., Yachnis, A., Humphries, M., Campbell-Thompson, M., et al. (2017). 5 Year Expression and Neutrophil Defect Repair after Gene Therapy in Alpha-1 Antitrypsin Deficiency. Mol. Ther. *25*, 1387–1394. https://doi.org/10.1016/J.YMTHE.2017.03.029.

106. Buchlis, G., Podsakoff, G.M., Radu, A., Hawk, S.M., Flake, A.W., Mingozzi, F., and High, K.A. (2012). Factor IX expression in skeletal muscle of a severe hemophilia B patient 10 years after AAV-mediated gene transfer. Blood *119*, 3038–3041. https://doi.org/10.1182/BLOOD-2011-09-382317.

107. Manno, C.S., Chew, A.J., Hutchison, S., Larson, P.J., Herzog, R.W., Arruda, V.R., Tai, S.J., Ragni, M.V., Thompson, A., Ozelo, M., et al. (2003). AAV-mediated factor IX gene transfer to skeletal muscle in patients with severe hemophilia B. Blood *101*, 2963–2972. https://doi.org/10.1182/BLOOD-2002-10-3296.

108. Greig, J.A., Calcedo, R., Grant, R.L., Peng, H., Medina-Jaszek, C.A., Ahonkhai, O., Qin, Q., Roy, S., Tretiakova, A.P., and Wilson, J.M. (2016). Intramuscular administration of AAV overcomes pre-existing neutralizing antibodies in rhesus macaques. Vaccine *34*, 6323–6329. https://doi.org/10.1016/J.VACCINE.2016.10.053.

109. Flotte, T.R. (2020). Revisiting the "New" Inflammatory Toxicities of Adeno-Associated Virus Vectors. Hum. Gene Ther. *31*, 398–399. https://doi.org/10.1089/HUM.2020.29117.TRF.

110. Shen, W., Liu, S., and Ou, L. (2022). rAAV immunogenicity, toxicity, and durability in 255 clinical trials: A meta-analysis. Front. Immunol. *13*, 1001263. https://doi.org/10.3389/FIMMU.2022.1001263.

111. Ertl, H.C.J. (2022). Immunogenicity and toxicity of AAV gene therapy. Front. Immunol. *13*, 975803. https://doi.org/10.3389/FIMMU.2022.975803.

112. Duan, D. (2023). Lethal immunotoxicity in high-dose systemic AAV therapy. Mol. Ther. *31*, 3123–3126. https://doi.org/10.1016/J.YMTHE.2023.10.015.

113. Flotte, T.R., Trapnell, B.C., Humphries, M., Carey, B., Calcedo, R., Rouhani, F., Campbell-Thompson, M., Yachnis, A.T., Sandhaus, R.A., McElvaney, N.G., et al. (2011). Phase 2 clinical trial of a recombinant adeno-associated viral vector expressing α1-antitrypsin: interim results. Hum. Gene Ther. *22*, 1239–1247. https://doi.org/10.1089/HUM.2011.053.

114. Brantly, M.L., Chulay, J.D., Wang, L., Mueller, C., Humphries, M., Spencer, L.T., Rouhani, F., Conlon, T.J., Calcedo, R., Betts, M.R., et al. (2009). Sustained transgene expression despite T lymphocyte responses in a clinical trial of rAAV1-AAT gene therapy. Proc. Natl. Acad. Sci. USA *106*, 16363–16368. https://doi.org/10.1073/PNAS.0904514106.

115. Costa-Verdera, H., Unzu, C., Valeri, E., Adriouch, S., González Aseguinolaza, G., Mingozzi, F., and Kajaste-Rudnitski, A. (2023). Understanding and Tackling Immune Responses to Adeno-Associated Viral Vectors. Hum. Gene Ther. *34*, 836–852. https://doi.org/10.1089/HUM.2023.119.

116. Ayuso, E., Mingozzi, F., Montane, J., Leon, X., Anguela, X.M., Haurigot, V., Edmonson, S.A., Africa, L., Zhou, S., High, K.A., et al. (2010). High AAV vector purity results in serotype- and tissue-independent enhancement of transduction efficiency. Gene Ther. *17*, 503–510. https://doi.org/10.1038/GT.2009.157.

117. Zhong, Y., Lee, K., Deng, Y., Ma, Y., Chen, Y., Li, X., Wei, C., Yang, S., Wang, T., Wong, N.J., et al. (2019). Arctigenin attenuates diabetic kidney disease through the activation of PP2A in podocytes. Nat. Commun. *10*, 4523. https://doi.org/10.1038/s41467-019-12433-w.

YMTHE, Volume *34*

Supplemental Information

AAV-mediated FGF21 gene therapy promotes health span extension by whole-body tissue-specific adaptations

Veronica Jimenez, Victor Sacristan, Miquel Garcia, Claudia Jambrina, Estefania Casana, Sergio Muñoz, Laia Vilà, Ignasi Grass, Maria Luisa Jaén, Carles Roca, Xavier León, Sara Marcó, Maria Molas, Albert Ribera, Ivet Elias, Jordi Rodó, Tura Ferré, and Fatima Bosch

Figure S1

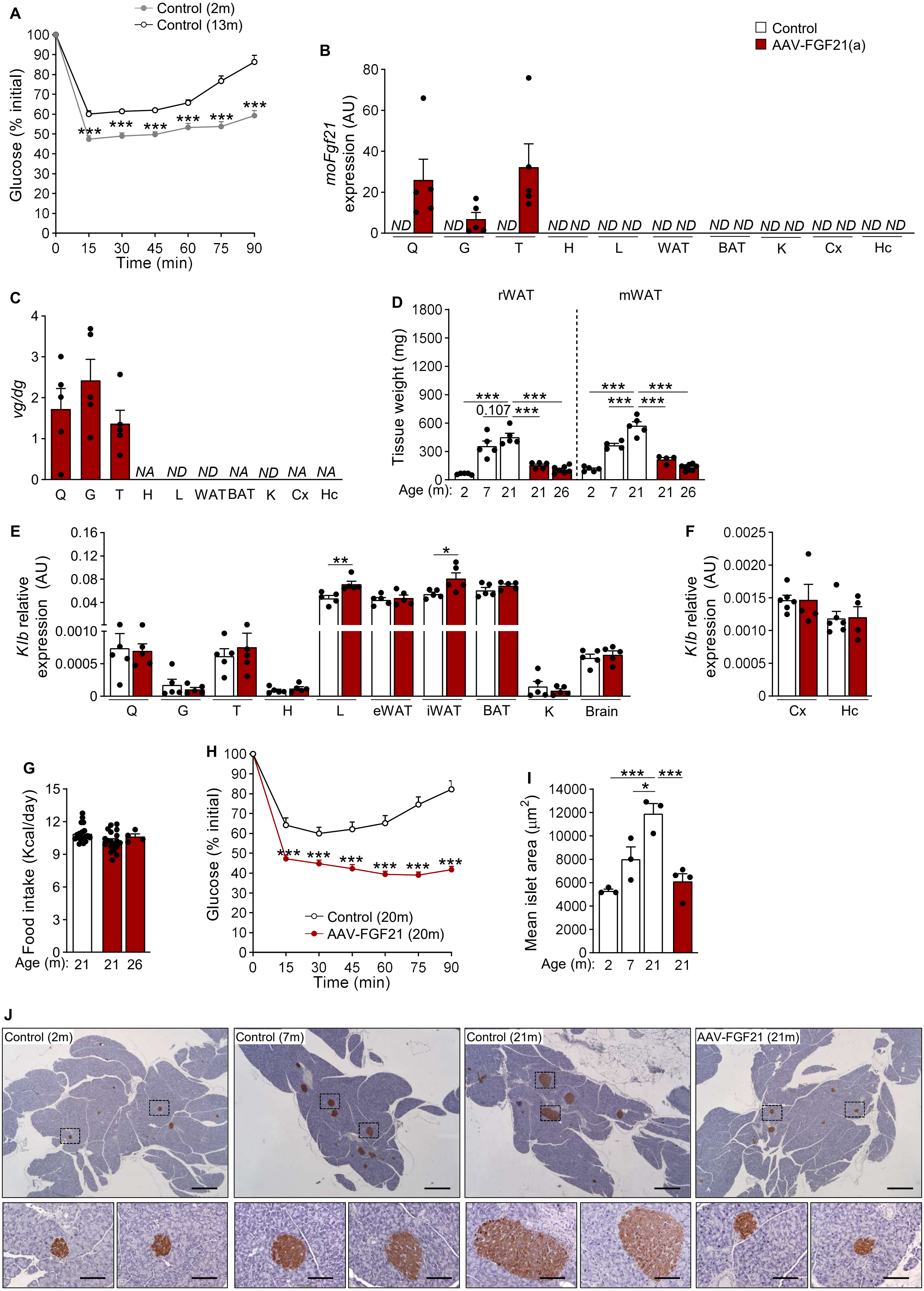

A
Control (2m)
Control (13m)
Glucose (% initial)
Time (min)
B
Control
AAV-FGF21(a)
moFgf21 expression (AU)
Q G T H L WAT BAT K Cx Hc
ND
C
vg/dg
NA
ND
D
rWAT
mWAT
Tissue weight (mg)
Age (m): 2 7 21 21 26 2 7 21 21 26
0.107
E
Klb relative expression (AU)
Q G T H L eWAT iWAT BAT K Brain
F
Cx Hc
G
Food intake (Kcal/day)
Age (m): 21 21 26
H
Control (20m)
AAV-FGF21 (20m)
Time (min)
I
Mean islet area (µm2)
Age (m): 2 7 21 21
J
Control (2m)
Control (7m)
Control (21m)
AAV-FGF21 (21m)

Figure S1. Effects of AAV-FGF21 treatment in body weight gain and glucose metabolism in male mice. (**A**) Insulin sensitivity was determined after an intraperitoneal injection of insulin (0.75 units/kg body weight) in 2- and 13-month-old male mice. Results were calculated as the percentage of initial blood glucose levels (*n*=11-30/group). (**B**) Quantification by qRT–PCR of the expression of murine optimized Fgf21 (*moFgf21*) in the three injected skeletal muscles (Q, quadriceps; G, gastrocnemius; T, tibialis cranialis) and in the heart (H), the liver (L), white adipose tissue (WAT), brown adipose tissue (BAT), and kidney (K) in 21-month-old male mice and in cortex (Cx) and hippocampus (Hc) in 26-month-old male mice (*n*=5/group). (**C**) Vector genome copy number in the same cohorts as in (B) (*n*=5/group).(**D**) Weight of the retroperitoneal (rWAT), and mesenteric (mWAT) white adipose tissue depots in male mice (*n*=5-8/group). (**E** and **F**) Quantification by qRT–PCR of the expression of β-Klotho (*Klb*) in the same cohorts as in B and C in 21 (E) and 26-month-old (F) male mice (*n*=5/group). (**G**) Histogram depicting the food intake in male mice (*n*=4-19 cages/group). (**H**) Insulin sensitivity was determined after an intraperitoneal injection of insulin (0.75 units/kg body weight) in 21-month-old male mice. Results were calculated as the percentage of initial blood glucose levels (*n*=13-19/group). (**I**) Representative images of the immunostaining against insulin in pancreas sections of male mice. Scale bars: 400 µm. Inset scale bars: 100 µm. (**J**) Quantification of mean islet area in male mice (*n*=3-4/group). Data are expressed as the mean ± SEM. *P<0.05, **P<0.01, ***P<0.001 by one-way ANOVA with Dunnet's multiple comparison test (D,G,I) or Student's two-tailed t test (A,E,F,H). AAV-FGF21 (a), male mice were treated with AAV-FGF21 vectors at 13 months of age; AU, arbitrary units; m, months of age; ND, non-detected; NA, non-available; vg/dg, vector genomes/diploid genome.

Figure S2

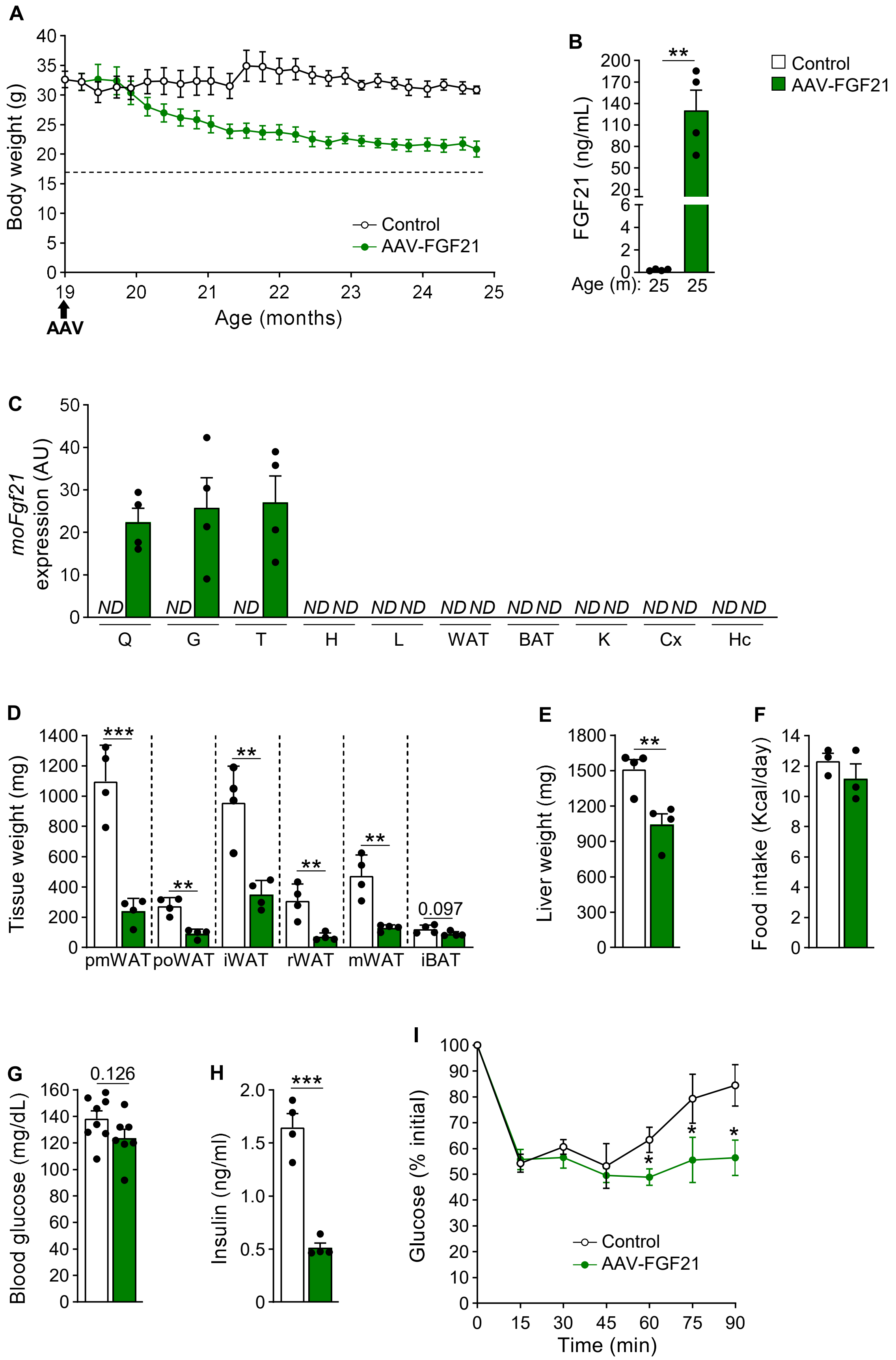

A
Body weight (g)
Control
AAV-FGF21
Age (months)
AAV
B
FGF21 (ng/mL)
Age (m): 25 25
Control
AAV-FGF21
C
moFgf21 expression (AU)
ND
Q G T H L WAT BAT K Cx Hc
D
Tissue weight (mg)
pmWAT poWAT iWAT rWAT mWAT iBAT
0.097
E
Liver weight (mg)
F
Food intake (Kcal/day)
G
Blood glucose (mg/dL)
0.126
H
Insulin (ng/ml)
I
Glucose (% initial)
Control
AAV-FGF21
Time (min)

Figure S2. Effects of AAV-FGF21 treatment in body weight gain and glucose metabolism in female mice. (**A**) Body weight follow-up in female mice treated with AAV-FGF21 vectors at 19 months of age (*n*=7-8/group). (**B**) Serum FGF21 levels in female mice (*n*=4/group). (**C**) Quantification by qRT–PCR of the expression of murine optimized Fgf21 (*moFgf21*) in the three injected skeletal muscles and in the heart (H), the liver (L), white adipose tissue (WAT), brown adipose tissue (BAT), kidney (K), cortex (Cx) and hippocampus (Hc) in 25-month-old female mice (*n*=4/group). (**D**) Weight of the parametrial (pmWAT), periovaric (poWAT), inguinal (iWAT), retroperitoneal (rWAT) and mesenteric (mWAT) white adipose tissue depots and the interscapular brown adipose tissue depot (iBAT) in female mice (*n*=4/group). (**E**) Weight of the liver in female mice (*n*=4/group). (**F**) Histogram depicting the food intake in female mice (*n*=3 cages/group). (**G** and **H**) Fed blood glucose (H, n=7-8/group) and insulin (**I**, n=4/group) levels in female mice at 21 months of age. (**I**) Insulin sensitivity was determined after an intraperitoneal injection of insulin (0.375 units/kg body weight) in 21-month-old female mice. Results were calculated as the percentage of initial blood glucose levels (*n*=6/group). Data are expressed as the mean ± SEM. *P<0.05, **P<0.01, ***P<0.001 by Student's two-tailed t test. AU, arbitrary units; m, months of age; ND, non-detected.

Figure S3

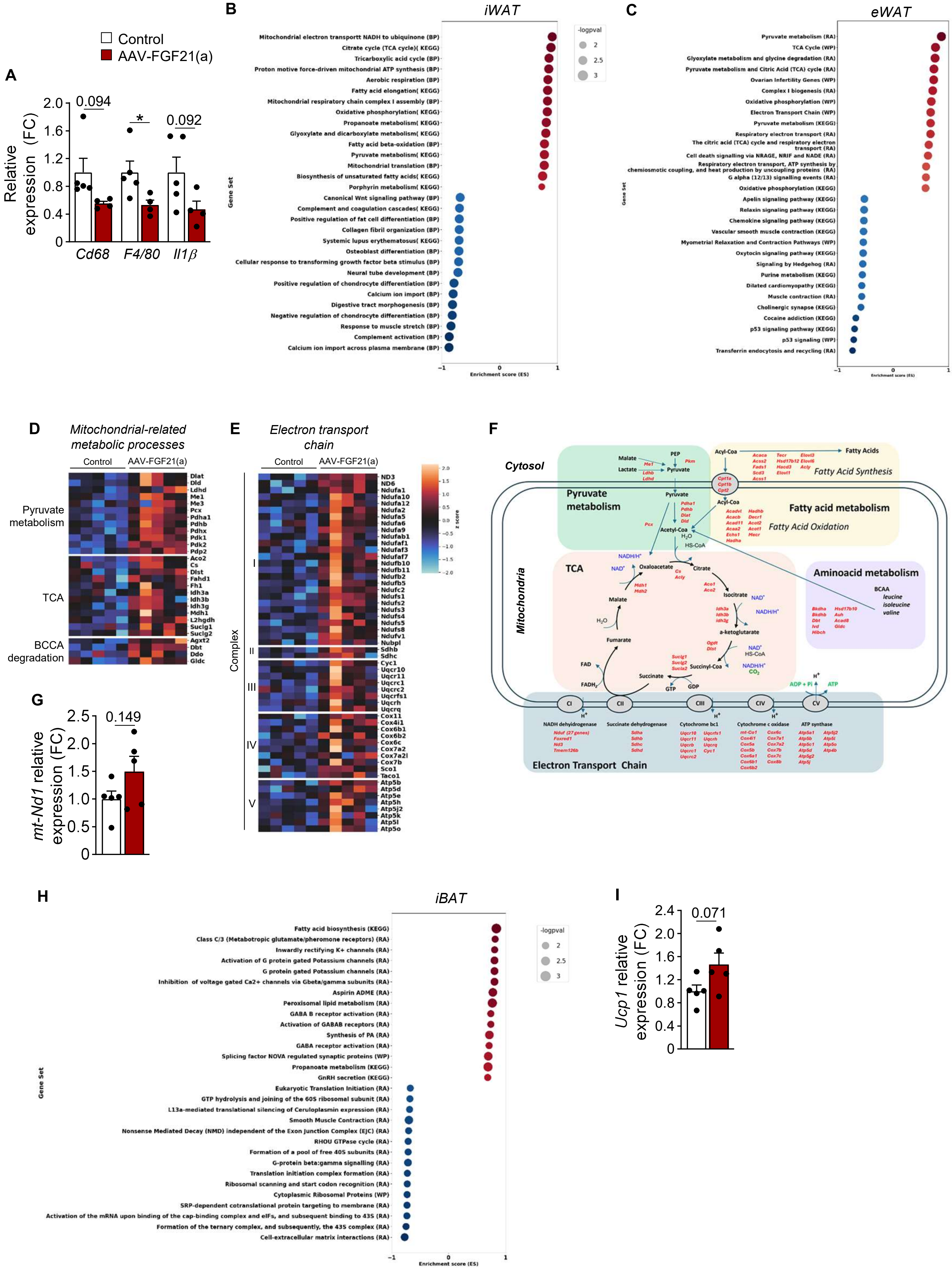

Control
AAV-FGF21(a)
A
Relative expression (FC)
0.094
*
0.092
Cd68
F4/80
Il1β
B
iWAT
-logpval
Mitochondrial electron transportt NADH to ubiquinone (BP)
Citrate cycle (TCA cycle)(KEGG)
Tricarboxylic acid cycle (BP)
Proton motive force-driven mitochondrial ATP synthesis (BP)
Aerobic respiration (BP)
Fatty acid elongation(KEGG)
Mitochondrial respiratory chain complex I assembly (BP)
Oxidative phosphorylation(KEGG)
Propanoate metabolism(KEGG)
Glyoxylate and dicarboxylate metabolism(KEGG)
Fatty acid beta-oxidation (BP)
Pyruvate metabolism(KEGG)
Mitochondrial translation (BP)
Biosynthesis of unsaturated fatty acids(KEGG)
Porphyrin metabolism(KEGG)
Canonical Wnt signaling pathway (BP)
Complement and coagulation cascades(KEGG)
Positive regulation of fat cell differentiation (BP)
Collagen fibril organization (BP)
Systemic lupus erythematosus(KEGG)
Osteoblast differentiation (BP)
Cellular response to transforming growth factor beta stimulus (BP)
Neural tube development (BP)
Positive regulation of chondrocyte differentiation (BP)
Calcium ion import (BP)
Digestive tract morphogenesis (BP)
Negative regulation of chondrocyte differentiation (BP)
Response to muscle stretch (BP)
Complement activation (BP)
Calcium ion import across plasma membrane (BP)
Gene Set
Enrichment score (ES)
C
eWAT
Pyruvate metabolism (RA)
TCA Cycle (WP)
Glyoxylate metabolism and glycine degradation (RA)
Pyruvate metabolism and Citric Acid (TCA) cycle (RA)
Ovarian Infertility Genes (WP)
Complex I biogenesis (RA)
Oxidative phosphorylation (WP)
Electron Transport Chain (WP)
Pyruvate metabolism (KEGG)
Respiratory electron transport (RA)
The citric acid (TCA) cycle and respiratory electron transport (RA)
Cell death signalling via NRAGE, NRIF and NADE (RA)
Respiratory electron transport, ATP synthesis by chemiosmotic coupling, and heat production by uncoupling proteins (RA)
G alpha (12/13) signalling events (RA)
Oxidative phosphorylation (KEGG)
Apelin signaling pathway (KEGG)
Relaxin signaling pathway (KEGG)
Chemokine signaling pathway (KEGG)
Vascular smooth muscle contraction (KEGG)
Myometrial Relaxation and Contraction Pathways (WP)
Oxytocin signaling pathway (KEGG)
Signaling by Hedgehog (RA)
Purine metabolism (KEGG)
Dilated cardiomyopathy (KEGG)
Muscle contraction (RA)
Cholinergic synapse (KEGG)
Cocaine addiction (KEGG)
p53 signaling pathway (KEGG)
p53 signaling (WP)
Transferrin endocytosis and recycling (RA)
D
Mitochondrial-related metabolic processes
Control
AAV-FGF21(a)
Pyruvate metabolism
TCA
BCCA degradation
E
Electron transport chain
Complex
z score
F
Cytosol
Mitochondria
Pyruvate metabolism
Fatty Acid Synthesis
Fatty acid metabolism
Fatty Acid Oxidation
TCA
Aminoacid metabolism
Electron Transport Chain
G
mt-Nd1 relative expression (FC)
0.149
H
iBAT
Fatty acid biosynthesis (KEGG)
Class C/3 (Metabotropic glutamate/pheromone receptors) (RA)
Inwardly rectifying K+ channels (RA)
Activation of G protein gated Potassium channels (RA)
G protein gated Potassium channels (RA)
Inhibition of voltage gated Ca2+ channels via Gbeta/gamma subunits (RA)
Aspirin ADME (RA)
Peroxisomal lipid metabolism (RA)
GABA B receptor activation (RA)
Activation of GABAB receptors (RA)
Synthesis of PA (RA)
GABA receptor activation (RA)
Splicing factor NOVA regulated synaptic proteins (WP)
Propanoate metabolism (KEGG)
GnRH secretion (KEGG)
Eukaryotic Translation Initiation (RA)
GTP hydrolysis and joining of the 60S ribosomal subunit (RA)
L13a-mediated translational silencing of Ceruloplasmin expression (RA)
Smooth Muscle Contraction (RA)
Nonsense Mediated Decay (NMD) independent of the Exon Junction Complex (EJC) (RA)
RHOU GTPase cycle (RA)
Formation of a pool of free 40S subunits (RA)
G-protein beta:gamma signalling (RA)
Translation initiation complex formation (RA)
Ribosomal scanning and start codon recognition (RA)
Cytoplasmic Ribosomal Proteins (WP)
SRP-dependent cotranslational protein targeting to membrane (RA)
Activation of the mRNA upon binding of the cap-binding complex and eIFs, and subsequent binding to 43S (RA)
Formation of the ternary complex, and subsequently, the 43S complex (RA)
Cell-extracellular matrix interactions (RA)
I
Ucp1 relative expression (FC)
0.071

Figure S3. Effects of treatment with AAV-FGF21 in adipose tissue in male mice. Male mice were treated with either AAV-Null (control) or AAV-FGF21 vectors at 13 months of age (AAV-FGF21 (a)). (**A**) Quantification by qRT–PCR of the expression of the inflammatory markers *Cd68*, *F4/80* and *Il1β* in iWAT at 21 months of age (n=5/group). (**B** and **C**) The plots represent the gene sets identified through Gene Set Enrichment Analysis (GSEA) of iWAT (B) and eWAT (C) of AAV-Null and AAV-FGF21-treated mice at 21 months of age. The color gradient from blue to red signifies the enrichment score (ES) values, providing a visual representation of enrichment magnitude. (**D** and **E**) The heatmaps depict the normalized expression patterns of leading-edge genes involved in mitochondrial-related metabolic processes (D) and encoding electron transport chain components (E) in eWAT at 21 months of age. (**F**) Scheme depicting the upregulated leading edge-genes (highlighted in red) involved in mitochondrial-related metabolism in iWAT of 21-month-old AAV-FGF21-treated mice. (**G**) Quantification by qRT–PCR of the expression of the mitochondrial gene mt-*Nd1* encoding the subunit 1 of NADH dehydrogenase in iWAT of 21-month-old mice (*n*=5/group). (**H**) The plot represents the gene sets identified through Gene Set Enrichment Analysis (GSEA) of iBAT of AAV-Null and AAV-FGF21-treated mice at 21 months of age. The color gradient from blue to red signifies the enrichment score (ES) values, providing a visual representation of enrichment magnitude. (**I**) Quantification by qRT–PCR of the expression of the thermogenic marker *Ucp1* in iBAT of 21-month-old mice (*n*=5/group). In (A,G,I) data are expressed as the mean ± SEM. FC, fold change. *P<0.05 by Student's two-tailed t test.

Figure S4

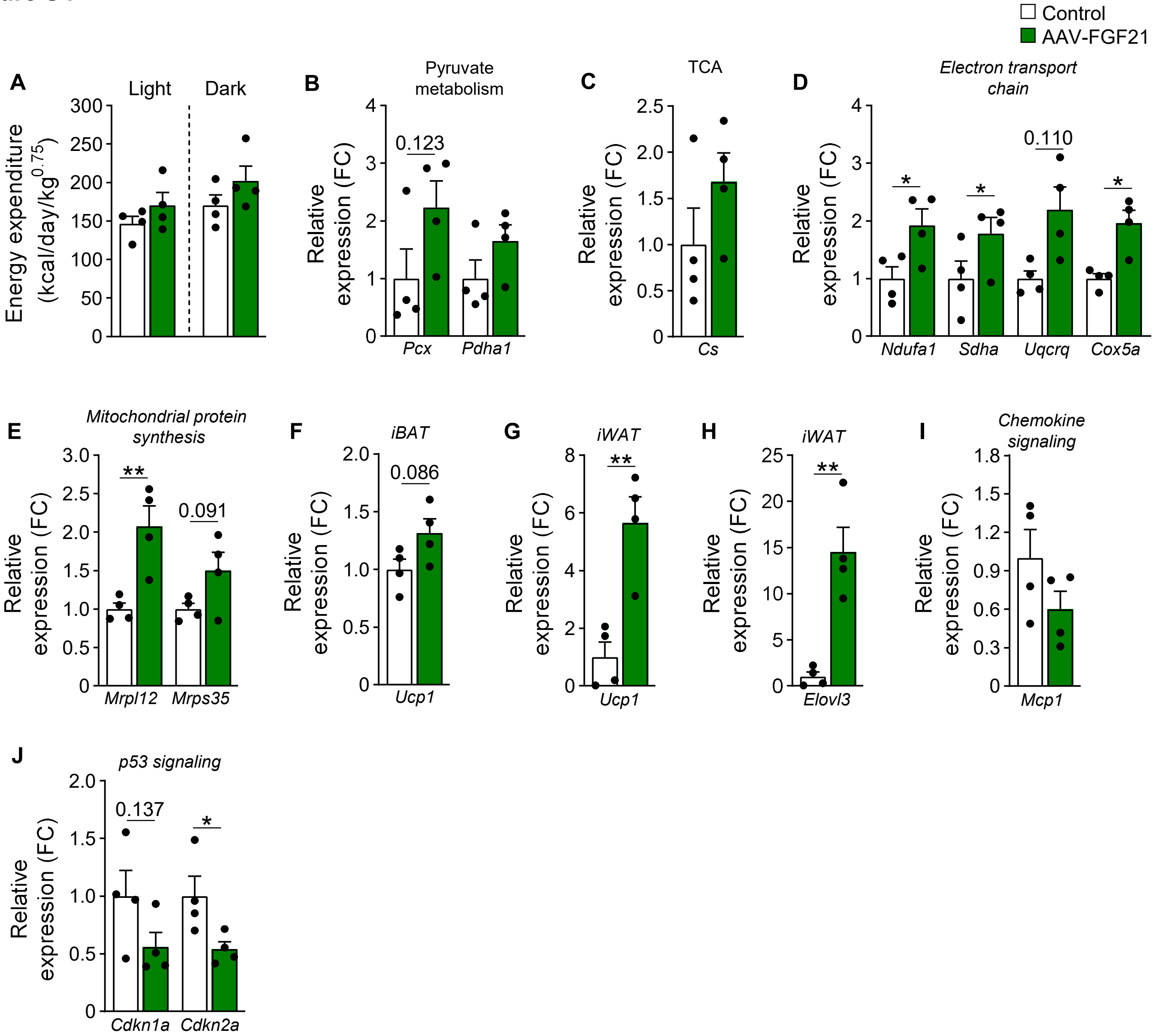

Control
AAV-FGF21
A
Light
Dark
Energy expenditure (kcal/day/kg$^{0.75}$)
B
Pyruvate metabolism
0.123
Relative expression (FC)
Pcx
Pdha1
C
TCA
Cs
D
Electron transport chain
0.110
Ndufa1
Sdha
Uqcrq
Cox5a
E
Mitochondrial protein synthesis
0.091
Mrpl12
Mrps35
F
iBAT
0.086
Ucp1
G
iWAT
Ucp1
H
iWAT
Elovl3
I
Chemokine signaling
Mcp1
J
p53 signaling
0.137
Cdkn1a
Cdkn2a

Figure S4. Effects of treatment with AAV-FGF21 in adipose tissue in female mice. (**A**) Energy expenditure was measured with an indirect open circuit calorimeter in female mice treated with AAV-FGF21 vectors at 19 months of age (*n*=4/group). (**B-E**) Quantification by qRT–PCR of the expression of key genes involved in pyruvate metabolism (B), the tricarboxylic acid cycle (TCA) (C), electron transport chain and mitochondrial protein synthesis (D) in iWAT in 25-month-old female mice (*n*=4/group). (**F** and **G**) Quantification by qRT–PCR of the expression of the thermogenic gene marker *Ucp1* in iBAT (F) and iWAT (G) in 25-month-old female mice (*n*=4/group). (**H**) Quantification by qRT–PCR of the expression of *Elovl3* in iWAT in 25-month-old female mice (*n*=4/group). (**I** and **J**) Quantification by qRT–PCR of the expression of key genes involved in chemokine (I) and p53 signaling (J) pathways in pmWAT in 25-month-old female mice (*n*=4/group). Data are expressed as the mean ± SEM. FC, fold change. *P<0.05, **P<0.01 by Student's two-tailed t test.

Figure S5

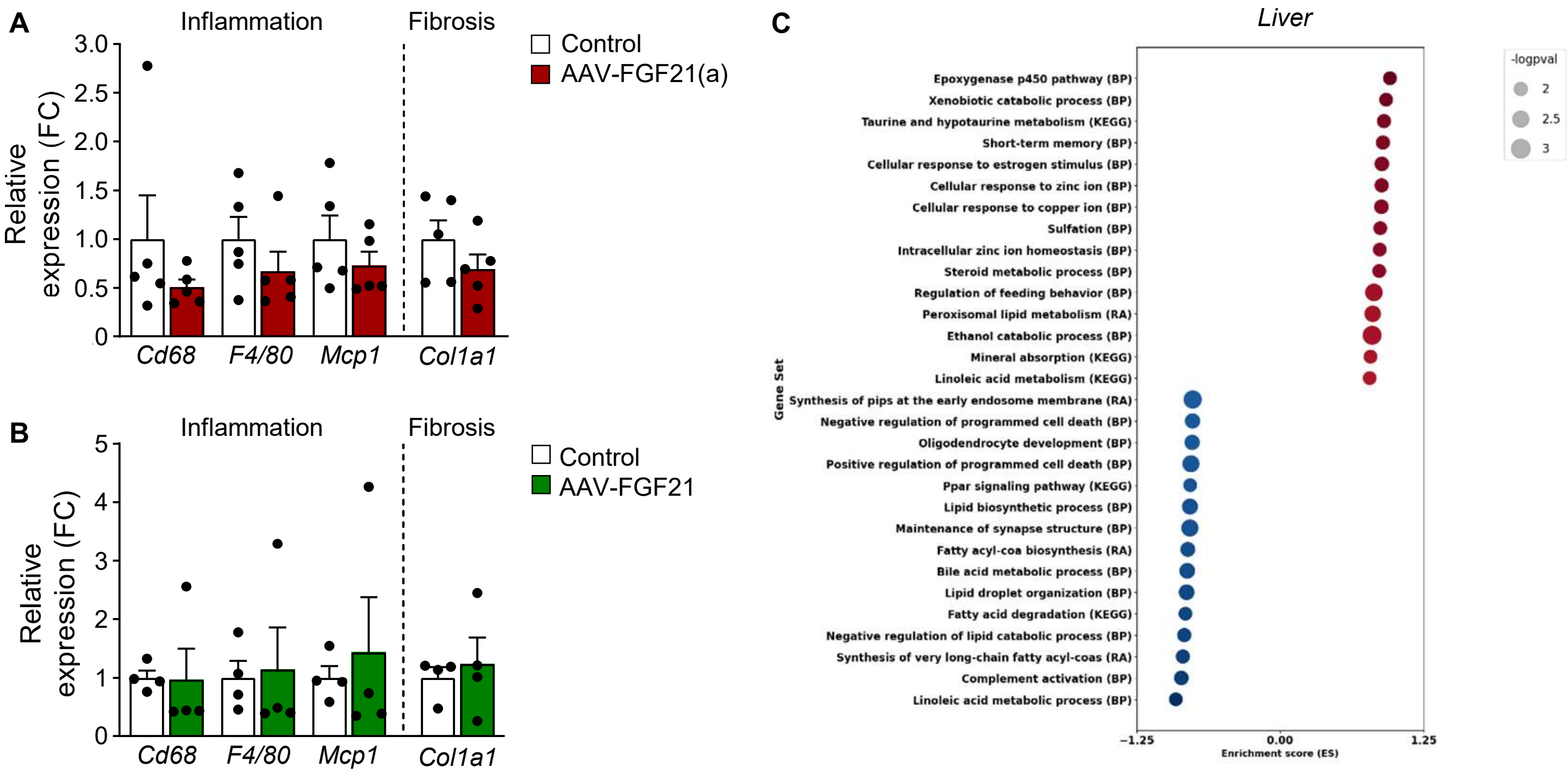

Figure S5. Effects of treatment with AAV-FGF21 in the liver. Male and female mice were treated with either AAV-Null (control) or AAV-FGF21 vectors at 13 (AAV-FGF21 (a)) or 19 months of age (AAV-FG21), respectively. (**A and B**) Quantification by qRT–PCR of the expression of the inflammatory and fibrosis markers *Cd68, F4/80*, *Mcp1* and *Col1a1* in the liver of male mice at 21 months of age (*n*=5/group) (A) or of female mice at 25 months of age (*n*=4/group) (B). (**C**) The plot represents the gene sets identified through Gene Set Enrichment Analysis (GSEA) of the liver of AAV-Null and AAV-FGF21-treated male mice at 21 months of age. The color gradient from blue to red signifies the ES values, providing a visual representation of enrichment magnitude. In (A,B) data are expressed as the mean ± SEM. FC, fold change.

Figure S6

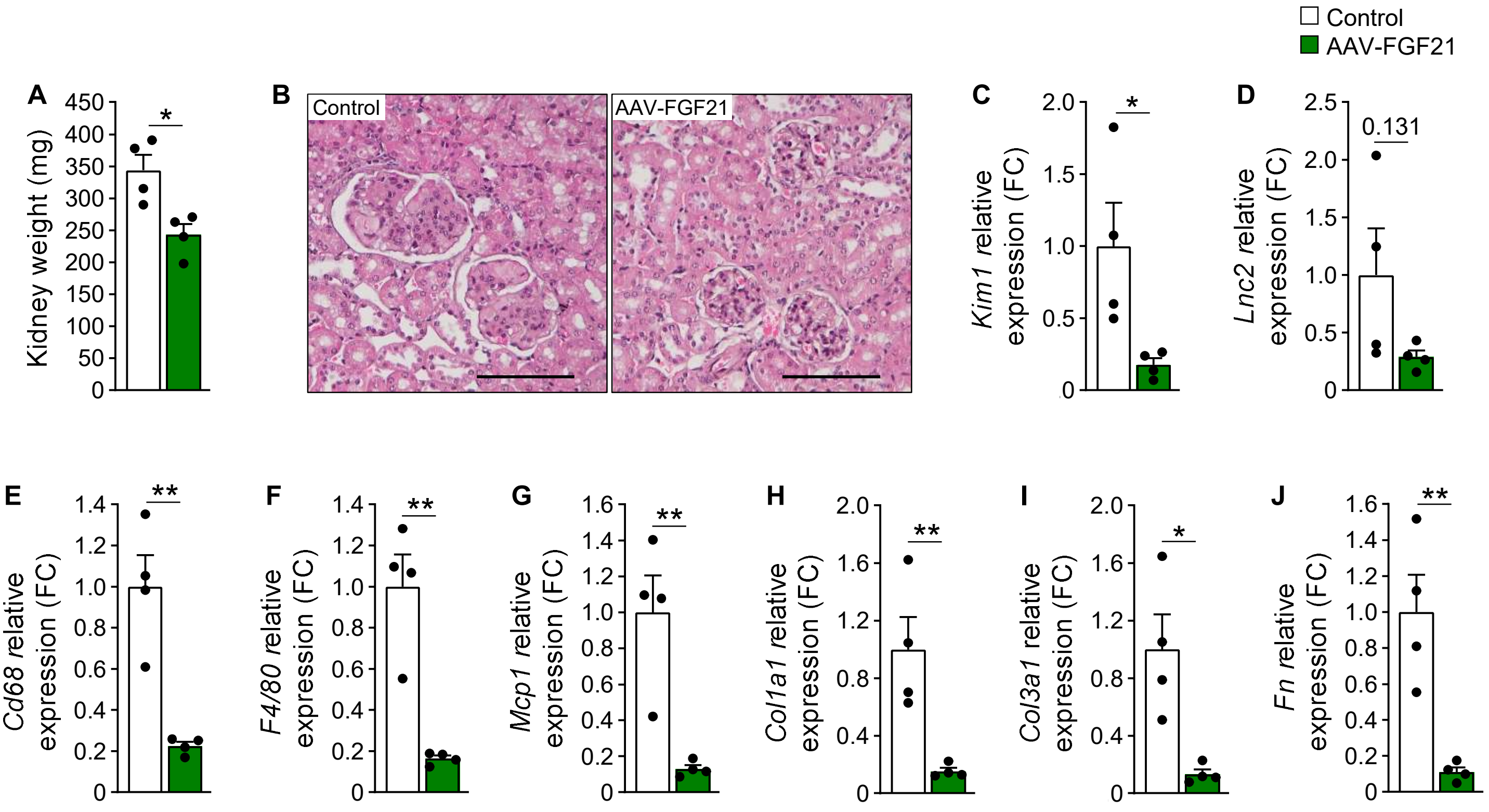

Figure S6. AAV-FGF21 treatment revers age-related kidney disease in female mice. Female mice were treated with AAV-FGF21 vectors at 19 (AAV-FGF21) months of age. (**A**) Kidney weight (n=4/group). (**B**) Representative images of hematoxylin–eosin staining of kidney sections. Scale bars, 100 µm. (**C** and **D**) Quantification by qRT–PCR of the expression of the kidney damage gene markers *Kim1* (C) and *Lnc2* (D) (n=4/group). (**E** to **J**) Quantification by qRT–PCR of the expression of the inflammatory and fibrosis gene markers *Cd68* (E), *F4/80* (F), *Mcp1* (G), *Col1a1* (H), *Col3a1* (I) and *Fn* (J) (n=4/group). Data are expressed as the mean ± SEM. *P<0.05, **P<0.01 by Student's two-tailed t test. FC, Fold change.

Figure S7

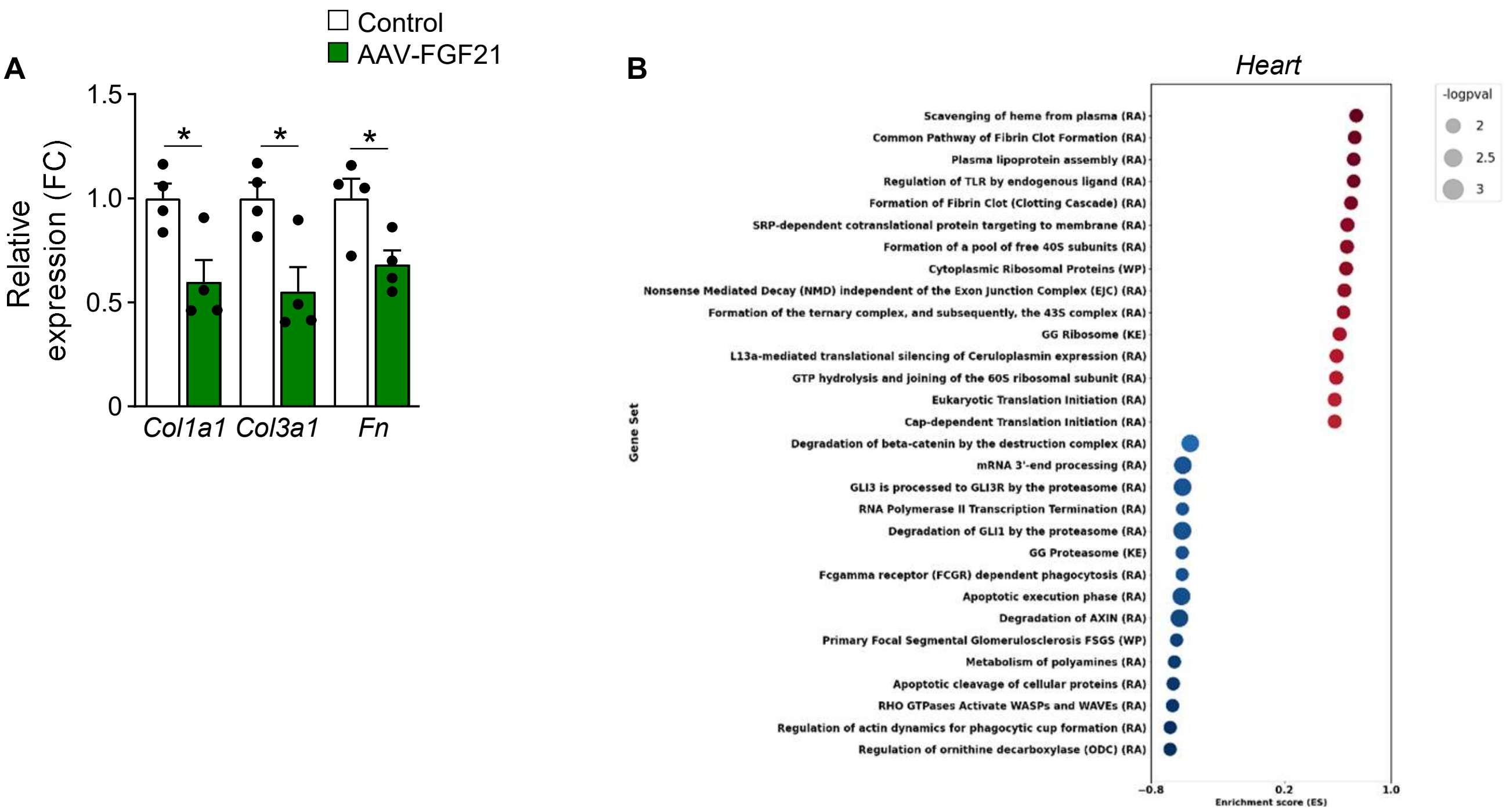

Figure S7. Effects of treatment with AAV-FGF21 in the heart. (**A**) Quantification by qRT–PCR of the expression of the fibrotic gene markers *Col1a1*, Col3a1, and *Fn*, (n=4/group) in female mice treated with AAV-FGF21 vectors at 19 months of age and sacrificed at 25 months of age. (**B**) The plot represents the gene sets identified through Gene Set Enrichment Analysis (GSEA) of the heart of AAV-Null and AAV-FGF21-treated mice at 21 months of age. The color gradient from blue to red signifies the ES values, providing a visual representation of enrichment magnitude. In (A) data are expressed as the mean ± SEM. *P<0.05 by Student's two-tailed t test. FC, Fold change.

Figure S8

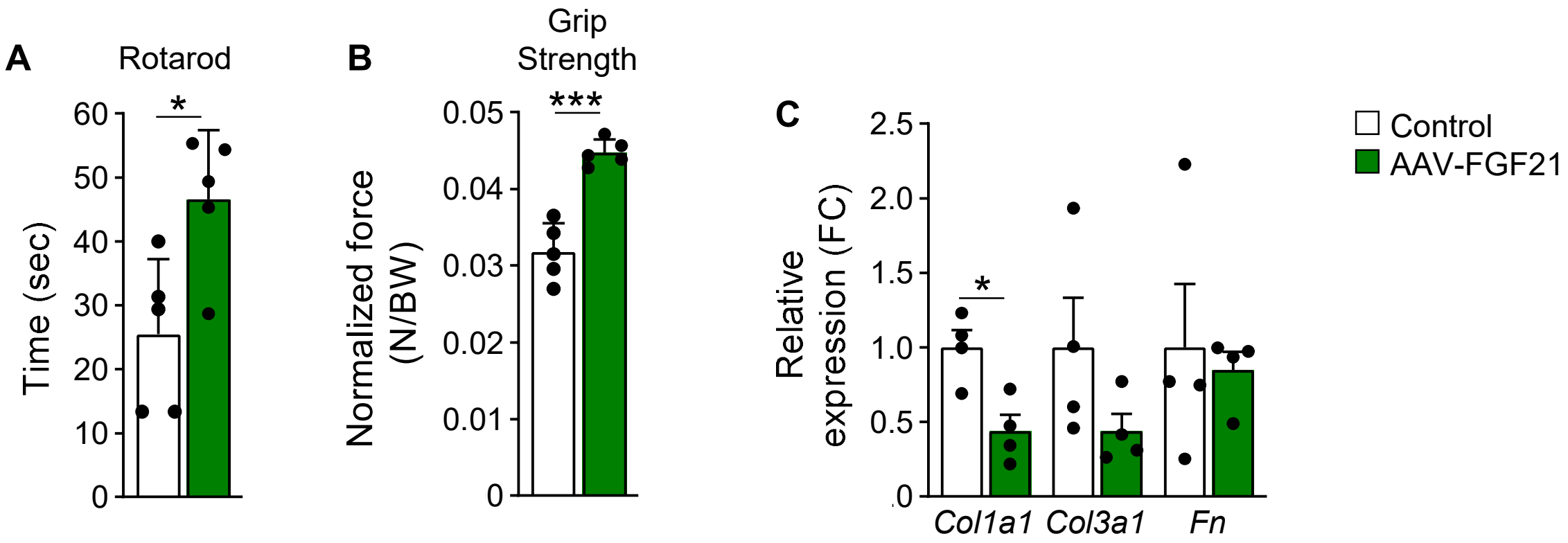

Figure S8. AAV1-FGF21 therapy improves muscular performance in female mice. (**A**) Time before falling during the rotarod test (*n*=5/group). (**B**) Forelimb grip strength (*n*=5/group). (**C**) Quantification by qRT–PCR of the expression of the fibrotic markers *Col1a1*, Col3a1, and *Fn* (n=4/group). Data are expressed as the mean ± SEM. *P<0.05, ***P<0.001 by Student's two-tailed t test.

Figure S9

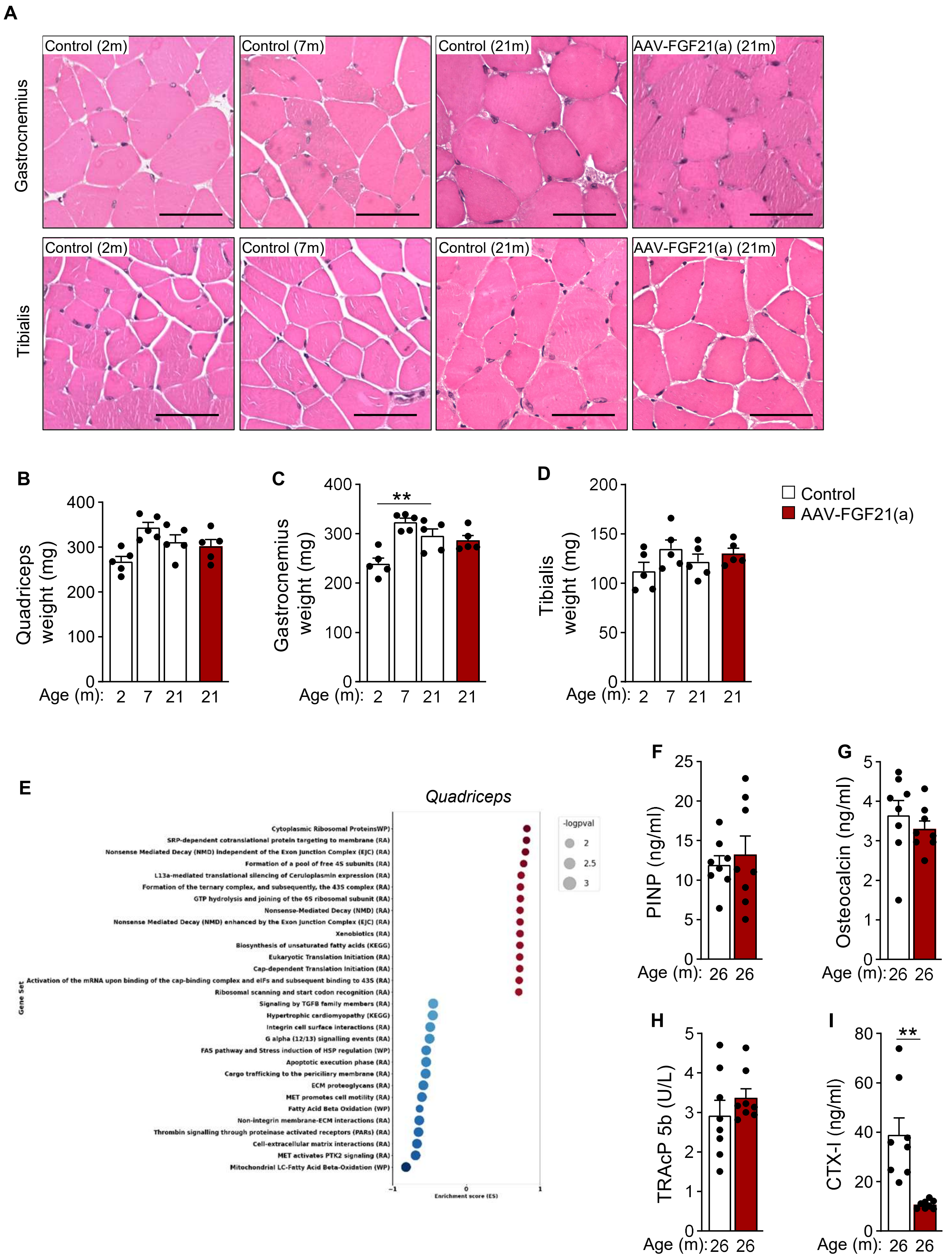

A
Gastrocnemius
Tibialis
Control (2m)
Control (7m)
Control (21m)
AAV-FGF21(a) (21m)
B
Quadriceps weight (mg)
C
Gastrocnemius weight (mg)
**
D
Tibialis weight (mg)
Age (m): 2 7 21 21
Control
AAV-FGF21(a)
E
Quadriceps
-logpval
2
2.5
3
Gene Set
Cytoplasmic Ribosomal ProteinsWP)
SRP-dependent cotranslational protein targeting to membrane (RA)
Nonsense Mediated Decay (NMD) independent of the Exon Junction Complex (EJC) (RA)
Formation of a pool of free 4S subunits (RA)
L13a-mediated translational silencing of Ceruloplasmin expression (RA)
Formation of the ternary complex, and subsequently, the 43S complex (RA)
GTP hydrolysis and joining of the 6S ribosomal subunit (RA)
Nonsense-Mediated Decay (NMD) (RA)
Nonsense Mediated Decay (NMD) enhanced by the Exon Junction Complex (EJC) (RA)
Xenobiotics (RA)
Biosynthesis of unsaturated fatty acids (KEGG)
Eukaryotic Translation Initiation (RA)
Cap-dependent Translation Initiation (RA)
Activation of the mRNA upon binding of the cap-binding complex and eIFs and subsequent binding to 43S (RA)
Ribosomal scanning and start codon recognition (RA)
Signaling by TGFB family members (RA)
Hypertrophic cardiomyopathy (KEGG)
Integrin cell surface interactions (RA)
G alpha (12/13) signalling events (RA)
FAS pathway and Stress induction of HSP regulation (WP)
Apoptotic execution phase (RA)
Cargo trafficking to the periciliary membrane (RA)
ECM proteoglycans (RA)
MET promotes cell motility (RA)
Fatty Acid Beta Oxidation (WP)
Non-integrin membrane-ECM interactions (RA)
Thrombin signalling through proteinase activated receptors (PARs) (RA)
Cell-extracellular matrix interactions (RA)
MET activates PTK2 signaling (RA)
Mitochondrial LC-Fatty Acid Beta-Oxidation (WP)
Enrichment score (ES)
F
PINP (ng/ml)
G
Osteocalcin (ng/ml)
H
TRAcP 5b (U/L)
I
CTX-I (ng/ml)
**
Age (m): 26 26

Figure S9. Effects of treatment with AAV-FGF21 in the skeletal muscle and bones. Male mice were treated with either AAV-Null (control) or AAV-FGF21 vectors at 13 months of age (AAV-FGF21 (a)). (**A**) Representative images of hematoxylin–eosin staining of gastrocnemius and tibialis cranialis sections. Scale bars, 100 µm. (**B**-**D**) Weight of the quadriceps (B), gastrocnemius (C) and tibialis cranialis (D) skeletal muscles (*n*=5/group). (**E**) The plot represents the gene sets identified through Gene Set Enrichment Analysis (GSEA) of the quadriceps of AAV-Null and AAV-FGF21-treated mice at 21 months of age. The color gradient from blue to red signifies the ES values, providing a visual representation of enrichment magnitude. (**F** and **G**) Serum levels of the bone formation markers procollagen type 1 N-terminal propeptide (PINP) (F) and osteocalcin (G) in 26-month-old male mice. (**H** and **I**) Serum levels of the bone resorption markers tartrate-resistant acid phosphatase 5b (TRAcP 5b) (H) and circulating carboxy-terminal crosslinked telopeptide of type 1 collagen (CTX-1) (I) in 26-month-old male mice. In (B-D and F-I) data are expressed as the mean ± SEM. **P<0.01 by one-way ANOVA with Dunnet's multiple comparison test (B-D) or by Student's two-tailed t test (F-I). m, months of age.

Figure S10

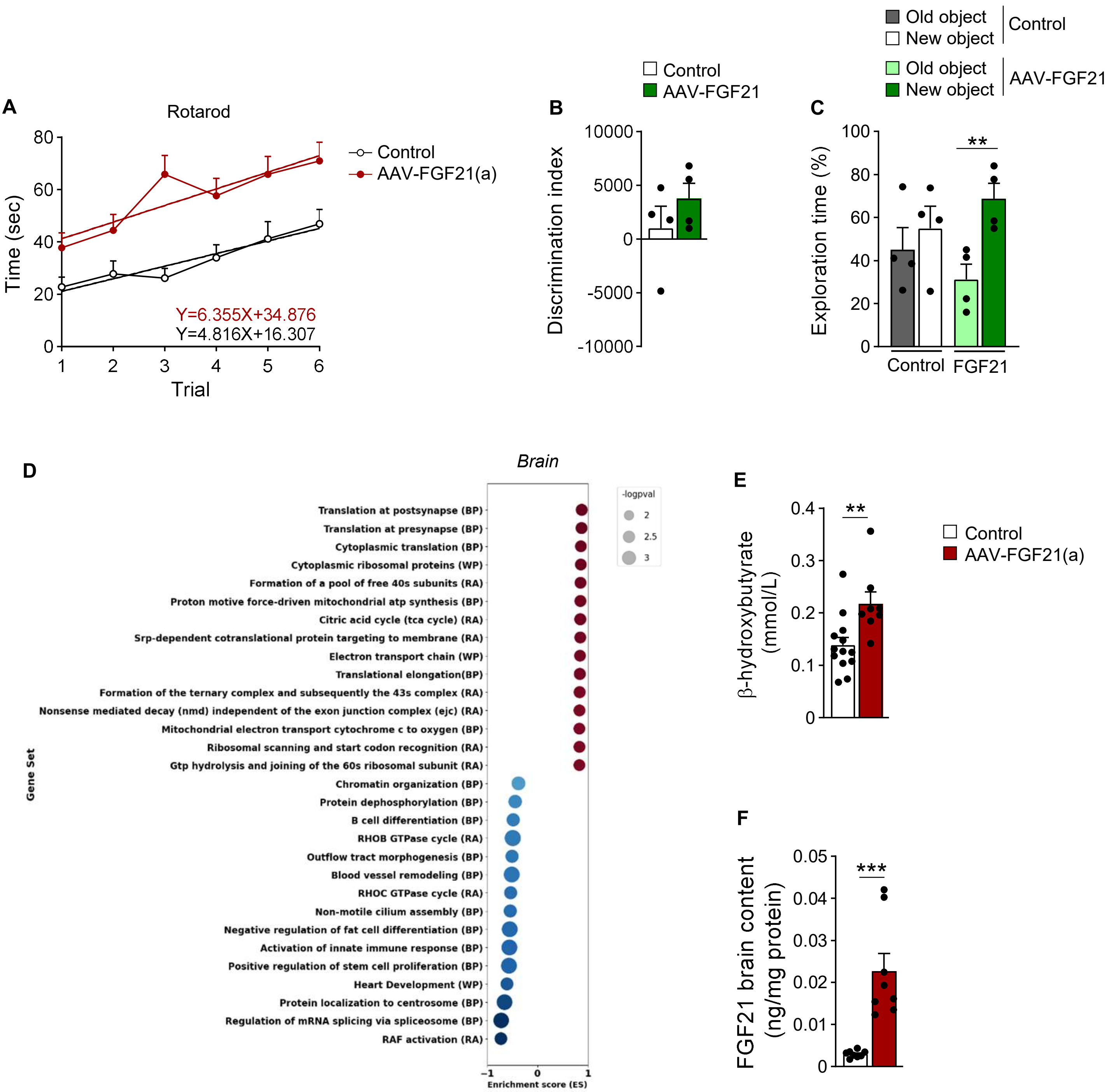

A
Rotarod
Time (sec)
Trial
Control
AAV-FGF21(a)
Y=6.355X+34.876
Y=4.816X+16.307
B
Control
AAV-FGF21
Discrimination index
C
Old object
New object
Control
Old object
New object
AAV-FGF21
Exploration time (%)
**
Control
FGF21
D
Brain
-logpval
Gene Set
Translation at postsynapse (BP)
Translation at presynapse (BP)
Cytoplasmic translation (BP)
Cytoplasmic ribosomal proteins (WP)
Formation of a pool of free 40s subunits (RA)
Proton motive force-driven mitochondrial atp synthesis (BP)
Citric acid cycle (tca cycle) (RA)
Srp-dependent cotranslational protein targeting to membrane (RA)
Electron transport chain (WP)
Translational elongation(BP)
Formation of the ternary complex and subsequently the 43s complex (RA)
Nonsense mediated decay (nmd) independent of the exon junction complex (ejc) (RA)
Mitochondrial electron transport cytochrome c to oxygen (BP)
Ribosomal scanning and start codon recognition (RA)
Gtp hydrolysis and joining of the 60s ribosomal subunit (RA)
Chromatin organization (BP)
Protein dephosphorylation (BP)
B cell differentiation (BP)
RHOB GTPase cycle (RA)
Outflow tract morphogenesis (BP)
Blood vessel remodeling (BP)
RHOC GTPase cycle (RA)
Non-motile cilium assembly (BP)
Negative regulation of fat cell differentiation (BP)
Activation of innate immune response (BP)
Positive regulation of stem cell proliferation (BP)
Heart Development (WP)
Protein localization to centrosome (BP)
Regulation of mRNA splicing via spliceosome (BP)
RAF activation (RA)
Enrichment score (ES)
E
β-hydroxybutyrate (mmol/L)
**
Control
AAV-FGF21(a)
F
FGF21 brain content (ng/mg protein)
***

Figure S10. Effects of treatment with AAV-FGF21 vectors in the brain. (**A**) Motor learning ability during subsequent trials in the rotarod test in male mice treated with either AAV-Null (control) or AAV-FGF21 vectors at 13 months of age (AAV-FGF21 (a)) (n=14-15/group). (**B** and **C**). Discrimination index (B) and exploration time (C) during the novel object recognition (NOR) test in female mice treated with AAV-FGF21 vectors at 19 months of age (n=4/group). (**D**) The plot represents the gene sets identified through Gene Set Enrichment Analysis (GSEA) of the brain of AAV-Null and AAV-FGF21-treated male mice at 21 months of age. The color gradient from blue to red signifies the ES values, providing a visual representation of enrichment magnitude. (**E**) Serum β-hydroxybutyrate levels in male mice at 26 months age (*n*=8-13/group). (**F**) FGF21 protein content in brain in male mice at 21 months of age (*n*=8/group). In (A,B,C,E,F) data are expressed as the mean ± SEM. **P<0.01, ***P<0.001 by Student's two-tailed t test.

Table S1. Annotated sequence of the pAAV-CMV-moFGF21 plasmid.

Table S2. GSEA Leading Edge Analysis and Cluster Map across Tissues. The dataset presents the top 15 enriched gene sets for AAV-FGF21- (orange) and AAV-Null-treated (blue) mice, categorized by tissue type in individual sheets. For each gene set, the leading-edge genes (the subset driving the enrichment score) are identified. The accompanying binary cluster maps (overlap matrices) visualize the distribution of these genes (x-axis) across the enriched pathways (y-axis). Yellow blocks indicate gene presence within a pathway's leading edge, while purple indicates absence. This matrix identifies "hub" genes contributing to multiple biological processes and highlights functional redundancies between enriched gene sets, facilitating a modular understanding of the FGF21-induced transcriptional response.
